# The impact of weight loss interventions on disordered eating symptoms in people with overweight and obesity: a systematic review & meta-analysis

**DOI:** 10.1016/j.eclinm.2024.103049

**Published:** 2025-01-31

**Authors:** Elena Tsompanaki, Dimitrios A. Koutoukidis, Gina Wren, Heather Tong, Annika Theodoulou, Danni Wang, Rebecca J. Park, Susan A. Jebb, Paul Aveyard

**Affiliations:** a Nuffield Department of Primary Care Health Sciences, University of Oxford, Oxford, United Kingdom; b Department of Psychiatry, University of Oxford, Oxford, United Kingdom

**Keywords:** Weight loss programmes, Disordered eating, Overweight/obesity

## Abstract

**Background:**

It is unclear whether weight loss interventions worsen disordered eating in people living with overweight/obesity. We aimed to systematically evaluate the association between weight loss interventions and disordered eating.

**Methods:**

Six databases were searched from inception until September 2024. Trials of weight loss interventions in people with overweight/obesity were included if they reported a validated score for disordered eating on either the Eating Disorder Examination Interview or the Eating Disorder Examination Questionnaire pre- and post-intervention. Interventions included behavioural weight loss programmes (BWL) and pharmacotherapy licenced for weight loss, with or without concurrent psychological support, provided for at least 4 weeks. Pooled standardised mean differences (SMD) in scores of disordered eating were calculated using random effects meta-analyses. Risk of bias (RoB) was assessed using the Cochrane RoB 2 tool and the Newcastle–Ottawa scale for randomised and single-arm trials, respectively (PROSPERO ID: CRD42023404792).

**Findings:**

Thirty-eight studies with 66 eligible arms (61 interventions: 29 BWL, 11 BWL + pharmacotherapy, 20 BWL + psychological intervention, 1 pharmacotherapy + psychological intervention) and 3364 participants in total were included. The mean weight change was −4.7 kg (95% CI: −5.7, −3.7). Compared with baseline, disordered eating scores improved by −1.47 SMD units (95% CI: −1.67, −1.27, p < 0.001, I^2^ = 94%) at intervention completion (median of 4 months). Seven randomised trials that directly compared a weight loss intervention to no/minimal intervention reported an improvement of −0.49 SMD units (95% CI, −0.93, −0.04, p = 0.0035, I^2^ = 73%). Sub-group analyses showed: (a) disordered eating scores improved more in people with an eating disorder at baseline compared with people without high scores, (b) no clear evidence that the association depended upon intervention type, and (c) disordered eating scores improved more in trials rated at low overall RoB.

**Interpretation:**

Despite heterogeneity in effect size, weight loss interventions consistently improved disordered eating scores. These findings provide reassurance that weight loss interventions might not worsen disordered eating and may improve it.

**Funding:**

10.13039/501100000329Novo Nordisk UK Research Foundation Doctoral Fellowship in Clinical Diabetes.

**Research in context**

**Evidence before this study**

Concerns have been expressed that intentional weight loss may cause or exacerbate disordered eating. There is some limited evidence from previous systematic reviews that behavioural weight loss programmes either do not change or reduce disordered eating, but previous reviews have excluded some studies enrolling people with eating disorders or interventions with pharmacotherapy.

**Added value of this study**

In people living with overweight/obesity, behavioural weight loss interventions were associated with significant improvements in disordered eating scores at the end of the intervention and for shortly after, regardless of baseline disordered eating scores. The improvement in scores was greater in the pre-post analysis of single arm trials but was also evident in the meta-analysis of the randomised controlled trials. It was also greater in people with higher eating disorder scores and in studies appraised as at low risk of bias.

**Implications of all the available evidence**

People living with overweight/obesity enroling in interventions intended to promote weight loss consistently experience improvements in their symptoms of disordered eating in the short-term and there is modest evidence from trials that this is causal. Weight loss programmes reduce risk factors for, incidence of, and mortality from cardiovascular disease and other problems of excess adiposity. This data should provide reassurance to practitioners to offer weight-loss treatments, even to people living with an eating disorder to improve their health.

## Introduction

Eating disorders have amongst the highest morbidity and mortality of any psychiatric disorder,^1^ incurring major costs to individuals and health care systems. The global prevalence of eating disorders is increasing, with a lifetime prevalence of around 8% for women and 2% for men and an apparent doubling in prevalence from the early 2000s.^2^

The prevalence of obesity in adults has also increased, doubling since 1990; an estimated 250 billion adults globally were affected by overweight/obesity in 2022.^3^ Excess weight increases the incidence of many non-communicable diseases, such as type 2 diabetes (T2D), cardiovascular disease^4^ and several cancers.^5^ Intentional weight loss improves cardiometabolic risk factors and reduces morbidity and mortality.^6^^,^^7^ Behavioural weight loss programmes (BWL), surgery, medication or combinations of these treatments can support people to lose weight.^8^ Weight loss can be pursued both in the context of a led or self-guided programme.

Eating disorders, in particular involving binge eating, are more prevalent amongst people with obesity when compared to people with healthy weight.^9^ One possible explanation for this co-occurrence is that attempts at intentional weight loss could lead to eating disorders in those vulnerable. Body dissatisfaction can lead to dietary restraint, with lapses in control leading to guilt, lowered self-esteem, and binge eating, thereby redoubling restraint.^10^ An alternative or complementary explanation is that shared risk factors, including genetic and psychological factors, could account for this co-occurrence. Data on whether weight loss interventions increase or decrease the incidence of eating disorders or disordered eating would help resolve this uncertainty. Several systematic reviews have provided imprecise evidence suggesting a potential benefit of participating in BWL programmes on eating disorder symptoms. However, these reviews excluded studies with interventions targeting eating disorders alongside weight loss or using weight loss pharmacotherapy or were not able to conduct meta-analyses.^11^^,^^12^ Accordingly, we undertook a systematic review to assess the impact of weight loss interventions, with or without pharmacotherapy, on disordered eating symptoms, regardless of baseline disordered eating status or type of weight loss intervention.

## Methods

The review protocol was prospectively registered on the International Prospective Register of Systematic Reviews (PROSPERO, ID: CRD42023404792).^13^ The review followed the Preferred Reporting Items for Systematic Reviews and Meta-Analysed (PRISMA) reporting guidelines.^14^

### Eligibility criteria

Randomised controlled, randomised comparative, and single-arm trials of weight loss interventions were included if they recruited adults (≥18 years) with overweight/obesity (as defined in the original publications), with or without disordered eating or eating disorders at baseline. Studies that included participants with both healthy and excess weight were included only if they reported results in the subgroup of those with overweight/obesity at baseline. BWL programmes had to prescribe energy restriction with an explicit weight loss goal.^15^ Psychological interventions had to include widely used psychological treatments aimed at weight loss and lasting at least 4 weeks, like cognitive behavioural therapy (CBT), or interventions developed and/or delivered by psychologists/therapists/mental health professionals (or relevant students). Pharmacotherapy interventions were those licensed for obesity treatment (or those that share a class effect) in the UK. Behavioural weight loss programmes could be combined with pharmacotherapy and/or psychological support to manage disordered eating and thereby reduce weight. Interventions focusing solely on exercise/physical activity without energy restriction were excluded. There was no restriction on the length of follow-up of participants.

Studies were required to report disordered eating in either a global score and/or sub-scale scores using the Eating Disorder Examination (EDE) Interview or the Eating Disorder Examination Questionnaire (EDE-Q), and we extracted all available scores. The EDE, and the questionnaire derived from it, EDE-Q, are considered the gold standard^16^ and they both have been validated among clinical and community samples.^17^, ^18^, ^19^

For comparative trials, the comparators could be usual care, minimal intensity interventions for weight loss, or waiting list control. Usual care was determined in the context of what is usually offered in routine care by non-specialist staff (e.g. publicly available resources on diet and weight loss). If studies had arms described as “usual care” but offered specialist advice and/or referral to structured programmes, these were included as active interventions. Studies in animals, children and/or adolescents, on people with obesity due to genetic conditions (e.g. Prader–Willi syndrome), or after bariatric surgery, or pharmacotherapy used off-label for weight loss (e.g. antidepressants not licenced for weight loss) were excluded.

### Process

MEDLINE, Embase, PsycINFO, CINAHL, Cochrane Central Register of Controlled Trials and Web of Science were searched from inception until 30/09/2024, using relevant terminologies to capture: “weight loss/weight management”, “obesity/overweight”, “disordered eating/eating disorders”. The search strategy can be found in the Supplementary Materials, section B. Searches were not restricted by country or language. References both within systematic reviews identified through searches, and of the studies included in this review, were screened.

The Covidence software was used for abstract and full-text screening.^20^ Extraction was performed into a pilot-tested and bespoke extraction sheet in Microsoft Excel. Five independent reviewers (ET, GW/HT, DW/AT) screened titles and abstracts, and full texts for inclusion. Two reviewers (ET, HT) independently extracted data, and two independent reviewers (GW, AT) checked 50% of extractions for accuracy. Any conflicts in screening or extraction were resolved by discussion, or by referral to a third reviewer (DAK). Study authors were contacted for further data and/or clarifications, if needed. The outcomes at end of intervention and post-intervention follow-up were extracted where the latter took place at least six weeks after end of intervention. Body weight was also collected, when available.

Risk of bias for randomised clinical trials was assessed by two independent reviewers (ET, GW), using the Cochrane risk of bias tool^21^ and the Newcastle Ottawa scale for observational studies.^22^ Risk of bias was assessed based on randomisation sequence generation, allocation concealment, blinding of outcome assessment, and attrition bias. It is not possible to blind participants or study personnel in behavioural intervention trials, therefore this domain was not assessed. Studies were judged at high risk of attrition if fewer than 75% of participants were followed up at the end of the intervention. Blinding of outcome assessment was judged as high risk if EDE interviews were done by study members who were aware of the participants’ allocation. Outcomes captured through self-reported EDE-Q were deemed low risk, as completion could not be influenced by the presence of a researcher. We assessed publication bias with funnel plots.

### Statistics

Given eligible studies included both EDE-Q and EDE scores, we calculated the standardised mean difference by dividing the difference in the mean disordered eating score pre- and post-intervention by the standard deviation of the change.^23^ When estimating the SD of change, we assumed the baseline and follow-up values were correlated based on published data.^24^ We repeated this process for the EDE-Q/EDE subscales. In some studies, only mean subscale scores were reported, without the mean global score; we therefore calculated the mean global score by dividing the sum of the mean subscale scores by the number of subscales. We conducted a post-hoc sensitivity analysis excluding these studies. We included analyses that imputed missing data where available. To reduce bias in summary estimates, we included studies regardless of whether the outcome showed significant change at follow-up.

Where studies only reported body mass index (BMI), we calculated weight using mean height by sex for the specific country.^25^

The pooled estimates were based on outcomes of similar endpoints and the pooled estimates are presented as mean differences with 95% confidence intervals (CI) for continuous variables. The minimum number of studies for quantitative synthesis in a meta-analysis was set at three. Statistical heterogeneity was assessed using the I^2^ statistic tool,^26^ with 0–24% indicating no heterogeneity, 25–49% low, 50–74% moderate and 75–100% high heterogeneity.

We used random-effects models because of the expected heterogeneity in the interventions and study populations. We conducted a meta-analysis for single arm trials pre and post intervention and a separate meta-analysis pooling randomised control trials comparing weight loss interventions with no active/minimal treatment, using the Hartung–Knapp–Sidik–Jonkman approach applied to the DerSimonian–Laird simple method of moments estimator to obtain less biased estimated effects and 95% CIs.^27^ For 3-arm RCTs, we compared the participants in each of the two interventions against half of the participants in the control group. Outcomes are summarised as difference in means with 95% CIs, both for SMD in disordered eating scores as well as weight in kg.

We ran two pre-specified sub-group analyses: (1) a sub-group analysis based on type of intervention (BWL versus BWL + pharmacotherapy versus BWL + psychological support) and (2) a subgroup analysis based on the baseline status of disordered eating. For the latter, we classed baseline disordered eating based on reported eligibility criteria as people with or without eating disorders. If not specified we classified the population as not having an eating disorder, if the EDE/EDE-Q score was <2.67, or as unselected for eating disorders, if it was at least 2.67.^28^ We undertook a post-hoc subgroup analysis based on overall risk of bias score. We conducted a meta-regression of the association between weight change and change in disordered eating scores. All analyses were conducted in R Studio, version 4.3.3.^29^

### Ethics

Ethical approval to conduct this work was not required.

### Role of funding source

The funder had no role in the design and conduct of this study; collection, management, analysis, and interpretation of the data; preparation, review, or approval of the manuscript; and decision to submit the manuscript for publication.

## Results

**Fig. 1:**
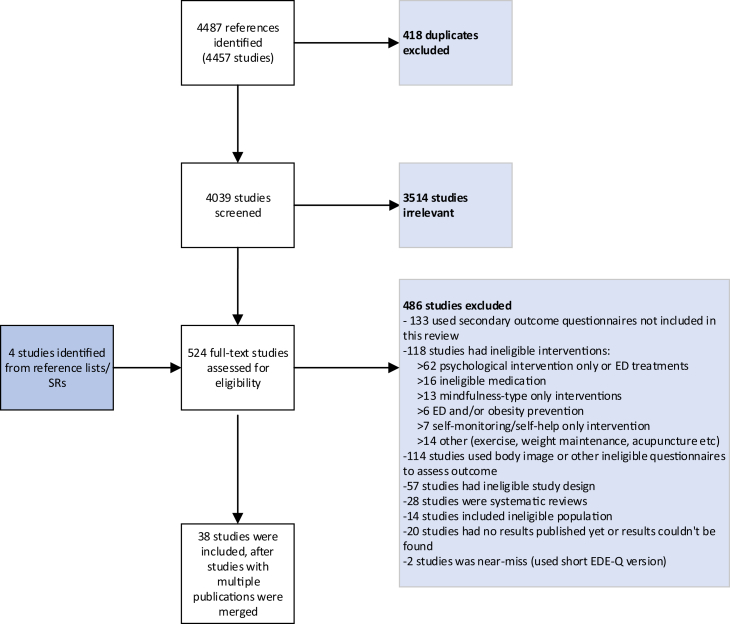
PRISMA flow diagram.

The literature search identified 4487 records and 524 references were full-text screened, with 38 studies included (Fig. 1). Twenty studies were ongoing and/or had not published results prior to October 2024.

The 38 trials included 3364 participants at baseline and reported on 66 eligible arms, including 10 randomised controlled trial arms, plus their 7 respective control groups. The number of participants per intervention arm ranged from 11 to 167, with a mean of 40 per arm. Fourteen studies were conducted in Europe and the United Kingdom (UK), 21 in North America, two in South America and one in Australia and New Zealand (Table 1, Supplementary Materials, section C).

**Table 1:** Characteristics of the included studies.

Study & country	Total, no (at baseline)	Arms	Presence of disordered eating at baseline	Outcome assessment tool	Duration, mo	Next follow-up post-intervention completion
Berk et al., 2018^30^ The Netherlands	83	Very low-calorie diet (VLCD) + cognitive behavioural therapy (CBT)	No	EDE-Q	18 m (higher intensity up till ∼6 months)	24 months
	75	VLCD				
Grilo et al., 2005^31^ USA	25	Orlistat + CBT *(ED treatment)*	Yes	EDE, subscales	3 months	6 months
	25	CBT + moderate weight loss diet *(ED treatment)*			3 months	
Wilfley et al., 2002^32^ USA	81	CBT and mild weight loss therapy *(ED treatment)*	Yes	EDE (manually converted by sub-scales), subscales	6 months	10 months
Calugi et al., 2016^33^ Italy	54	Residential weight loss therapy + CBT	No	EDE, subscales	∼1 month intensive and then as outpatient (in total 6 months)	N/A
	54 (BED)		Yes			
Carbone et al., 2021^34^ Italy	23 (BED)	Naltrexone-Bupropion + mild weight loss diet *(treatment for altered eating behaviour + weight loss)*	Yes	EDE-Q, subscales	4 months	N/A
	20		No			
Carels et al., 2021^35^ USA	26	Behavioural weight loss + self compassion skills training	No	EDE –Q (manually converted by sub-scales), subscales	3 months	N/A
	28	Behavioural weight loss				
Chao et al., 2019^36^ USA	50	Behavioural weight loss therapy	Population unselected by ED	EDE-Q, subscales	6 months	12 months
	50	Intensive Behavioural weight loss therapy + liraglutide				
	50	Intensive Behavioural weight loss therapy with 12-week low calorie diet + liraglutide				
daLuz et al., 2017^37^ Brazil	50	Mild weight loss + enhanced CBT *(ED treatment)*	Yes	EDE-Q	6 months	12 months
DalleGrave et al., 2020^38^ Italy	67	Weight loss + group CBT	No	EDE-Q	18 months	N/A
Mensinger et al., 2016^39^ USA	40	Weight loss programme	No	EDE-Q, subscales	6 months	24 months
Di Marco et al., 2009^40^ USA	20	Standard guided self-help weight loss + motivational interviewing	No	EDE-Q subscales, EDE-Q (manually converted by sub-scales)	3 months	N/A
	19	Standard guided self-help weight loss				
Grilo et al., 2021^41^ USA	12	Naltrexone and buproprion + lifestyle modification	Yes	EDE-Q	3 months	6 months
	10	control (placebo)				
Grilo et al., 2005^42^ USA	38	Behavioural weight loss + guided self help *(ED treatment)*	Yes	EDE-Q subscales, EDE-Q (manually converted by sub-scales)	3 months	N/A
	15	Control (minimal intervention)				
Dassen et al., 2018^43^ the Netherlands	51	Weight loss + cognitive training	No	EDE-Q	∼1 month	7 months
	40	Mild weight loss				
Grilo et al., 2013^44^ USA	20 (BED)	Orlistat + behavioural weight loss	Yes	EDE, subscales	4 months	6 months
	20 (BED)	Behavioural weight loss + placebo				
	20	Orlistat + behavioural weight loss	No			
	19	Behavioural weight loss + placebo				
Abiles et al., 2013^45^ Spain	49 (BED)	Mild weight loss + CBT	Yes	EDE-Q subscales, EDE-Q (manually converted by sub-scales)	3 + 12 months	N/A
	61 (non-BED)		No			
Grilo et al., 2020^46^ USA	47	Moderate weight loss therapy + orlistat	Yes	EDE	6 months	N/A
	46	Moderate weight loss therapy + placebo				
	39	Moderate weight loss therapy				
Loader et al., 2013^47^ UK	18	Behavioural weight loss + guided bibliotherapy self -elp	Population unselected by ED	EDE-Q subscales, EDE-Q (manually converted by sub-scales)	6 months	N/A
	18	Behavioural weight loss				
Nauta et al., 2000^48^ the Netherlands	21	Behavioural weight loss	No	EDE-Q subscales, EDE-Q (manually converted by sub-scales)	∼4 months	10 months
	16 (BED)		Yes			
Grilo et al., 2011^49^ USA	45	Behavioural weight loss	Yes	EDE, EDE subscales	6 months	12 months
	35	Sequential CBT + behavioral weight loss *(ED treatment)*			10 months	
Masheb et al., 2011^50^ USA	25	Low-energy density diet *+ CBT (ED treatment)*	Yes	EDE, EDE subscales	6 months	N/A
	25	Nutrition councelling + CBT *(ED treatment)*			6 months	
Munsch et al., 2007^51^ Switzerland	36	Behavioural weight loss therapy	Yes	EDE subscales, EDE (manually converted by sub-scales)	4 months	N/A
Pataky et al., 2018^52^ Switzerland	35	Multidisciplinary weight loss programme + CBT/coaching	Population unselected by ED	EDE-Q, subscales	12 months	N/A
	39					
	13					
Preuss et al., 2017^53^ Germany	28	Weight loss therapy with impulse control	Population unselected by ED	EDE-Q	∼3 months	6 months
Ramirez et al., 2001^54^ USA	40	Weight loss pogramme	No	EDE-Q Restraint and Eating Concern only	4 months	N/A
	48	Weight loss + body image therapy				
Rock et al., 2010^55^ USA	167	Weight loss programme (telephone)	No	EDE	6 months more intensive phase, 2 years in total	12 months
	164	Weight loss programme (face-to-face)				
	111	Control (Usual care)			6 months	
Barnes et al., 2017^56^ USA	30	Weight loss with motivational interviewing (online)	No	EDE-Q	3 months	6 months
	29	Nutritional psychoeducation				
	30	Control (usual care)				
Barnes et al., 2018^57^ USA	31	Motivational Interviewing and Nutrition Psychoeducation	No	EDE-Q	3 months	6 months
Moss et al., 2017^24^ Canada	69	Behavioral weight-loss programme + motivational interviewing	No	EDE-Q, subscales	3 months	9 months
	66	Behavioral weight-loss programme				
Werrij et al., 2009^58^ the Netherlands	96	Cogntitive dietetic group treatment	No	EDE-Q, subscales	2.5 months	12 months
	104	Exercise and dietetic treatment				
Wilson et al., 2010^59^ USA	64	Behavioural weight loss	Yes	EDE	6 months	12 months
Allison et al., 2022^60^ USA	11	Liragutide + lifestyle modification	Yes	EDE-Q	4 months	N/A
	12	Control (placebo)				
Grilo et al., 2022^61^ USA	35	Behavioural weight loss + placebo	Yes	EDE-Q	4 months	N/A
	35	Behavioural weight loss + naltrexone-bupropion				
Grammer et al., 2023^62^ USA	30	Weight loss + CBT	Yes	EDE-Q	2 months	N/A
Mohseni et al., 2023^63^ the Netherlands	96	CBT + nutritional psychoeducation	No	EDE-Q, EDE-Q subscales eating, weight, and shape concern	15 months	N/A
da Luz et al., 2024^64^ Brazil	61	Nutrition education + CBT *(ED treatment)*	Yes	EDE-Q, EDE-Q subscales	3 months	9 months
Carbone et al., 2024^65^ Italy	22 (BED)	Naltrexone-bupropion + lifestyle modification *(ED treatment)*	Yes	EDE-Q, EDE-Q subscales	4 months	N/A
	20 (non-BED)		No			
Rahimi-Ardabili et al., 2024^44^ Australia	73	Behavioural weight loss + compassion therapy	No	EDE-Q, EDE-Q subscales	3 months	N/A
	44	Control (Nutrition information)				

Dallegrave et al.^38^ was not included at all in the meta-analysis.

Twenty-six arms from 20 studies included participants with a diagnosis of an eating disorder, 28 arms from 18 studies excluded people with eating disorders, 9 arms from 4 studies were unselective. When paired with weight loss interventions, psychological interventions either targeted the treatment of disordered eating,^30^, ^31^, ^32^, ^33^, ^34^, ^35^, ^36^ or aimed to enhance weight loss through body image therapy^37^^,^^38^ and self-compassion,^39^ for example. One study used cognitive behavioural therapy (CBT) to help participants maintain weight loss.^38^

Overall, 82% of participants were female, 57% had White ethnicity (when reported), the mean age ranged from 38 to 55 years, and the mean BMI ranged from 30.5 to 46.1 (when reported). Attrition ranged from 5% to 50% at the end of the intervention. The median duration of intervention was 4 months (range: 1–18 months) and 10 months (6–24 months) for the post intervention follow-up from baseline.

**Fig. 2:**
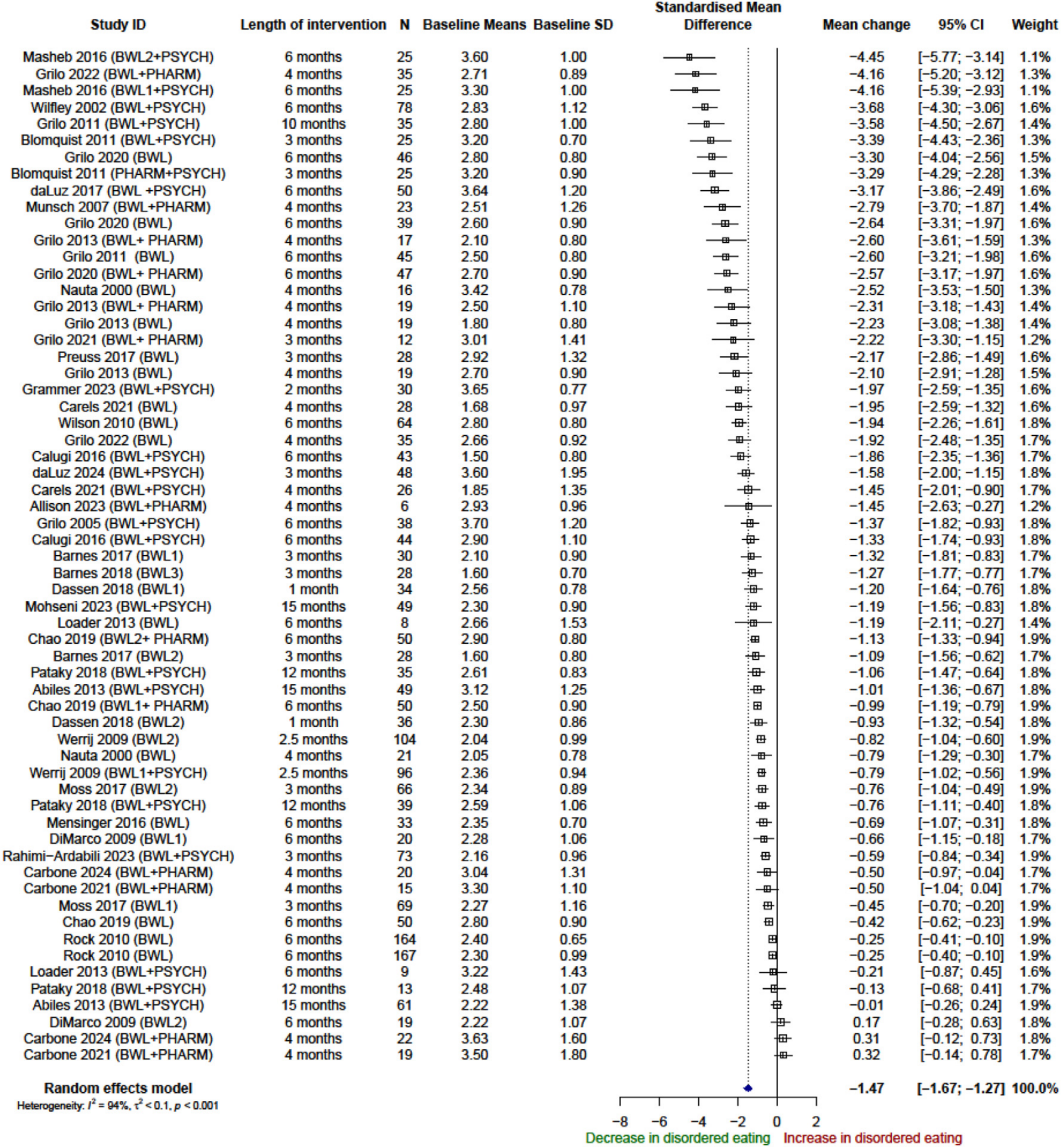
Association between weight loss interventions and disordered eating at the end of the intervention, pre-post analysis of single arms.

In the pre-post design, 61 eligible arms from 36 studies were included in the analysis of global EDE-Q/EDE scores. One study was not included at all in the meta-analysis; DalleGrave et al.^40^ did not report SD of the mean global scores. Ramirez et al.^37^ only reported two of the four subscale scores and no global scores, so it was only included in the relevant sub-scale meta-analyses. One study was only included in the pre-post analysis of longer-term follow-up, due to not reporting results at the end of the intervention duration stated.^38^ In the pre-post meta-analysis, there was clear and consistent evidence of a reduction in EDE-Q (−1.47 SMD; 95% CI, −1.67 to −1.27; I^2^ = 94%, n = 61) (Fig. 2) at the end of the intervention compared with baseline. This reduction was sustained at further follow-up (−1.54 SMD; 95% CI, −1.79 to −1.30; I^2^ = 93%, n = 34) compared with baseline. This was in the context of a mean weight change of −4.7 kg (95% CI, −5.73 to −3.7; I^2^ = 98%, n = 58) at the end of the intervention. Thereafter, there was mild weight regain with the mean weight change from baseline until further follow-up at −3.4 kg (95% CI, −4.91 to −1.85; I^2^ = 96%, n = 30) (Supplementary Materials, section A, Figure A3).

Across the EDE-Q/EDE sub-scales, there was some evidence of a significant increase in dietary restraint (Supplementary Materials, section A, Figure A6), whereas there was clear and consistent evidence of a significant reduction in all other subscales (weight concern, shape concern, eating concern) at the end of the intervention (Supplementary Materials, section A, Figures A7–A9). At further follow-up, there was no evidence that restraint differed from baseline (Supplementary Materials, section A, Figure A10), whereas there were still clear and consistent reductions in the other subscales (Supplementary Materials, section A, Figures A11–A13).

**Fig. 3:**
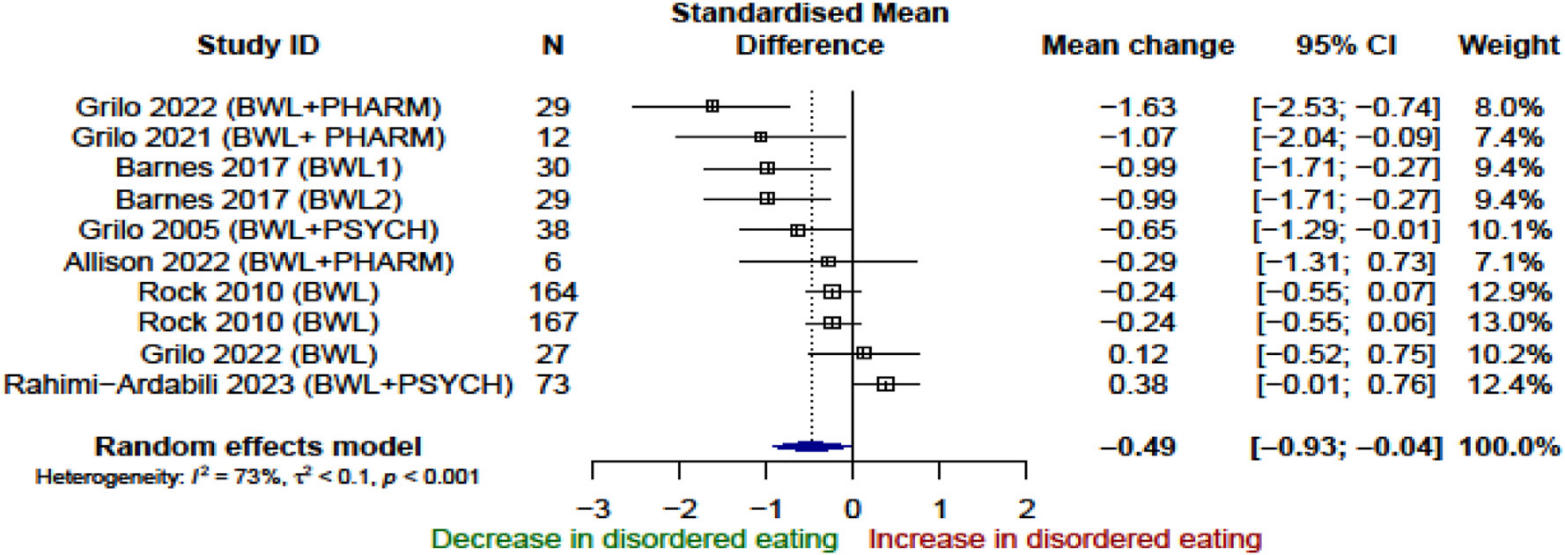
Association between weight loss interventions and disordered eating in the end of the intervention, analysis of randomised control trials.

Seven studies with 10 intervention arms were included in the intervention versus control meta-analysis.^34^^,^^41^, ^42^, ^43^, ^44^, ^45^, ^46^ Control groups involved placebo without dietary intervention,^41^^,^^45^^,^^46^ self-monitoring diary of behaviours without intervention,^34^ offering publicly available printed resources on diet and exercise,^42^ discouragement from starting a weight loss programme^43^ and standard nutrition information over email.^44^ The end of treatment effect of weight loss programmes versus minimal/no intervention was −0.49 SMD (95% CI, −0.93 to −0.04; I^2^ = 73%, n = 10) based on clear and consistent evidence (Fig. 3). Three studies followed up participants beyond intervention end and there was no evidence of an effect on disordered eating compared with control (0.10 SMD; 95% CI, −0.46 to 0.65; I^2^ = 0%, n = 3) (Supplementary Materials, section A, Figure A5).

**Fig. 4:**
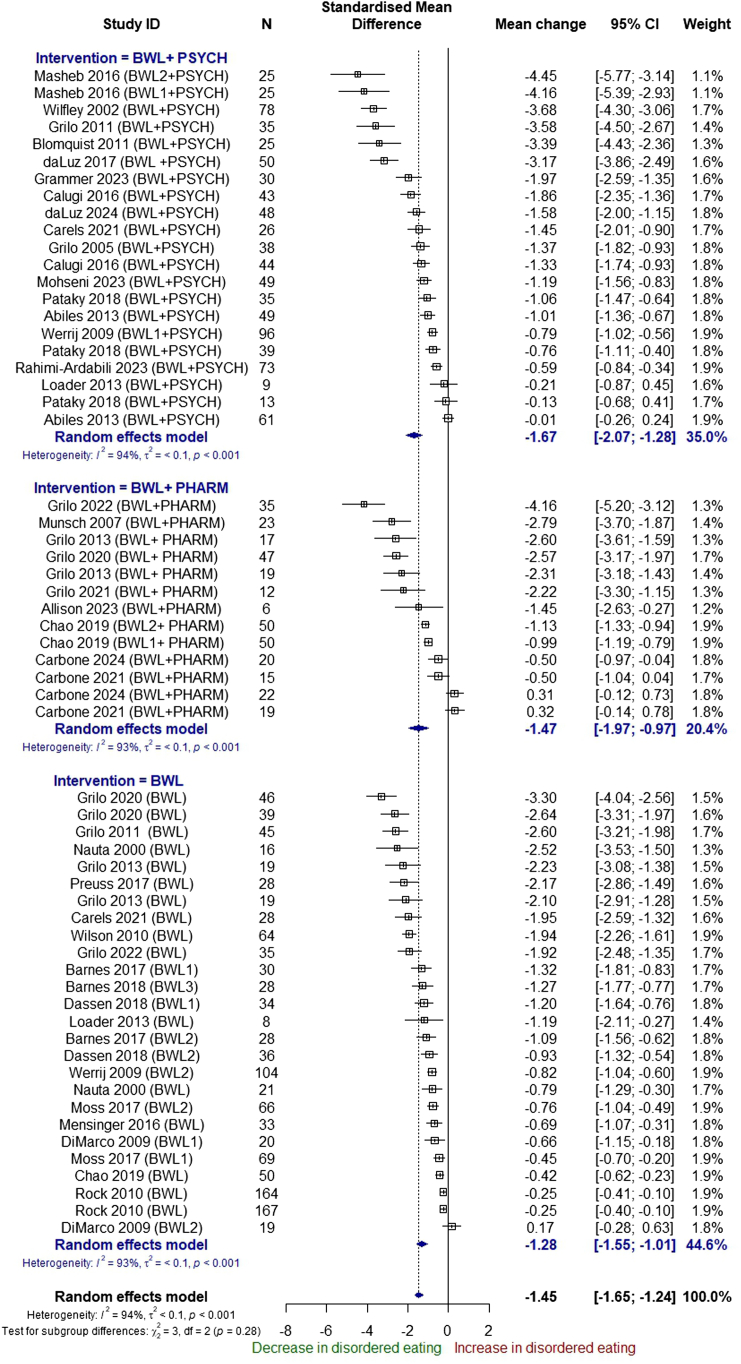
Subgroup analysis by type of intervention, pre-post analysis of singe arms.

In a subgroup analysis by type of intervention, excluding the one arm combining psychotherapy and pharmacotherapy, there was no evidence of subgroup differences (p = 0.28) between interventions that offered BWL alone, BWL and psychological therapy, and BWL and pharmacotherapy (Fig. 4).

**Fig. 5:**
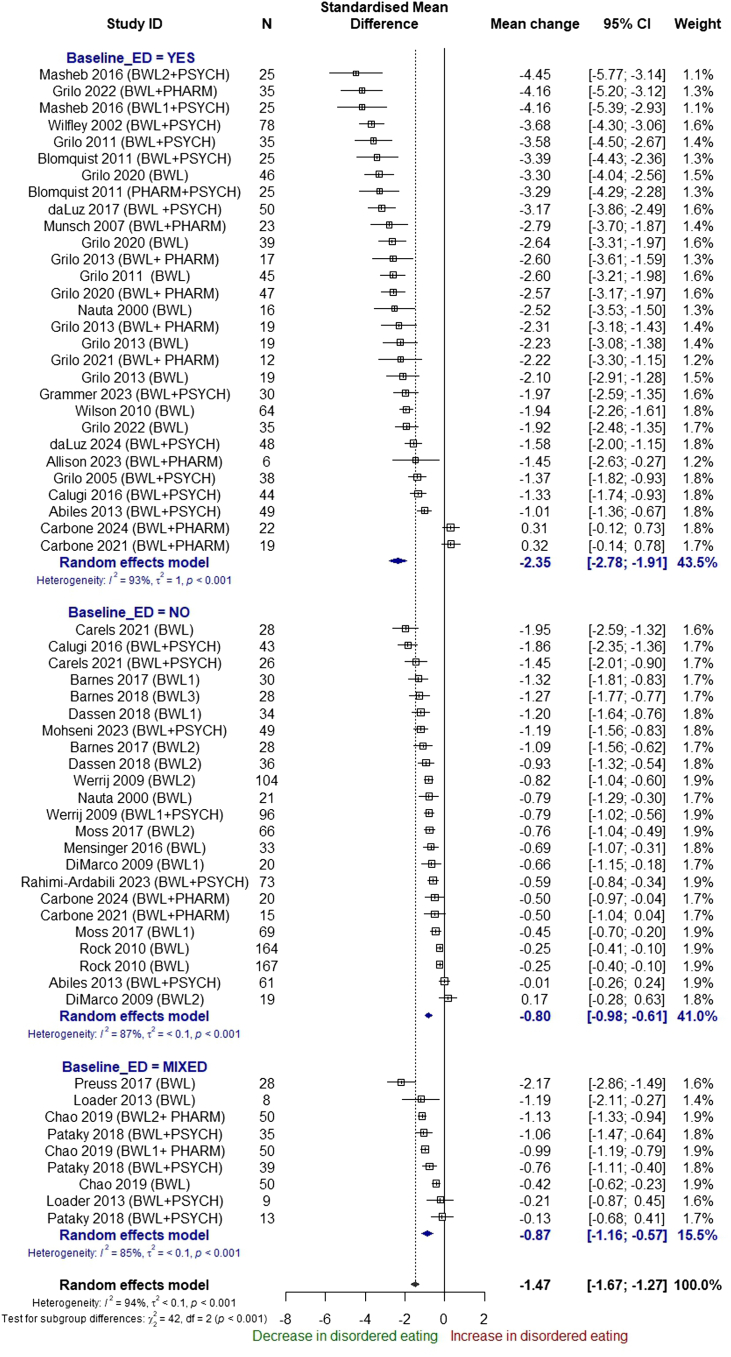
Subgroup analysis by baseline disordered eating status, pre-post analysis of single arms.

In a subgroup analysis by baseline disordered eating status, there was evidence of subgroup differences (p < 0.001). Participants diagnosed with an eating disorder at baseline saw the largest reduction in disordered eating scores (−2.35 SMD units; 95% CI −2.78 to −1.91; I^2^ = 93%, n = 29) compared with those without disordered eating (−0.80 SMD units; 95% CI, −0.98 to −0.61; I^2^ = 87%, n = 23) or the population unselected for disordered eating (−0.87 SMD units; 95% CI, −1.16 to −0.57; I^2^ = 85%, n = 9) (Fig. 5).

In the post-hoc subgroup analysis, there was no evidence of a difference between the studies reporting EDE-Q/EDE global scores directly (n = 48) versus studies where scores were calculated (p = 0.5) (n = 13).

In the meta-regression between change in disordered eating scores and weight change in the single-arm trials, weight loss was not associated with disordered eating change [regression coefficient −0.027 (95% CI −0.09 to 0.04)]. No other variables were included in the regression model. (Supplementary Materials, section A, Figure A16).

### Risk of bias

Seven studies were classified as at low, 20 as at medium, and 12 as at high risk of bias. In a sensitivity analysis, there was evidence of subgroup differences by risk of bias, with studies at low risk showing larger reductions in disordered eating scores compared with studies at either medium or high risk (Supplementary Materials, section A, Figure A4). There was no strong evidence of publication bias based on visual inspection of the funnel plot of studies using the EDE-Q and no statistically significant asymmetry indicated by Egger's test (p = 0.08). However, there was evidence of asymmetry in studies that assessed outcomes using the EDE, Egger's test (p = 0.02), (Supplementary Materials, section A, Figures A14 and A15).

## Discussion

In people with overweight/obesity, weight loss interventions producing modest weight loss were clearly and consistently associated with statistically significant improvement in disordered eating scores in analyses of pre-post single-arm and randomised controlled trials. The improvement in disordered eating was maintained at further follow-up and was independent of the type of intervention. The magnitude of weight loss did not predict changes in disordered eating. The improvement in disordered eating was larger in participants with pre-existing eating disorders than in participants without, or in participants unselected for disordered eating. Studies judged at low risk of bias showed larger effect sizes.

We followed the Cochrane guidance in conducting this review. To maximise data, we included both randomised and non-randomised trials, amounting to 38 trials with 3364 participants from 10 countries, making it, to our knowledge, the largest review in the field. It follows syntheses of evidence of weight loss interventions on disordered eating conducted in the past without a meta-analysis^12^^,^^47^ and extends the conclusions from a recent systematic review and meta-analysis^11^ which excluded interventions with pharmacotherapy and did not include trials among populations with eating disorders at baseline. By including more evidence and by calculating data needed to assess the change in EDE-Q/EDE score, the test statistic of most relevance, we produced more precise estimates than previous reviews.

The effect size estimates from the single-arm analyses were larger than the effect size observed in RCTs. One explanation for this is regression to the mean. Nearly half of the trials included participants selected to have an eating disorder or disordered eating at baseline and, on average, their scores are likely to decrease by follow-up. Trials are therefore likely to provide a better estimate of treatment effect. At treatment end, there was clear and consistent evidence of a benefit of weight loss treatment, but with only 2 RCTs (3 arms) the estimate at subsequent follow-up was too imprecise to draw conclusions on whether the effect was transient or persistent.

Another strength of this meta-analysis is the focus on studies that assessed disordered eating using validated tools, here EDE and EDE-Q. They are traditionally considered the “gold standard” to assess disordered eating psychopathology and demonstrate good agreement with each other and clinical diagnoses.^48^ There have been some concerns that the EDE-Q may overestimate binge eating frequency compared to EDE, which could inflate EDE-Q scores relative to the EDE. However, here we calculated change in score, which somewhat negates this concern and supports our decision to combine both measures together.

There were some weaknesses of our study and the data. There was high heterogeneity in weight loss outcomes and in eating disorder symptoms. We combined different types of weight loss interventions and subgroup analysis showed no evidence this reduced heterogeneity within these broad groups nor overall. The reduction in eating disorder symptoms was greatest in people with disordered eating at baseline. Despite this heterogeneity in effect size, the direction of effect was largely consistent. Another limitation was the small number of relevant randomised controlled trials, meaning evidence on causality is limited. In addition, 90% of the population was female, which is common in weight loss trials.^49^ Additionally, most studies reported a short follow-up, following participants for one year or less. Weight regain is common after programme completion^50^ and future trials could investigate how weight regain and participation in multiple weight loss attempts/programmes affect disordered eating in the long-term. Another limitation is that studies of people with eating disorders included mainly people with binge eating disorder (BED). BED is commonly comorbid with overweight/obesity,^51^^,^^52^ and weight loss programmes may not be considered appropriate initial treatment for people with bulimia or other eating disorders. We excluded all studies that did not use the EDE or EDE-Q. We confined our analysis to this measure because the EDE and EDE-Q are the most widely used quantitative assessments of the core symptoms of eating disorders in the field. However, we excluded 133 studies that had other measures that relate to eating disorders, such as measures of self-esteem, or offered a less comprehensive measure of eating disorders than that provided by EDE and EDE-Q. There is no reason to believe that these studies would have systematically different results to those we did include. We measured disordered eating using a continuous score not designed to diagnose eating disorders so we can say only that symptoms of eating disorders decline after enrolment in a weight loss programme. Finally, the median longest follow-up was 10 months. Consequently, this review cannot provide evidence on the development of eating disorders in the longer term.

Participants in these programmes saw a mean rise in the restraint subscale of the assessment tools. These tools were not designed to be administered to people undergoing BWL programmes, during which participants are encouraged to practice dietary restraint. As such, participants following the programme by restraining their intake were expected to increase their dietary restraint scores. However, that increase observed at the end of the intervention had dissipated by follow-up. Reassuringly, all other subscales (eating, shape and weight concern) significantly reduced both at the end of the intervention and at further follow-up, which led to the reduction in the global disordered eating score. This suggests that structured support to lose weight actually helps people address these challenges.

As new treatment options are being introduced to help people lose weight, there have been calls for implementation of screening for eating disorders before referral to weight loss interventions. However, screening adds significant cost to the healthcare system and time from referral to initiating the programme that may reduce access to these interventions. Our results provide reassurance that such screening is not essential. Further research on whether there are certain participant characteristics that could influence the outcome, apart from baseline disordered eating status, would be useful. There is a relevant individual participant data meta-analysis on the subject underway.^53^

In cognitive behavioural theory,^54^ eating disorders or disordered eating result from body dissatisfaction that prompts an individual to impose rigid rules to achieve a certain weight or shape. Efforts to adhere to extreme and highly specific rules can break down, for example at times of negative emotion, and temporary abandonment of the rules, perhaps leading to overeating and lowering self-esteem, leading to even more rigid rule formation. BWL programmes advise participants to impose structure and regular eating rather than extreme restraint rules. It is possible, therefore, that the support available and the flexibility of the programme rules may reduce the tendency for individuals to fall into this cycle of over-restraint and failure. Overeating is an established risk factor in the development of eating disorders, like BED,^55^ and compensatory weight control behaviours often precede the development of eating disorders,^56^ suggesting that potentially ineffective attempts to induce weight loss could increase disordered eating symptoms.

Despite the well-established benefits in physical health,^57^^,^^58^ weight loss attempts are often hypothesised to have a negative impact on mental health. Weight is often perceived as a sensitive topic, and doctors avoid raising the subject of weight loss due to its perceived negative consequences.^59^ Healthcare professionals have reported being hesitant to advocate for evidence-based effective treatments that involve weight loss, such as total diet replacement,^60^^,^^61^ which they report is to avoid the potential negative psychological impacts,^62^ with anecdotal concerns that these particularly restrictive dietary regimens could trigger eating disorders. There is also increasing concern in the public realm about “diet culture” and the impact of weight loss, especially the overvaluing of the thin ideal,^63^ on the risk of developing disordered eating.^64^ These concerns may relate to the harm that eating disorders do to quality of life^65^ and the co-occurrence of disordered eating and obesity.^51^^,^^52^

In summary, this review shows that BWL programmes, with or without weight loss pharmacotherapy or psychological therapy, do not worsen disordered eating in people with or without eating disorders and may even improve it.

## Contributors

ET was awarded funding to conduct this study. ET, DAK, RJP and PA designed the study. ET and GW/AT had access to and verified the underlying data used in this study. ET, DAK and PA developed the methodology and take responsibility for the accuracy of the data analysis. ET was responsible for the data administration and was supervised by DAK, RJP, PA, and SAJ. ET conducted the statistical analysis and drafted the manuscript. All authors critically reviewed or revised the manuscript and approved the final version of the manuscript.

## Data sharing statement

All data were publicly available. No additional data included to be made available.

## Declaration of interests

DAK, SAJ, and PA are investigators in two investigator-led publicly funded (NIHR) trials where the weight loss intervention was donated by Nestle Health Science and Oviva to the University of Oxford outside the submitted work. Second Nature is the commercial partner on GMW's MRC industrial collaborative awards in science and engineering (iCASE) studentship, but had no involvement in this review. None of these associations led to payments to these authors. DW received funding from the China Scholarship Council (CSC) for a visiting scholar position at the University of Oxford. This funding is unrelated to the content of the present manuscript. PA is an NIHR Senior Investigator and PA and SAJ are supported by the NIHR Oxford Biomedical WResearch Centre, the NIHR Oxford Health Biomedical Research Centre, and the NIHR Oxford and Thames Valley Applied Research Collaboration. DAK is supported by an NIHR Advanced Fellowship (NIHR302549). The funders had no role in the design and conduct of this work; collection, management, analysis, and interpretation of the data; preparation, review, or approval of the manuscript; and decision to submit the manuscript for publication. The views expressed are those of the author(s) and not necessarily those of the NIHR, or the Novo Nordisk UK Research Foundation.
