# Supplementary material for: The impact of weight loss interventions on disordered eating symptoms in people with overweight and obesity: a systematic review & meta-analysis
Source: eClinicalMedicine. 2025 Jan 31;80:103049. doi: 10.1016/j.eclinm.2024.103049 (PMC11841075; doi:10.1016/j.eclinm.2024.103049)
Supplement: Supplementary materials [file mmc1.docx]

### Supplementary materials

#### Appendix A- Figures and tables

Figure A1. Association between weight loss interventions and disordered eating in the further follow-up, pre-post analysis of single arms

Figure A2. Weight loss in the end of the intervention, pre-post analysis of single arm trials

Figure A3. Weight loss in the further follow-up, pre-post analysis of single arm trials

Figure A4. Sub-group analysis by risk of bias, pre-post analysis of single-arm trials

Figure A5. Association between weight loss interventions and disordered eating in the further follow-up, analysis of randomised control trials

Figure A6. Meta-analysis of restraint sub-scale in the end of the intervention, pre-post analysis of single arm trials****

Figure A7. Meta-analysis of eating concern sub-scale in the end of the intervention, pre-post analysis of single arm trials

Figure A8. Meta-analysis of weight concern sub-scale in the end of the intervention, pre-post analysis of single arm trials

Figure A9. Meta-analysis of shape concern sub-scale in the end of the intervention, pre-post analysis of single arm trials

Figure A10. Meta-analysis of restraint sub-scale in further follow-up, pre-post analysis of single arm trials

Figure A11. Meta-analysis of eating concern sub-scale in further follow-up, pre-post analysis of single arm trials

Figure A12. Meta-analysis of weight concern sub-scale in further follow-up, pre-post analysis of single arm trials

Figure A13. Meta-analysis of shape concern sub-scale in further follow-up, pre-post analysis of single arm trials

Table A1. Risk of bias table, RCTs

| **Study** | **Randomisation process** | **Effect pf assignment to intervention** | **Effect of adhering to intervention** | **Missing outcome data** | **Measurement of outcome bias** | **Selection bias** | **Total** |
| --- | --- | --- | --- | --- | --- | --- | --- |
| Berk et al., 2018  ^1^ |  |  |  |  |  |  |  |
| Grilo et al., 2005  ^2^ |  |  |  |  |  |  |  |
| Wilfley et al., 2002  ^3^ |  |  |  |  |  |  |  |
| Carels et al., 2021  ^4^ |  |  |  |  |  |  |  |
| Chao et al., 2019  ^5^ |  |  |  |  |  |  |  |
| da Luz et al., 2017  ^6^ |  |  |  |  |  |  |  |
| Mensinger et al., 2016  ^7^ |  |  |  |  |  |  |  |
| DiMarco et al., 2009  ^8^ |  |  |  |  |  |  |  |
| Grilo et al., 2021  ^9^ |  |  |  |  |  |  |  |
| Grilo et al., 2005  ^10^ |  |  |  |  |  |  |  |
| Dassen et al., 2018  ^11^ |  |  |  |  |  |  |  |
| Grilo et al., 2013  ^12^ |  |  |  |  |  |  |  |
| Grilo et al., 2020  ^13^ |  |  |  |  |  |  |  |
| Loader et al., 2013  ^14^ |  |  |  |  |  |  |  |
| Nauta et al., 2000  ^15^ |  |  |  |  |  |  |  |
| Grilo et al., 2011  ^16^ |  |  |  |  |  |  |  |
| Masheb et al., 2011  ^17^ |  |  |  |  |  |  |  |
| Munsch et al., 2007  ^18^ |  |  |  |  |  |  |  |
| Preuss et al. 2017  ^19^ |  |  |  |  |  |  |  |
| Ramirez et al., 2001  ^20^ |  |  |  |  |  |  |  |
| Rock et al., 2010  ^21^ |  |  |  |  |  |  |  |
| Barnes et al., 2017  ^22^ |  |  |  |  |  |  |  |
| Moss et al., 2017  ^23^ |  |  |  |  |  |  |  |
| Werrij et al., 2008  ^24^ |  |  |  |  |  |  |  |
| Wilson et al., 2010  ^25^ |  |  |  |  |  |  |  |
| Alisson et al., 2023  ^26^ |  |  |  |  |  |  |  |
| Grilo et al., 2022  ^27^ |  |  |  |  |  |  |  |
| Grammer et al., 2023^28^ |  |  |  |  |  |  |  |
| da Luz et al., 2024  ^29^ |  |  |  |  |  |  |  |
| Rahimi-Ardabili et al., 2024^30^ |  |  |  |  |  |  |  |

Table A2. Risk of bias table, observational studies

| **Study** | **Newcastle-Ottawa scale score** |
| --- | --- |
| Calugi et al., 2016  ^31^ |  |
| Carbone et al., 2021  ^32^ |  |
| DalleGrave et al., 2020  ^33^ |  |
| Abiles et al., 2013  ^34^ |  |
| Pataky et al., 2018  ^35^ |  |
| Barnes et al., 2018  ^36^ |  |
| Carbone et al., 2024  ^37^ |  |
| Mohseni et al., 2023  ^38^ |  |

Figure A14. Funnel plot of single arm trial publications at the end of the intervention (studies using EDE-Q)

Figure A15. Funnel plot of single arm trial publications at the end of the intervention (studies using EDE)

Figure A16. Meta-regression Bubble plot


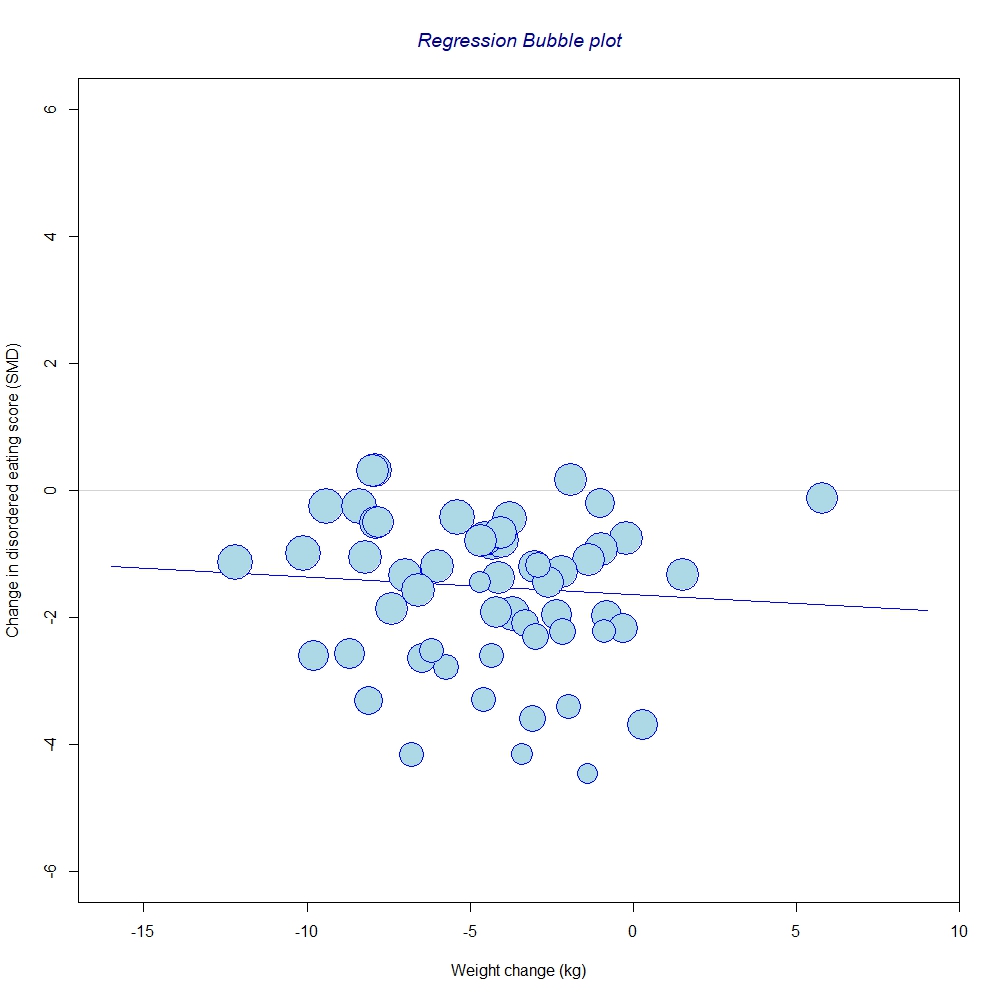


Appendix B. EMBASE search strategy (16.05.2022)

Embase 1974 to present

1 obesity/ or morbid obesity/ or abdominal obesity/ 510645

2 (overweight or over weight or overeat* or over eat* or overfeed* or over feed*).ti,ab. 126953

3 obes*.ti,ab. 507081

4 1 or 2 or 3 659127

5 weight loss program/ 2875

6 body weight loss/ and (modific* or therap* or intervention* or strateg* or program* or management or scheme* or group* or club? or pathway* or service?).ti,ab. 38268

7 ((weight adj1 (los* or reduc* or manag*)) and (modific* or therap* or intervention* or strateg* or program* or management or scheme* or group* or club? or pathway* or service?)).ti,ab. 119546

8 obesity/dt, pc, th or morbid obesity/dt, pc, th or abdominal obesity/dt, pc, th 44994

9 diet restriction/ or caloric restriction/ or diet therapy/ or exp low calorie diet/ or low fat diet/ or high-protein low-carbohydrate diet/ 175521

10 (diets or dieting).ti,ab. 123863

11 ((low calorie* or low energy or hypocaloric or calorie control* or low fat* or fat control* or low carbohydrate* or carbohydrate control*) adj2 diet*).ti,ab. 16509

12 (health* adj1 eating).ti,ab. 13219

13 (diet* adj2 (modific* or therap* or intervention* or strateg* or program* or management or scheme* or group* or club?)).ti,ab. 66032

14 (nutrition* adj2 (modific* or therap* or intervention* or strateg* or program* or management or scheme*)).ti,ab. 38211

15 (Weight Watchers or slimming world or lighterlife or lighter life or jenny craig or diet chef or nutrisystem or slimfast or slim fast or medifast or optifast or modifast or nutrilett or hmr or cambridge diet).ti,ab. 2458

16 (commercial* adj2 (program* or intervention? or weight loss or diet?)).ti,ab. 3551

17 (meal? adj2 replace*).ti,ab. 1224

18 ((preprepared or pre-prepared or prepared) adj2 (meal? or food? or snack? or portion?)).ti,ab. 2339

19 ((prepack* or pre-pack*) adj2 (meal? or food? or snack? or portion?)).ti,ab. 303

20 (eating plan? or meal plan* or planned meal? or planned menu?).ti,ab. 1364

21 (portion control* adj2 (diet? or meal? or food? or weight loss)).ti,ab. 108

22 (liquid adj2 (diet? or meal? or food?)).ti,ab. 11074

23 (replace* adj (snack? or drink? or food? or liquid?)).ti,ab. 139

24 shake?.ti,ab. 16202

25 (isoenergetic adj (diet? or food? or meal?)).ti,ab. 605

26 cognitive behavioral therapy/ or behavior therapy/ or cognitive therapy/ or counseling/ 167302

27 (((behavio* or cognitive) adj2 therap*) or cbt).ti,ab. 53453

28 ((chang* or modif*) adj2 (lifestyle* or behavio?r)).ti,ab. 77524

29 (orlistat or xenical).tw,ot. 3819

30 ("Ro 18 0647" or Ro 18-0647 or Ro 180647 or Ro18647).tw,ot. 7

31 (sibutramin$ or arcalion).tw,ot. 1764

32 (Bts 54 524 or Bts 54524 or Bts54524).tw,ot. 20

33 (reductil or medaria or meridia).tw,ot. 748

34 (rimonabant or acomplia or zimulti).tw,ot. 2427

35 (Sr 141716 or Sr141716 or Sr 141716a or Sr141716a).tw,ot. 3048

36 ((eating adj2 disorder*) or bulimi* or purg*).ti,ab. 44500

37 (selfimage or self image or bodyimage or body image).mp. 32765

38 (Eating adj2 Disorder*).mp. 43893

39 or/36-38 83480

40 or/5-35 771626

41 4 and 39 and 40 5072

42 randomized controlled trial/ 708056

43 single blind procedure/ or double blind procedure/ 238586

44 crossover procedure/ 70276

45 random*.tw. 1786455

46 (((singl* or doubl*) adj (blind* or mask*)) or crossover or cross over or factorial* or latin square or assign* or allocat* or volunteer*).ti,ab. 1196596

47 or/42-46 2600059

48 41 and 47 824

49 (exp animals/ or nonhuman/) not human/ 6952191

50 48 not 49 **816**

#### Appendix C. Full characteristics of included studies

| **Study and country** | **Intervention in detail** | **Intervention in brief** | **Population** | **Provider** | **Duration** | **Long term follow-up** | **Total or partial meal replacement** | **Eligible comparison** | **Geographical area** | **Age,**  **mean years (±SD)** | **Participants,% female** | **Ethnicity, % White** |
| --- | --- | --- | --- | --- | --- | --- | --- | --- | --- | --- | --- | --- |
| Berk et al., 2018,  the Netherlands  ^1^ | ***VLCD+ group CBT***: Starts with a diet very low in energy (very low calorie diet; VLCD) of approximately 750 kcal/day for 8 weeks. Blocks of 20 participants started with the diet concomitantly. The daily diet consisted of two diabetes-specific meal replacements plus 75 g lean meat, 150 ml skimmed milk and low-carbohydrate vegetables ad libitum. After 8 weeks the diet was changed into a low energy diet of 1100–1300 kcal/day, gradually increasing the intake during the following 12 weeks. From then on, the participants ate a diet based on national health recommendations, aiming at weight maintenance. In the end of the diet, participants started group-CBT with up to ten participants per group. The first ten weekly sessions were followed by two fortnightly sessions, two monthly sessions and two 3monthly sessions. | Very low calorie diet, re-introduction of food and then cognitive behavioural therapy | Overweight and obese (BMI >27 kg/m2) adults with type 2 diabetes and aged 18–75 | Trained psychologist/psychotherapist, dietitian, diabetes nurse | 18m (higher intensity up till ~6 months) | 2 years | YES | NO | Europe & the UK | 52.3 ± 11.3 | 53% | 50.6% |
|  | ***VLCD***: Starts with a diet very low in energy (very low calorie diet; VLCD) of approximately 750 kcal/day for 8 weeks. Blocks of 20 participants started with the diet concomitantly. The daily diet consisted of two diabetes-specific meal replacements plus 75 g lean meat, 150 ml skimmed milk and low-carbohydrate vegetables ad libitum. After 8 weeks the diet was changed into a low energy diet of 1100–1300 kcal/day, gradually increasing the intake during the following 12 weeks. From then on, scheduled visits every 3–6 months (sometimes on separate occasions) to the internist and diabetes nurse, plus referral to a dietitian or psychologist when indicated. | Very low calorie diet and then re-introduction of food |  | Dietitian, diabetes nurse ±trained psychologist/psychotherapist | ~6 months | 2 years | YES | NO |  | 55.2 ± 9.3 | 58.7% | 60% |
| Grilo et al., 2005  ^2^ | ***Diet + Pharmacotherapy + CBT self-help***: Treatment was administered in double-blind placebo-controlled fashion. Participants received a fixed dose of 120 mg orlistat 3 times/day with meals during the 12-week study along with basic medication management procedures. Participants were instructed to adhere to the following guidelines: 1) eat three meals and two to three snacks per day; 2) aim for modest balanced calorie diet with goals of 1200 kcal/day for women and 1500 kcal/day for men; 3) limit fat to less than 30% of intake; and 4) follow United States Department of Agriculture  (USDA) Food Guide Pyramid to aid in balanced food choices and portion sizes. The CBT was administered individually using a guided-self-help approach. Participants were given a copy of Overcoming Binge Eating, 25 a step-by-step self-help version of the therapist manual. The guided-self-help protocol included six brief (15–20 min) individual meetings with doctoral research-clinicians during the 12-week study. The focus was primarily on (a) maintaining and enhancing motivation; (b) correcting misunderstanding of the information; (c) addressing difficulties with relevant skill-building exercises; and (d) reinforcing the importance of self-monitoring and record keeping. | Moderate energy restriction food based weight loss, weight loss medication (orlistat) and cognitive behavioural therapy self-help guidance | Adults between the age 35-60, living with obesity and meeting full DSM-IC criteria for binge eating disorder | Doctors | 3 months | 6 months | NO | NO | North America | 45.2 ± 7.4 | 84% | 88% |
|  | ***Diet + placebo + CBT self-help***:  Along with a placebo tablet 3 times/day, following guidelines: 1) eat three meals and two to three snacks per day; 2) aim for modest balanced calorie diet with goals of 1200 kcal/day for women and 1500 kcal/day for men; 3) limit fat to less than 30% of intake; and 4) follow United States Department of Agriculture (USDA) Food Guide Pyramid to aid in balanced food choices and portion sizes. The CBT was administered individually using a guided-self-help approach. Participants were given a copy of Overcoming Binge Eating, 25 a step-by-step selfhelp version of the therapist manual.9 The guided-selfhelp protocol included six brief (15–20 min) individual meetings with doctoral research-clinicians during the 12-week study. The clinicians focused primarily on (a) maintaining and enhancing motivation; (b) correcting misunderstanding of the information; (c) addressing difficulties with relevant skill-building exercises; and (d) reinforcing the importance of self-monitoring and record keeping. | Moderate energy restriction food based weight loss, placebo and cognitive behavioural therapy self-help guidance |  | Doctors | 3 months | 6 months | NO | NO |  | 47 ± 7 | 92% | 88% |
| Wilfley et al., 2002  ^3^ | ***Individualised weight loss+ Group CBT for BED***:  In the first phase of the CBT phase (sessions 1-6), behavioural strategies (eg, self-monitoring) were used to help patients identify episodes of over-restriction and under-restriction and encouraged normalising of eating patterns. During the second phase (sessions 7-14), patients learned cognitive skills to counter negative thoughts identified as predisposing binge eating. Cognitive restructuring helps patients challenge harsh stereotyped views of overweight and promotes acceptance of diverse body sizes. In the third phase (sessions 15-20), relapse prevention techniques, such as problem solving and coping with high-risk situations, are presented to help with maintaining changes. Patients are encouraged to identify reasonable goals and strategies for weight loss that will not promote binge eating. | Cognitive behavioural therapy in a group setting, with setting personalised goals for weight loss | Adults aged 18-65 years, living with overweight/obesity (27-48 kg/m^2^) and meeting DSM-/V research criteria for binge-eating disorder | Therapists | 6 months | 10 months | NO | NO | North America | 45.6 ± 9.6 | 82.7% | 93.9% |
| Calugi et al., 2016  ^31^ | ***Inpatient weight loss + CBT***:  The residential program lasted 21 days, and included the following procedures: (i) a low calorie diet (1,200 kcal/day, 50% of which from carbohydrates, 15% from proteins, and 30% from fat); (ii) 30 min per day of indoor cycling and two 45-min sessions per week of calisthenics and (iii) daily group CBT sessions. The group CBT sessions included the following strategies and procedures: (i) education on energy balance, the food pyramid, portion size, regular eating, calorie counting, shopping and food labels, physical activity (what, when, and how much); (ii) self-monitoring of food intake, energy and body weight; (iii) stimulus control strategies (in particular how to at home); (iv) problem solving; (v) cognitive restructuring of dysfunctional thoughts that hinder weight loss and weight-loss maintenance; (vi) relapse prevention; (vii) involving significant others to create an environment that facilitates adherence to lifestyle modification aimed at weight loss. The behavioural component of the treatment was based on the principles of the LEARN program for weight control^39^ and the cognitive component on the CBT for obesity developed by Cooper et al.^40^ | Moderate energy restriction inpatient weight loss programme (based on LEARN) with cognitive behavioural therapy | Females aged 18-65, referred for inpatient obesity treatment  Females aged 18-65 with binge eating disorder, referred for inpatient obesity treatment | Hospital staff | ~1 month intensive and then as outpatient (in total 6 months) | X | NO | NO | Europe + the UK | 41.6 ± 13.4  38.8 ± 13.7 | 100% | Not reported |
| Carbone et al., 2021  ^32^ | ***Pharmacotherapy + diet:*** Participants started with 1x tablet a day of naltrexone-bupropion available as prolonged release (containing 8 mg of naltrexone·HCl and 90 mg of bupropion·HCl) and then slowly increased to a maximum of 2 tablets twice a day after the third week. Therapy lasted for another 13 weeks, making a total of 16 weeks. Participants were allowed not to receive the maximum dose in cases of bearable side effects (e.g., nausea, constipation). Alongside the pharmacotherapy, participants were prescribed a hypo-caloric diet (reducing daily calorie intake of about 500 kcal), behavioural counselling and moderate aerobic physical activity (i.e., 20-min walk every day) during the 16 weeks. | Moderate energy restriction weight loss programme paired with weight-loss medication (naltrexone-bupropion) | Adults aged 18-65, meeting DSM-5 criteria for binge eating disorder diagnosis and obesity (BMI ≥30 kg/m^2^)  Adults aged 18-65, living with obesity (BMI ≥30 kg/m^2^) | Medical staff | 4 months | X | NO | NO | Europe + the UK | 41 ± 13.2  44.4 ± 14 | 79%  53% | Not reported |
| Carels et al., 2021  ^4^ | ***Behavioural weight loss programme + psychological intervention (self-compassion skills training)***: The BWLP was an abridged adaptation of a 12- week of the Diabetes Prevention Programme^41^. Chapters emphasized calorie and macronutrient tracking, increased exercise, self-monitoring, general tips for reducing caloric and fat intake and behaviour-focused interventions (e.g., learning cues and triggers for overeating). Participants were offered a workbook and the focus each week was on one chapter during group sessions. Participants were encouraged to monitor their energy intake using the MyFitnessPal application, a free calorie counter (MyFitnessPal.com or a smartphone application version). Weekly groups were facilitated by graduate trainees in a Clinical Psychology Doctoral programme, supervised by a licensed psychologist. Each group had between 5 and 15 participants. The BWLP intervention was supplemented with brief self-compassion skills training. Self-compassion was framed as a supportive tool to nurture adaptive cognitive and behavioural changes throughout the weight loss program, and included the following topics: self compassionate eating, self-appreciation, evaluation of critical self-talk, increasing SC through writing, journaling, and self-care. Training consisted of psychoeducation and associated discussion regarding the application of self-compassion to support physical and psychological health. | Moderate energy restriction weight loss programme (based on DPP), paired with self-compassion skills training | Adults aged 18 or older, BMI≥27 kg/m^2^ | Graduate trainees in a Clinical Psychology Doctoral programme, supervised by a licensed psychologist | 3 months | X | NO | NO | Europe + the UK | 52 ± 12.6 | 76.9% | 42.3% |
|  | ***Behavioural weight loss programme alone:*** The BWLP was an abridged adaptation of a 12- week of the Diabetes Prevention Programme^42^. Chapters emphasized calorie and macronutrient tracking, increased exercise, self-monitoring, general tips for reducing caloric and fat intake and behaviour-focused interventions (e.g., learning cues and triggers for overeating). Participants were offered a workbook and the focus each week was on one chapter during group sessions. Participants were encouraged to monitor their energy intake using the MyFitnessPal application, a free calorie counter (MyFitnessPal.com or a smartphone application version). Weekly groups were facilitated by graduate trainees in a Clinical Psychology Doctoral programme, supervised by a licensed psychologist. Each group had between 5 and 15 participants. | Moderate energy restriction weight loss programme (based on DPP) | Adults aged 18 or older, BMI≥27 kg/m^2^ | Graduate trainees in a Clinical Psychology Doctoral programme, supervised by a licensed psychologist | 3 months | X | NO | NO |  | 52.4 ± 12.1 | 85.7% | 64.3% |
| Chao et al., 2019  ^5^ | ***Intensive Behavioural therapy only***:  The programme consisted of 21 sessions of IBT, delivered by a physician, nurse practitioner, or registered dietitian. Sessions were structured, lasted 15 minutes, and were delivered as 4 weekly visits, then 10 every-other-week sessions, followed by 7 visits every 4 weeks. Participants with a weight of <113.6 kg were prescribed a diet of 1,200 to 1,499 kcal/d of conventional foods, and those with a weight of ≥113.6 kg were prescribed 1,500 to 1,800 kcal/d. Approximately 15% to 20% of kilocalories were from protein, 20% to 35% from fat, and the remainder from carbohydrates. Participants were instructed to record their food and calorie intake daily. They were encouraged to engage in low- to moderate-intensity physical activity 5 days per week, gradually building to ≥225 min/week from weeks 25 to 52. The CMS‐based IBT program followed an abbreviated lifestyle counselling protocol adapted from the Diabetes Prevention Program DPP^42^ for delivery in primary care settings. | Mild energy restriction weight loss programme (based on DPP) | Adults aged 21 to 70 years, BMI 30-55 kg/m^2^ | Physician, nurse practitioner, or registered dietitian | 6 months | 12 months | NO | NO | North America | 49.5 ± 11 | 78% | 54% |
|  | ***Intensive Behavioural therapy + liraglutide:***  The programme consisted of 21 sessions of IBT, delivered by a physician, nurse practitioner, or registered dietitian. Sessions were structured, lasted 15 minutes, and were delivered as 4 weekly visits, then 10 every-other-week sessions, followed by 7 visits every 4 weeks. Participants with a weight of <113.6 kg were prescribed a diet of 1,200 to 1,499 kcal/d of conventional foods, and those with a weight of ≥113.6 kg were prescribed 1,500 to 1,800 kcal/d. Approximately 15% to 20% of kilocalories were from protein, 20% to 35% from fat, and the remainder from carbohydrates. Participants were instructed to record their food and calorie intake daily. They were encouraged to engage in low- to moderate-intensity physical activity 5 days per week, gradually building to ≥225 min/week from weeks 25 to 52. The CMS‐based IBT program followed an abbreviated lifestyle counselling protocol adapted from the Diabetes Prevention Program DPP^42^ for delivery in primary care settings. Participants were also prescribed liraglutide 3.0 mg/day as an once-daily, self-administered, subcutaneous injection. As recommended, the medication was initiated at 0.6 mg/d for 1 week and increased by 0.6 mg/d in weekly intervals until 3.0 mg/d was achieved. | Mild energy restriction weight loss programme (based on DPP) + medication (liraglutide) | Adults aged 21 to 70 years, BMI 30-55 kg/m^2^ | Physician, nurse practitioner, or registered dietitian |  |  | NO | NO |  | 45.2 ± 12.3 | 84% |  |
|  | ***Intensive Behavioural therapy with 12-week total diet replacement+ liraglutide*** :  The programme consisted of 21 sessions of IBT, delivered by a physician, nurse practitioner, or registered dietitian. Sessions were structured, lasted 15 minutes, and were delivered as 4 weekly visits, then 10 every-other-week sessions, followed by 7 visits every 4 weeks. Participants with a weight of <113.6 kg were prescribed a diet of 1,200 to 1,499 kcal/d of conventional foods, and those with a weight of ≥113.6 kg were prescribed 1,500 to 1,800 kcal/d. Approximately 15% to 20% of kilocalories were from protein, 20% to 35% from fat, and the remainder from carbohydrates. Participants were instructed to record their food and calorie intake daily. They were encouraged to engage in low- to moderate-intensity physical activity 5 days per week, gradually building to ≥225 min/week from weeks 25 to 52. The CMS‐based IBT program followed an abbreviated lifestyle counselling protocol adapted from the Diabetes Prevention Program DPP^42^ for delivery in primary care settings. Participants were prescribed liraglutide 3.0 mg/d as a once-daily, self-administered, subcutaneous injection. As recommended, the medication was initiated at 0.6 mg/d for 1 week and increased by 0.6 mg/d in weekly intervals until 3.0 mg/d was achieved. These participants received the same treatment as those in IBT-liraglutide except that, at week 4, they were prescribed a 12-week, 1,000- to 1,200-kcal/d diet that provided four servings daily of a liquid shake (Health Management Resources, Boston, Massachusetts; 160 kcal per shake) and an evening meal of a frozen food entrée (250-300 kcal), with a serving of fruit and salad. | Mild energy restriction weight loss programme (based on DPP) followed by a 12-week total diet replacement regimen, paired with medication (liraglutide) | Adults aged 21 to 70 years, BMI 30-55 kg/m^2^ | Physician, nurse practitioner, or registered dietitian |  |  | YES | NO |  | 48 ± 11.9 | 76% |  |
| da Luz et al., 2017  ^6^ | ***Subtle dieting + psychological support***:  HAPIFED is a manualized program composed of five stages^6^. Stage one (sessions 2–11) mainly offers psychoeducation about ED symptoms and behaviours and weight, in relation to mental and physical risks. Real-time self-monitoring is introduced and encouraged, with the additional evaluation of internal cues related to hunger and satisfaction. Stage two (session 12): the personal formulation process is revised and improved. A joint review of progress/identifying barriers to change is conducted and each participant had the opportunity to discuss/share/reflect on her/his own formulation. In other sessions, when issues emerged for the participant relevant to the formulation, the therapists explored these, linking theory to practice, with reference to the situation referred by the participant. Stage three (sessions 13–19): the focus is on behavioural change and monitoring through the practice of specific behavioural skills, e.g. activities that emphasize the identification of body’s positive aspects, tasks that promote better self-care, and skills training in progressive muscular behavioural relaxation, etc. Stage four (sessions 20–27) highlights the relevance of changing unhealthy beliefs and attitudes for modification of unsuitable ED behaviours. Stage five (sessions 28–30) includes management of relapses and recheck of healthy cognitive and behavioural strategies. Features unique to HAPIFED include psychoeducation around ED and high weight, the monitoring of internal cues of hunger and satiety, dietitian-led nutritional counselling, and weight loss strategies including increased physical activity. Also, two home visits by an occupational therapist for evaluation of the domestic environment and the person’s daily routines, and advice on improvements for food preparation and physical activities (in stages 1 and 3) were included. | Mild energy restriction weight loss programme with psychoeducation on disordered eating and | Adults with BMI 27-40 kgm^2^ and presence of recurrent binge eating with diagnosis of BN, BED, or OSFED/UFED according to the DSM-5 criteria | Therapists and nutritionists/dietitians | 6 months | 12 months | NO | NO | South America | 40.55 ± 11.7 | 96% | 75% |
| DalleGrave et al., 2020  ^33^ | ***Weight loss + psychotherapy***:  CBT-OB associates specific physical activity (i.e. reducing sedentary activities and gradually increasing the number of daily steps until the goal of 10,000–12,000 steps per day is reached) and dietary recommendations strategies (i.e. a meal plan based on Mediterranean diet to produce a 500- kcal energy deficit per day with cognitive behavioural procedures. Except for the first two sessions, in which the therapist describes the use of meal planning, self-monitoring in real time, and the strategies for developing an active lifestyle, sessions are dedicated to discussing how to address the weight-loss obstacles that each participant has identified in their Weight-Loss Obstacles Questionnaire—a self-report questionnaire that includes key questions investigating whether or not they are applying the treatment procedures, and whether any other behaviours or attitudes that might hinder weight loss have occurred over the previous week. By encouraging patients to change the way that they behave and analyse the effects and implications of such behavioural modifications, CBT-OB promotes cognitive change (i.e. reduction of unrealistic weight-loss expectations, improvement in satisfaction with progress achieved during treatment, improvement in dietary restraint, reduction in disinhibition, improvement in satisfaction with the results achieved), training patients to manipulate not only their behaviour, but also their frame of mind. By being encouraged to try different ways of behaving and assessing the effects and implications thereof, patients are given an opportunity to develop a healthier approach to eating. Once new, healthier eating and physical activity habits have become the new norm, and patients have developed a persistent weight-control mind-set, they are helped to identify stimuli that are likely to “reinstall” their unhealthy weight-gain mind-set; they are taught to recognize the signs that this is occurring, and to take the appropriate action immediately (generally doing the opposite of the behaviour driven by the weight-gain mind-set). These skills are designed to help patients to deal effectively and efficiently with setbacks that might otherwise develop into a full-scale relapse. | Moderate energy restriction weight loss programme (based on the Mediterranean diet), paired with cognitive behavioural therapy for obesity | Adults aged 20-65 with a BMI ≥30 kg/m^2^, without severe psychiatric disorders like bulimia nervosa | 3 physicians, two of whom specialise in clinical nutrition and one in nursing | 18 months | N/A | NO | NO | Europe and the UK | 45.8 ± 11.1 | 76% | Not reported |
| Mensinger et al., 2016  ^7^ | ***Weight loss programme***:  Participants received the LEARN Program for Weight Management, which stands for Lifestyle, Exercise, Attitudes, Relationships, and Nutrition^39^. This evidence-based behaviour modification programme emphasizes weight loss as an ultimate goal of the program, while focusing on gaining skills to overcome weight loss barriers, and learning how to change diet and lifestyle. Participants received the 10th edition of the LEARN Program for Weight Management manual and the LEARN Weight Stabilization and Maintenance Guide along with the LEARN Program CD set. In addition to maintaining food diaries and physical activity logs between the scheduled program meetings each week, participants were expected to complete exercises from the manual. Examples of the exercises included: (a) a self-assessment of eating risk factors, (b) a worksheet to prepare one with coping skills for “high risk situations” that might lead to overeating, (c) an eating habits checklist, (d) a nutrition quiz, and (e) an exercise quiz. Participants were also encouraged to maintain their lifestyle changes by utilizing the social support network developed during the program. Email and phone number lists were created and distributed to help facilitate this network. The programme emphasized the importance of healthy lifestyle choices and gradual sustainable change. However, the conventional weight management program made weight loss an explicit goal and focused on food intake levels based on external prescriptions and caloric restriction. | Mild energy restriction weight loss programme (based on LEARN) with exercise | Females aged 30-45 and BMI between 30 and 45 kg/m^2^ | Registered dietitian with over 15 years of experience working with bariatric populations and patients with type 2 diabetes within individual and group settings | 6 months | 2 years | NO | NO | North America | 39.35 ± 3.9 | 100% | 95% |
| DiMarco et al., 2009  ^8^ | ***Standard guided self-help weight loss + motivational interviewing:***  The skill-building lesson plans include goal setting, environmental restructuring, logging, social support, walking, positive attitude and self-talk, body image, cravings, stress, relapse prevention, “plateau busting,” and confidence building. The widely used LEARN programme^39^ encourages gradually losing weight, progressively increasing physical activity, and decreasing energy and fat intake through permanent lifestyle changes. The program emphasizes: (a) self-monitoring of eating behaviour; (b) controlling stimuli associated with eating; (c) physical activity; (d) nutrition education; (e) modifying self-defeating thoughts and negative emotions associated with dieting and body image; (f) setting realistic goals; (g) relationships; and (h) relapse prevention and weight maintenance. The nutritional guidance is consistent with federal guidelines. In this programme, these sessions incorporated motivational interviewing techniques, such as a decisional balance exercise, aimed at encouraging participants to explore ambivalence about making changes. | Guided self-help mild energy restriction weight loss programme (based on LEARN), paired with motivational interviewing | Adults aged 18 to 55, BMI between 27 and 40 kg/m^2^ | Therapists | 3 months | X | NO | NO | North America | 39.9 ± 8.84 | 82% | 71.8% |
|  | ***Standard guided self-help weight loss:***  The skill-building lesson plans include goal setting, environmental restructuring, logging, social support, walking, positive attitude and self-talk, body image, cravings, stress, relapse prevention, “plateau busting,” and confidence building. The widely used LEARN programme^39^ encourages gradually losing weight, progressively increasing physical activity, and decreasing energy and fat intake through permanent lifestyle changes. The program emphasizes: (a) self-monitoring of eating behaviour; (b) controlling stimuli associated with eating; (c) physical activity; (d) nutrition education; (e) modifying self-defeating thoughts and negative emotions associated with dieting and body image; (f) setting realistic goals; (g) relationships; and (h) relapse prevention and weight maintenance. The nutritional guidance is consistent with federal guidelines. | Guided self-help mild energy restriction weight loss programme (based on LEARN) |  | Therapists | 3 months | X | NO | NO | North America |  |  |  |
| Grilo et al., 2021  ^9^ | ***Pharmacotherapy + mild weight loss advice:***  Medication offered in this intervention were naltrexone and buproprion. The up-titration schedule as follows: Study day 1, placebo lead-in (both groups); study days 2 and 3, 150 mg of bupropion (active-treatment group) ; study days 4 and 5, 300 mg of bupropion (active-treatment group); study days 6 through 84 (end), 50 mg of naltrexone and 300 mg of bupropion (active treatment group). At the end of 12 weeks, participants followed a down-titration protocol of 7 days of 150 mg of bupropion (active-treatment group). | Mild energy restriction weight loss programme with weight loss medication (naltrexone and buproprion) | Adults aged 18-65, meeting DSM-5 criteria for binge-eating disorder and a BMI 30-50 kg/m^2^ | Not specified | 3 months | 6 months | NO | YES- placebo control, minimal advice | North America | 51.17 ± 8. | 92% | 75% |
| Grilo et al., 2005  ^10^ | ***Behavioural weight loss—guided self-help (BWLgsh):***  The intervention uses the LEARN Program for Weight Management. This programme is widely used by both participants and professionals and is frequently the manual of choice in university-based clinics, and has received empirical support for its use as a self-help method. LEARN is an acronym for lifestyle, exercise, attitudes, relationships, and nutrition. LEARN focuses on making gradual and moderate lifestyle changes with goals of moderate caloric restriction and increased physical activity to produce modest gradual weight losses. Participants were provided with a copy of the self-help book^39^, which is comprised of 16 lessons covering various aspects of weight loss. The nutritional guidance is consistent with federal guidelines. The book is structured with a series of steps that address how to assess and change eating and activity behaviours. The steps are concretely described and are presented in an additive fashion yet with redundancy to facilitate mastery. The program emphasizes: (a) self-monitoring of eating behaviour; (b) controlling stimuli associated with eating; (c) physical activity; (d) nutrition education; (e) modifying self-defeating thoughts and negative emotions associated with dieting and body image; (f) setting realistic goals; (g) relationships; and (h) relapse prevention and weight maintenance. | Mild energy restriction weight loss programme (based on LEARN) paired with guided self-help | Adults aged 18-60, meeting DSM-4 criteria for binge-eating disorder and a BMI≥27 kg/m^2^ | Not specified (university clinic) | 3 months | X | NO | YES- minimal advice, no manual | North America | 46 ± 9.2 | 76.3% | 60.5% |
| Dassen et al., 2018  ^11^ | ***Weight loss plus cognitive training:***  The programme was developed as a game specifically designed to improve cognitive ability^43^. This training was developed by the faculty's engineering department in conjunction with the authors, and was based on the working memory (WM) training-paradigm ^44,45^. Participants had to perform 25 online WM sessions (experimental condition) or sham sessions (control condition) on a tablet or computer at home. Participants also received online psychoeducation about weight loss and a healthy lifestyle, while completing the 25 sessions of WM training at home. The lifestyle intervention included four themed sessions. The first session was available at the start of the training and focused on general principles of weight loss and motivation to lose weight. Participants were advised to keep track of their daily caloric intake via an online tool. The second session addressed topics such as the ‘obesogenic’ environment, healthy weight loss and nutrition, and participants were encouraged to come up with their own personal diet plan. The third lifestyle session addressed several aspects of physical activity, such as the health benefits of regular physical activity and implementing physical activities in daily life. The fourth session discussed strategies for dealing with difficult moments and gave tips to maintain a healthy weight after the intervention. The lifestyle intervention was composed by the first author, using general nutrition information and principles of cognitive behavioural therapy. | Mild energy restriction weight loss programme with cognitive training online | Adults aged 18-60, living with overweight, excluding participant in treatment for an eating disorder | Not specified | ~1 month | 7 months | NO | NO | Europe and the UK | 46.3 ± 11.9 | 76.5% | Not reported |
|  | ***Weight loss only:***  The lifestyle intervention included four themed sessions. The first session was available at the start of the training and focused on general principles of weight loss and motivation to lose weight. Participants were advised to keep track of their daily caloric intake via an online tool. The second session addressed topics such as the ‘obesogenic’ environment, healthy weight loss and nutrition, and participants were encouraged to come up with their own personal diet plan. The third lifestyle session addressed several aspects of physical activity, such as the health benefits of regular physical activity and implementing physical activities in daily life. The fourth session discussed strategies for dealing with difficult moments and gave tips to maintain a healthy weight after the intervention. The lifestyle intervention was composed by the first author, using general nutrition information and principles of cognitive behavioural therapy | Mild energy restriction weight loss programme |  | Not specified | ~1 month | 7 months | NO | NO |  | 50.1 ± 8.6 | 72.5% | Not reported |
| Grilo et al., 2013  ^12^ | ***Behavioural weight loss + orlistat***:  Pharmacotherapy included orlistat 120 mg 3 times daily, as a fixed-dose throughout the 4 month treatment. Medication clinical management procedures for orlistat^46^ were delivered in brief individual meetings by a bilingual psychiatrist at the community centre who was trained by the investigators. Participants were educated about orlistat and how it works, instructed to take the medication three times each day (with breakfast, lunch, and dinner), and were encouraged to follow the behavioural weight loss programme closely, particularly the reduced fat intake recommendations. Brief meetings with the study physician during the course of treatment were held as needed to review adherence, problem-solve issues of non-compliance, assess side effects, and if present, methods for coping with side effects. Patients were given a once-daily multivitamin containing fat-soluble vitamins and instructed to take it two hours prior to the study medication at dinner. BWL consisted of a culturally-enhanced adaptation of the Diabetes-Prevention-Programme delivered in Spanish by fully bilingual masters’ and doctoral-level clinicians at the community centre. The DPP^41^ focuses on goal-setting including reasonable weight loss, healthy eating behaviours and nutritional practices, lifestyle physical activity, and problem-solving to overcome barriers to achieve these lifestyle changes. Our adaptations included the use of handouts and examples geared to the Latino/a population of Connecticut (largely Puerto Rican and mixture of South American countries), and culture-specific food props to teach healthy portion size and combinations. Following initial training in BWL and DPP methods by the investigators, the clinicians participated in the cultural adaptation process jointly with the investigators, and subsequently received weekly supervision in BWL delivery by one of the investigators. Key features of the Lifestyle Balance programme included the following elements: 1) a goal-based behavioural intervention, 2) case managers or “lifestyle coaches” to deliver the intervention, 3) frequent contact and ongoing intervention throughout the trial to help participants achieve and maintain the weight and physical activity goals, 4) “toolbox” strategies to tailor the intervention to the individual participant, 5) intervention materials and strategies to address the needs of an ethnically diverse population, and 6) an extensive local and national network that provided training, feedback, and clinical support for the interventionists. The calorie goals were calculated by estimating the daily calories needed to maintain the participant’s starting weight and subtracting 500–1,000 calories/day (depending on initial body weight) to achieve a 1–2 pound per week weight loss. The fat goals, given in grams of fat per day, were based on 25% of calories from fat. Four standard calorie levels were used: 1,200 kcal/day (33 g fat) for participants with an initial weight of 120–170 lbs, 1,500 kcal/day (42 g fat) for participants with a weight of 175–215 lbs, 1,800 kcal/day (50 g fat) for participants with a weight of 220–245 lbs and 2,000 kcal/ day (55 g fat) for participants weighing >250 lbs. | Moderate energy restriction weight loss programme (based on DPP) with weight loss medication (orlistat) | Adults aged 21-65, with BMI ≥30 kg/m^2^, with binge eating disorder | Orlistat: bilingual psychiatrist at the community centre who was trained by the investigators.  BWL: fully bilingual masters’ and doctoral-level clinicians at the community centre | 4 months | 6 months | NO | NO | North America | 45.9 ± 9  47.8 ± 12.3 | 85%  85% | 100% |
|  | ***Behavioural weight loss + placebo:***  BWL consisted of a culturally-enhanced adaptation of the Diabetes-Prevention-Programme delivered in Spanish by fully bilingual masters’ and doctoral-level clinicians at the community centre. The DPP^41^ focuses on goal-setting including reasonable weight loss, healthy eating behaviours and nutritional practices, lifestyle physical activity, and problem-solving to overcome barriers to achieve these lifestyle changes. Our adaptations included the use of handouts and examples geared to the Latino/a population of Connecticut (largely Puerto Rican and mixture of South American countries), and culture-specific food props to teach healthy portion size and combinations. Following initial training in BWL and DPP methods by the investigators, the clinicians participated in the cultural adaptation process jointly with the investigators, and subsequently received weekly supervision in BWL delivery by one of the investigators. Key features of the Lifestyle Balance programme included the following elements: 1) a goal-based behavioural intervention, 2) case managers or “lifestyle coaches” to deliver the intervention, 3) frequent contact and ongoing intervention throughout the trial to help participants achieve and maintain the weight and physical activity goals, 4) “toolbox” strategies to tailor the intervention to the individual participant, 5) intervention materials and strategies to address the needs of an ethnically diverse population, and 6) an extensive local and national network that provided training, feedback, and clinical support for the interventionists. The calorie goals were calculated by estimating the daily calories needed to maintain the participant’s starting weight and subtracting 500–1,000 calories/day (depending on initial body weight) to achieve a 1–2 pound per week weight loss. The fat goals, given in grams of fat per day, were based on 25% of calories from fat. Four standard calorie levels were used: 1,200 kcal/day (33 g fat) for participants with an initial weight of 120–170 lbs, 1,500 kcal/day (42 g fat) for participants with a weight of 175–215 lbs, 1,800 kcal/day (50 g fat) for participants with a weight of 220–245 lbs and 2,000 kcal/ day (55 g fat) for participants weighing >250 lbs. Participants were also offered placebo 3 times daily, in a fixed dose throughout the 4 month treatment. | Moderate energy restriction weight loss programme (based on DPP) with placebo | Adults aged 21-65, with BMI ≥30 kg/m^2^ without binge eating disorder | BWL: fully bilingual masters’ and doctoral-level clinicians at the community centre | 4 months |  | NO | NO | North America | 45.6 ± 7.6  46.7 ± 10.5 | 70%  84% | 100% |
| Abiles et al., 2013  ^34^ | ***Behavioural weight loss + cognitive behavioural therapy (CBT):***  CBT was applied in three stages in consecutive groups of 10 patients each.  1st stage (Initial psychological assessment): Tests validated for Spanish-speaking populations were used to examine the psychological profiles of patients before their group assignment for the 2nd stage, considering four variables.  2nd stage (Group therapy): Over a three-month period, CBT was applied in 12 two-hour sessions using the method of Fairburn et al.^40^. The objective of the treatment was for the patient to recover self-esteem and develop appropriate eating behaviours for weight loss and long-term weight maintenance. It was necessary for patients to lose at least 10% of their initial weight to complete the CBT and be accepted for BS.  3rd stage (Individual monitoring and treatment): After the group therapy stage, patients again underwent psychopathology assessment to detect behaviours needing reinforcement or symptoms requiring individual treatment. Next, patients were followed up in weekly 60-min sessions for 12-months, and their suitability for BS was then evaluated in a final report. Throughout this 12 month period, the calorie intake of the patients was restricted by following a balanced diet of 1,500 kcal in accordance with guidelines provided by a nutritionist. | Moderate energy restriction weight loss programme paired with cognitive behavioural therapy | Adults aged 18-59, with binge eating disorder, in the pathway for bariatric surgery  Adults aged 18-59, without binge eating disorder, in the pathway for bariatric surgery | Nutritionist | 3 + 12 months | X | NO | NO | Europe and the UK | 42.6 ± 9.1  39.2 ± 9.1 | 65.3%  63.9% | Not reported |
| Grilo et al., 2020  ^13^ | ***BWL + pharmacotherapy***:  In this stepped care protocol, patients began behavioural weight loss (BWL) which lasted for 1 month. Treatment responders continued BWL, whereas non-responders switched to cognitive behavioural therapy, and all were randomized (double-blind) to weight-loss medication or placebo (5 months). BWL was based on the LEARN Program for Weight Management^39^. The widely used LEARN program encourages gradually losing weight, progressively increasing physical activity, and decreasing energy and fat intake through permanent lifestyle changes. The program emphasizes: (a) self-monitoring of eating behaviour; (b) controlling stimuli associated with eating; (c) physical activity; (d) nutrition education; (e) modifying self-defeating thoughts and negative emotions associated with dieting and body image; (f) setting realistic goals; (g) relationships; and (h) relapse prevention and weight maintenance. BWL was delivered in individual sessions ranging from 50 to 60 minutes following the manual and keyed to specific patient readings in their copy of the self-care manual and to weekly behavioural goals, moderate caloric reductions with improved nutrition quality (1,200- 1,500 kcal/day, less than 30% fat), and moderate increases in physical activity (30 minutes of physical activity five times weekly). Behavioural strategies include goal setting, recording food intake and physical activity, and problem solving to cope with barriers to attaining goals. Sibutramine, a serotonin/norepinephrine reuptake inhibitor, was given using fixed doses of 15 mg/d (38) during the last 5 months of Stepped Care. When sibutramine was withdrawn from the market (October 2010), the study was continued as conceptually designed using the FDA-approved weight-loss medication orlistat in a dose of 120 mg, three times daily. | Mild energy restriction weight loss programme (based on LEARN) with weight loss medication (sibutramine first but then changed to orlistat as sibutramine discontinued) | Adults aged 18-65, BMI 30-50 kg/m^2^ and meeting DSM-4 for binge eating disorder | Master’s-level clinicians delivered BWL, and a faculty-level psychiatrist delivered pharmacotherapy with minimal clinical management | 6 months | X | NO | NO | North America | 48 ± 9.6  50 ± 9.2 | 68%  82.1 % | 78.9%  77% |
|  | ***BWL + placebo***:  In this stepped care protocol, patients began behavioural weight loss (BWL) which lasted for 1 month. Treatment responders continued BWL, whereas non-responders switched to cognitive behavioural therapy, and all were randomized (double-blind) to weight-loss medication or placebo (5 months). BWL was based on the LEARN Program for Weight Management^39^. The widely used LEARN program encourages gradually losing weight, progressively increasing physical activity, and decreasing energy and fat intake through permanent lifestyle changes. The program emphasizes: (a) self-monitoring of eating behaviour; (b) controlling stimuli associated with eating; (c) physical activity; (d) nutrition education; (e) modifying self-defeating thoughts and negative emotions associated with dieting and body image; (f) setting realistic goals; (g) relationships; and (h) relapse prevention and weight maintenance. BWL was delivered in individual sessions ranging from 50 to 60 minutes following the manual and keyed to specific patient readings in their copy of the self-care manual and to weekly behavioural goals, moderate caloric reductions with improved nutrition quality (1,200- 1,500 kcal/day, less than 30% fat), and moderate increases in physical activity (30 minutes of physical activity five times weekly). Behavioural strategies include goal setting, recording food intake and physical activity, and problem solving to cope with barriers to attaining goals. Sibutramine, a serotonin/norepinephrine reuptake inhibitor, was given using fixed doses of 15 mg/d (38) during the last 5 months of Stepped Care. When sibutramine was withdrawn from the market (October 2010), the study was continued as conceptually designed using the FDA-approved weight-loss medication orlistat in a dose of 120 mg, three times daily.  Placebo was delivered in a double-blind (matched identical capsules) manner with minimal clinical management | Mild energy restriction weight loss programme (based on LEARN) with placebo |  | Master’s-level clinicians delivered BWL, and a faculty-level psychiatrist delivered pharmacotherapy with minimal clinical management | 6 months | X | NO | NO | North America | 45.9 ± 9.0 | 85% | 0% |
|  | ***BWL alone:***  BWL was based on the LEARN Program for Weight Management^39^. The widely used LEARN program encourages gradually losing weight, progressively increasing physical activity, and decreasing energy and fat intake through permanent lifestyle changes. The program emphasizes: (a) self-monitoring of eating behaviour; (b) controlling stimuli associated with eating; (c) physical activity; (d) nutrition education; (e) modifying self-defeating thoughts and negative emotions associated with dieting and body image; (f) setting realistic goals; (g) relationships; and (h) relapse prevention and weight maintenance. BWL was delivered in individual sessions ranging from 50 to 60 minutes following the manual and keyed to specific patient readings in their copy of the self-care manual and to weekly behavioural goals, moderate caloric reductions with improved nutrition quality (1,200- 1,500 kcal/day, less than 30% fat), and moderate increases in physical activity (30 minutes of physical activity five times weekly). Behavioural strategies include goal setting, recording food intake and physical activity, and problem solving to cope with barriers to attaining goals. | Mild energy restriction weight loss programme (based on LEARN) |  | Master’s-level clinicians delivered BWL | 6 months | X | NO | NO |  | 45.6 ± 7.6 | 70% |  |
| Loader et al., 2013  ^14^ | ***Behavioural weight loss + guided bibliotherapy self help:***  Behavioural weight loss was treatment as usual (TAU) consisted of the current support available from the weight management clinic. This support was initially a one-to-one appointment with a doctor and an invitation to attend a psycho-education group. This group provided information on factors affecting weight such as nutrition and activity levels. The initial group session lasted approximately an hour and a half and could include potentially 12 new patients. TAU was for an indeterminate period depending on the individual’s presentation. During their involvement with the clinic the average patient would attend regular assessment reviews with a doctor and a dietician and be invited to attend assessment group sessions. Support and advice was offered regarding diet, physical activity and lifestyle changes to encourage weight-loss.  The BWL was supplemented with guided self-help (GSH) bibliotherapy. The bibliotherapy manual was “Compassionate mind approach to beating overeating”^47^. Participants were asked to work through this manual at approximately one chapter every two weeks. They also received prearranged telephone support calls every two weeks facilitated by a therapist. Each telephone call was intended to offer support which focused on a relevant chapter of the manual. The calls were scheduled to last approximately 15 minutes and follow a protocol which was used as a prompt. If participants were unable to attend their telephone appointment then an alternative was arranged. The intervention lasted for six months and a maximum of 12 support telephone calls were offered. | Mild energy restriction weight loss with self-help guided compassion learning | Adults aged 18-65, BMI≥30 kg/m^2^, at a specialist NHS weight management clinic | BWL was delivered by a Dr and a dietitian. Therapists were Trainee Clinical Psychologists who had undergone training. Supervision was available from a Consultant Clinical Psychologist. | 6 months | X | NO | NO | Europe and the UK | 46.4 ± 9.7 | 66% | 88.9% |
|  | ***Behavioural weight loss alone:***  Behavioural weight loss was treatment as usual (TAU) consisted of the current support available from the weight management clinic. This support was initially a one-to-one appointment with a doctor and an invitation to attend a psycho-education group. This group provided information on factors affecting weight such as nutrition and activity levels. The initial group session lasted approximately an hour and a half and could include potentially 12 new patients. TAU was for an indeterminate period depending on the individual’s presentation. During their involvement with the clinic the average patient would attend regular assessment reviews with a doctor and a dietician and be invited to attend assessment group sessions. Support and advice was offered regarding diet, physical activity and lifestyle changes to encourage weight-loss. | Mild energy restriction weight loss |  | BWL was delivered by a Dr and a dietitian. | 6 months | X | NO | NO |  | 44.3 ± 9.5 | 72% | 61.1% |
| Nauta et al., 2000  ^15^ | ***Behavioural weight loss:***  The aim of the treatment was to learn a healthy eating pattern by having three meals each day and three planned snacks, decreasing fat intake, eating between 1,500 to 1,800 kcal a day, increasing exercise habits, and recognizing and anticipating high-risk situations. In the first session, the therapists explained the rationale of behavioural therapy: To regain control over eating, it is important to learn a healthy eating pattern, without excessive, restrictive dieting. Participants were told that treatment needed first to eliminate binge eating and overeating patterns by establishing regular, healthy eating patterns and that weight control needed to be a secondary concern. Participants were instructed to self-monitor their food intake and eating patterns, binge episodes, or episodes of overeating and the circumstances under which they occurred (i.e., time or mood). Examination of the self-monitoring formed an important focus of therapy. In the first half of the session, homework assignments were discussed, and in the second half, nutritional information was given. Furthermore, participants were advised to make gradual changes in eating habits and exercise level. The basic behavioural strategies were self-monitoring, goal-setting, and stimulus control techniques. Three forms of stimulus control techniques were used. First, participants learned stimulus control techniques whereby stimuli that resulted in eating were avoided (e.g., not going to a shop when hungry). Second, they learned techniques to anticipate high-risk situations and to outline strategies to prevent eating, such as walking or taking a shower. Third, participants learned self-reinforcement techniques, such as buying a magazine, and were positively reinforced by the therapist if they were not overeating. No attention was paid to dysfunctional cognitions. | Mild energy restriction weight loss programme with stimulus control techniques | Adults aged 18-50, with BMI≥ 27 kg/m^2^ and without binge eating  Adults aged 18-50, with BMI≥ 27 kg/m^2^ and meeting DSM-4 diagnostic criteria for binge eating | Therapists experienced in the behavioural and cognitive treatment of eating disorders and/ or obesity | 4 months | 10 months | NO | NO | Europe and the UK | 38.3 ± 7.1 | 100% | Not reported |
| Grilo et al., 2011  ^16^ | ***Behavioural weight loss:***  Behavioural weight loss was administered in 16 group 60-minute sessions over a 24-week period following the manualized LEARN Program for Weight Management^39^. This specific BWL is used widely in obesity studies^48^ and has been previously used in treatment trials with BED^49^. LEARN is an acronym for lifestyle, exercise, attitudes, relationships, and nutrition. LEARN focuses on making gradual lifestyle changes with goals of moderate caloric restriction and increased physical activity to produce gradual weight losses. The program emphasizes: (a) self-monitoring of eating behaviour; (b) controlling stimuli associated with eating; (c) physical activity; (d) nutrition education; (e) modifying self-defeating thoughts and negative emotions associated with dieting and body image; (f) setting realistic goals; (g) relationships; and (h) relapse prevention and weight maintenance. BWL was delivered in individual sessions ranging from 50 to 60 minutes following the manual and keyed to specific patient readings in their copy of the self-care manual and to weekly behavioural goals, moderate caloric reductions with improved nutrition quality (1,200- 1,500 kcal/day, less than 30% fat), and moderate increases in physical activity (30 minutes of physical activity five times weekly). Behavioural strategies include goal setting, recording food intake and physical activity, and problem solving to cope with barriers to attaining goals. | Mild energy restriction weight loss programme (based on LEARN) | Aged 18-60 with BMI 30-65 kg/m^2^, and meeting criteria for binge eating | Therapists (doctoral-level psychologists) | 6 months | 12 months | NO | NO | North America | 44.6 ± 8.5 | 62.2% | 80% |
|  | ***Sequential Cognitive Behavioural Therapy followed by Behavioural Weight Loss (CBT+BWL):***  This treatment condition involved a sequential approach in which CBT was delivered first (16 sessions over 16 weeks) followed by BWL (16 sessions over 24 weeks). The CBT and BWL interventions were delivered in group by the same therapists using the same BWL protocol as above. | Mild energy restriction weight loss programme (based on LEARN), paired with group cognitive behavioural therapy |  | Therapists (doctoral-level psychologists) | 10 months | 12 months | NO | NO | North America | 44.5 ± 9.2 | 80% | 74.3% |
| Masheb et al., 2011  ^17^ | ***Cognitive behavioural therapy (CBT) + low energy-density diet***:  In the programme, each hour-long session included 40 min devoted to CBT and 20 min devoted to dietary counselling. Between weeks 1-16, οne-hour weekly individual sessions were offered, whereas for weeks 17-26, sessions were offered every other week. Sessions included 40 min devoted to CBT and 20 min devoted to energy density. Participants completed daily food diaries that were checked weekly by clinicians. In addition, participants were instructed in how to self-monitor episodes of binge eating and overeating in these diaries. Additionally, participants in this condition received weekly dietary counselling for lowering energy density. In the first phase of this treatment, patients were informed about the objective and science of energy density shown a food preparation demonstration for a breakfast differing in energy density; shown photographs of meals differing in energy density; and taught how to calculate energy density using nutrition facts labels, the energy-density formula, and an energy-density value food chart. In the second phase of this treatment, goals were set to increase the patient’s consumption of low-energy-density foods which could be eaten in satisfying portions, and to exercise portion control over medium to high energy-density foods. Weekly topics adapted from the manualized protocol by Ello-Martin et al.^50^ were: water and energy density; reducing fat and energy density; volume and satiety; portion and serving sizes; carbohydrates and energy density; protein and energy density; calcium and energy density; beverages, alcohol and desserts; meal planning and grocery shopping; dining out and energy density; modifying recipes; overcoming obstacles to fruits and vegetables; cravings and variety; low-energy-density snacks and comfort foods; weight maintenance; and, holidays and celebrations | Mild energy restriction weight loss programme focussing on energy density, paired with cognitive behavioural therapy | Adults aged 21-60, BMI≥30 kg/m^2^, and meeting DSM-4 criteria for binge eating disorder | Doctoral-level research clinicians in psychology | 6 months | X | NO | NO | North America | 47.9 ± 7.9 | 80% | 72% |
|  | ***CBT plus general nutrition counselling not related to lowering energy density or weight loss:***  CBT- ED (21 hourly individual sessions over a six-month treatment period, weekly for weeks 1e16 and every other week for weeks 17e26, with 40 min devoted to CBT and 20 min devoted to nutrition). Participants in this condition completed food diaries and self-monitoring, and received the same exact CBT described in Treatment 1 above. Additionally, participants in this condition received weekly dietary counselling for health as opposed to reducing energy density or weight loss. A manual was specifically designed for this study so that the CBT þ GN would serve as an active comparator to the CBT þ ED treatment.1 In the first phase of the dietary component of treatment patients were: informed about the objective of the general nutrition treatment and the science and definition of nutrients; taught the definition of calories; and informed about nutrition facts labels. In the second phase, each weekly topic was designed specifically as a control for the type and amount of dietary information provided in the energy-density condition and included the following: MyPyramid.gov and your health; dietary fat and your health; fruits, vegetables and your health; portions, serving sizes and your health; carbohydrates and your health; protein and your health; calcium and your health; water and your health; meal planning, grocery shopping and your health; dining out and your health; added sugars and your health; herbs, spices and your health; consumer information and your health; sodium and your health; snacks, comfort foods and your health; vitamins, antioxidants and your health; and, tips for holidays and celebrations. Clinicians reviewed and discussed the weekly topic with patients, but no problem-solving or goal-setting was conducted in these sessions | Mild energy restriction weight loss advice with cognitive behavioural therapy | Adults aged 21-60, BMI≥30 kg/m^2^, and meeting DSM-4 criteria for binge eating disorder | Doctoral-level research clinicians in psychology | 6 months | X | NO | NO |  | 43.7 ± 6.7 | 72% | 88% |
| Munsch et al., 2007  ^18^ | ***Behavioural weight loss***:  6 group sessions lead by therapists. The active treatment phase consisted of 16 weekly 90 min group sessions. BWLT was based on the manual “Weight Loss with Xenical". This standardized treatment was developed to foster weight management and aims at instructing patients to normalize fat intake and to achieve balanced nutrition, including lifestyle modification and a hypocaloric diet (30% of energy from fat). | Mild energy restriction weight loss programme | Adults aged 18-70, BMI 27-40 kg/m^2^, meeting full DSM-IV criteria for binge eating disorder | Therapists (fully qualified psychotherapists + MSc students) | 4 months | X | NO | NO | Europe and the UK | 47.8 ± 11.8 | 86.1% | Not reported |
| Pataky et al., 2018  ^35^ | ***Multidisciplinary weight loss programme:***  The Service of Therapeutic Education for Chronic Diseases is a WHO collaborating and reference centre for chronic disease management, particularly engaged in therapeutic patient education in the field of obesity and diabetes. The evaluated lifestyle intervention for weight loss is a multidisciplinary and structured patient educational program for obese patients over 12 months, with a minimum of 9 group sessions and 10 individual outpatient appointments during the program duration. Each group session, of whole day duration  (from 9 a.m. to 5 p.m.) consists of several workshops having a specific structure, depending on the topic (e.g., diet, eating behaviour, physical activity). At the beginning of the program, patients are required to participate in a predefined curriculum including on medical issues, balanced diet, cognitive-behavioural therapy, physical activity, and art therapy over 4 consecutive days. During these 4 days, every patient has individual appointments with different healthcare providers (HCP) and specialists (e.g., physician, nurse, dietician, and psychologist) for medical, nutritional and psychological assessment on an interdisciplinary basis. Moreover, group sessions take place and are managed by the HCP according to the workshop’s topic. This 4-day (full time) educational program is followed by a coaching that takes place over the following 12 months. During this period, every patient participates in a minimum of 4 ambulatory, motivational and educational group sessions (every 2–3 months), each with different topic (i.e., diet, cognitive-behavioural therapy, physical activity, and body image) taking 1 day each. A personal coach (i.e., an HCP specialized in both obesity management and patient education) is attributed to every patient. The patient has a follow-up on regular monthly basis with this HCP and during the 12-month follow-up period. His/her body weight is measured monthly during each 60-min individual appointment. During these face-to-face meetings, different aspects of healthy nutrition and physical activity are discussed with the patient, as well as the difficulties related to psychological and environmental issues (e.g., family, work, social live). The face-to-face meetings are individually tailored and are based on motivational interviewing, and any diet or physical activity prescription is proposed to the patient. At the end of the 12-month period, patients are required to participate in the last workshop day centred on results after 12 months. In addition to biological evaluation (e.g., fasting glucose, HbA1c, lipids, liver parameters, and blood pressure), group sessions and individual appointments take place during the day in order to assess the psychosocial well-being and quality of life for every participant. | Mild energy restriction weight loss outpatient programme | Adults living with overweight/obesity presenting for treatment | Multidisciplinary team including a dietitian | 12 months | X | NO | NO | Europe and the UK | 46 ± 8  47 ± 11  46 ± 9 | 80%  87.2%  84.6% | Not reported |
| Preuss et al. 2017  ^19^ | ***Weight loss programme***:  Treatment focuses on therapeutic goals recommended for both patients with BED and patients with obesity and other pathological eating behaviour, by evidence-based clinical guidelines (NICE, 2014 for obesity and NICE, 2004/2017 for atypical EDs including BED). Ten modules concerning the management of daily diet, movement und stress were constructed, based on previously evaluated CBT manuals for the treatment of obesity and BED^40,51^. Typical interventions were application of daily eating protocols and goal attainment by formulating weekly goals concerning eating and activity behaviours, promoting change in dietary habits, e.g., increasing intake of complex carbohydrates and high-fibre products, increasing daily physical activity, learning mindful eating, applying self- instruction and problem solving skills to resolve emotional eating and incorporating relaxation exercises into daily life (e.g. progressive muscular relaxation and imagery techniques). | Mild energy restriction weight loss programme | Adults with BMI 25-40.9 kg/m^2^ | Clinical psychologists in advanced training for the licensure of psychological psychotherapy | ~3 months | 6 months | NO | NO | Europe and the UK | 38.1 ± 10.7  38.1 ± 11.2  38.1 ± 10.1 | 94.2%  95.1%  92.9% | Not reported |
| Ramirez et al., 2001  ^20^ | ***Weight loss***: This intervention consisted of 16 sessions, one hour per week, led by a registered dietitian. The curriculum for the weight-control intervention as the LEARN Program for Weight Control^39^, a 16-week nutrition and behavioural self-management program that L incorporates a weekly plan to promote eating and exercise change. (The edition of the LEARN program we used did not include any intervention for body image, whereas subsequent editions contain a brief section on this aspect.) | Mild energy restriction (based on LEARN) | Adults with BMI greater or equal to 27.3 for women and 27.8 for men | Registered dietitian | 4 weeks | X | NO | NO | North America | 44 ± 9.7 | 77.3% | Not reported |
| Rock et al., 2010  ^21^ | ***Centre-based weight loss intervention:***  Participants assigned to the centre based study groups received all program materials, including free-of-charge pre-packaged prepared foods as needed to achieve a meal plan. Interactions between corporate trained and supervised staff and the participants consisted of brief weekly one-to-one contacts with an in-person or telephone counsellor, with follow-up phone and e-mail contacts and Web site or message board availability. Counsellors were instructed to provide the program as designed for a regular paying client, although they were not blinded to the identity of study participants. Free of-charge counselling sessions were offered to participants for the entire 2-year period. The diet component of the program consisted of a nutritionally adequate, low-fat (20%-30% of energy), reduced energy diet (typically 1200-2000 kcal/d) that included pre-packaged prepared food items with increased amounts of vegetables and fruits to reduce the energy density of the diet. The approach was tailored so that participants could choose regular foods when preferred. Participants were encouraged during the initial period to follow a menu plan with pre-packaged foods, which would provide 42% to 68% of energy for those who choose not to deviate from the plan. Regular foods, such as vegetables, fruit, cereal or grain products, low-fat dairy products, lean meat or the equivalent, and unsaturated fat sources were recommended to achieve the total prescribed energy intake. Over time, participants were transitioned to a meal plan based mainly on food not provided by the commercial program, although participants could choose to include 1 pre-packaged meal per day during weight loss maintenance. Prepared foods and counsellors were provided by Jenny Craig Inc (Carlsbad, California). Increased physical activity was another program component; the goal was 30 minutes of physical activity on 5 or more days per week. Program material and counselling addressed attitudes about weight, food, and physical activity and included recipes and guidance for eating in restaurants, CDs and DVDs to increase physical activity, and online tools and support | Moderate energy restriction weight loss programme delivered in person | Females aged 18 or older, with a BMI 25-40 kg/m^2^, without a diagnosis of an eating disorder | Corporate trained and supervised staff | ~ 6 months intensive, 2 years overall | 12 months | NO | YES- publically available materials | North America | 44 ± 10 | 100% | 67.7% |
|  | ***Telephone-based weight loss intervention:***  Participants assigned to the telephone based study groups received all program materials, including free-of-charge pre-packaged prepared foods as needed to achieve a meal plan. Interactions between corporate trained and supervised staff and the participants consisted of brief weekly one-to-one contacts with an in-person or telephone counsellor, with follow-up phone and e mail contacts and Web site or message board availability. Counsellors were instructed to provide the program as designed for a regular paying client, although they were not blinded to the identity of study participants. Free of-charge counselling sessions were offered to participants for the entire 2-year period. The diet component of the program consisted of a nutritionally adequate, low-fat (20%-30% of energy), reduced energy diet (typically 1200-2000 kcal/d) that included pre-packaged prepared food items with increased amounts of vegetables and fruits to reduce the energy density of the diet. The approach was tailored so that participants could choose regular foods when preferred. Participants were encouraged during the initial period to follow a menu plan with pre-packaged foods, which would provide 42% to 68% of energy for those who choose not to deviate from the plan. Regular foods, such as vegetables, fruit, cereal or grain products, low-fat dairy products, lean meat or the equivalent, and unsaturated fat sources were recommended to achieve the total prescribed energy intake. Over time, participants were transitioned to a meal plan based mainly on food not provided by the commercial program, although participants could choose to include 1 pre-packaged meal per day during weight loss maintenance. Prepared foods and counsellors were provided by JennyCraig Inc (Carlsbad, California). Increased physical activity was another program component; the goal was 30 minutes of physical activity on 5 or more days per week. Program material and counselling addressed attitudes about  weight, food, and physical activity and  included recipes and guidance for eating in restaurants, CDs and DVDs to increase physical activity, and online tools and support. | Moderate energy restriction weight loss programme delivered over the phone |  | Corporate trained and supervised staff | ~ 6 months intensive, 2 years overall | 12 months | NO | YES- publically available materials | North America | 44 ± 10 | 100% | 79.3% |
| Barnes et al., 2017  ^22^ | ***Weight loss + motivational interviewing programme:***  Intervention) included five manualized sessions over 12 weeks, with guidelines to help medical assistants flexibly apply MI strategies to motivate patients for behaviour changes that support weight loss. The guidelines allowed focus on BED as needed. The first appointment included an initial 60-minute in-person individual session. Following this first appointment, patients received up to four additional 20-minute MI sessions. At their first session, participants also received a Lifestyle, Exercise, Attitudes, Relationships, and Nutrition (LEARN) manual^39^ and orientation to a free website for tracking food intake, setting weight and intake goals, and physical activity (Livestrong.com). | Mild energy restriction weight loss programme (based on LEARN) paired with motivational interviewing | Adults with BMI 25-55 kg/m^2^ | Medical assistants | 3 months | 6 months | NO | YES- no intervention offered, usual care in community if they want | North America | 47.1 ± 10 | 80% | 63% |
|  | ***Nutrition psychoeducation programme:***  The nutrition psychoeducation and internet condition was designed as a five-session psychoeducation only, attention-control. The sessions provided basic nutritional information (e.g., recommended fruit/vegetable intake) based on the recommendations of the American Heart Association and United States Department of Agriculture and allowed patients to ask questions to better understand the material. Participants received the same manual, and opportunity to sign up and use Livstrong.com to set weight loss/calorie goals as above. However, any further discussion about motivation, food tracking, goal setting, or personalized feedback was proscribed. | Mild energy restriction weight loss nutrition psychoeducation programme (based on the American Heart Association and the US department of Agriculture) |  | Medical assistants | 3 months | 6 months | NO | YES- no intervention offered, usual care in community if they want | North America | 49 ± 11.6 | 69% | 69% |
| Barnes et al., 2018  ^36^ | ***Motivational Interviewing and Nutrition Psychoeducation:***  This was designed as a five session, manualized, 3-month intervention. MINP included guidelines to help medical assistants flexibly apply MI with strategies to motivate participants for weight-related behaviour change and allowed focus on BED as needed. The first session was an initial 60-min in person individual appointment, which focused on enhancing motivation for weight loss and treatment adherence and ending with participants setting self-identified specific weight-related goals. Clinicians interacted with their participants in a non-judgmental and collaborative fashion, conveying respect, acceptance, and compassion toward them and a stance toward evoking the participants' motives for change, consistent with a MI style of interaction. Use of MI-inconsistent strategies (e.g., confrontation) was proscribed. At the participants' discretion, the session also included basic nutrition psychoeducation (e.g., recommended fruit/vegetable intake, healthy portion and serving sizes) based on the recommendations of the American Heart Association and United States Department of Agriculture. In addition, they received training in the use of supplemental materials: a free weight loss website (Livestrong.com) and a LEARN manual^39^, a readily available, well-researched weight loss manual. Clinicians taught participants to login to Livstrong.com, enter pertinent information (height, weight, age, activity level) and weekly weight loss and physical activity goals. Livestrong.com then provided participants with daily calorie guidelines for attaining their goals. Participants were shown how to track food, weight, and exercise, and to monitor other nutrition related information (e.g., carbohydrate intake), and if participants desired, they received personalized feedback on food journals at subsequent sessions. Following this first appointment, participants received up to four additional 20-min MINP sessions (in-person at weeks 6, 12, by phone at weeks 3, 9). If participants were interested, each session started with a discussion of the participants' specific and measurable behavioral weight-loss goals (e.g., walk 30 min 3 times a week, track food intake on Livestrong.com 4 days a week) from the previous session and ended with setting new goals, including problem-solving as necessary for when goals were not reached. Clinicians used MI strategies (e.g., reflections, affirmations, change planning) in these sessions to enhance participant motivation to meet weight-related goals (e.g., decreasing calories, increasing fruit/vegetable intake, increasing physical activity) and provided nutrition psychoeducatio***n.*** | Mild energy restriction weight loss programme (based on LEARN) paired with motivational interviewing and nutritional psychoeducation | Adults with BMI 25-55 kg/m^2^ | Medical assistants | 3 months | 6 months | NO | NO | North America | 48.4 ± 10.6 | 87.1% | 41.9% |
| Moss et al., 2017  ^23^ | ***Behavioural weight-loss programme:***  TrymGym was established in 1973 at a large medical doctoral university in Calgary and over 10,000 participants have completed the program. This BWLP emphasizes gradual, sustainable weight loss and lifestyle changes, and is delivered by a team of health care practitioners including dietitians, kinesiologists, and fitness instructors via both classroom sessions and exercise sessions. Specifically, the program consists of three core components: (1) Nutrition: individualized guidelines for healthy eating, based on the Canada Food Guide^52^, were developed for each participant, (2) Physical activity: group exercise classes focused on fat loss, strength training, and development of endurance and flexibility, and (3) Behaviour change: behavioural strategies including self-monitoring, goal-setting, and formulating action plans to achieve goals were taught in classroom sessions. The semi-structured MI protocol was a 45-min intervention developed by the first author based on general MI principles and guidelines^53^, MI strategies specific to health care practice^54^, and MI principles for and (8) eliciting ideas for possible changes participant could make to work towards weight loss. The protocol for both MI sessions consisted of similar components for obesity treatment^55^. The MI protocol included the following components: (1) eliciting concerns about weight, (2) exploring ambivalence, (3) assessing importance and confidence for change, (4) writing a decisional balance, (5) bolstering self-efficacy, (6) looking towards the future. | Mild energy restriction weight loss programme with exercise | Adults with BMI ≥25 kg/m^2^ | Researcher/therapist | 3 months | 9 months | NO | NO | North America | 45.6 ± 9.8 | 75.36% | 95.8% |
|  | ***Weight loss attention control:***  The attention control interview was a semi-structured interview addressing health history, weight history, diet history, and dietary and physical activity habits. Most questions were drawn from the TrymGym intake application. It was designed to be structurally equivalent to the MI session in length of session, timing of sessions, and treatment modality. The goal was to provide a pseudo-intervention that controlled for factors common to attending treatment (e.g., attending treatment sessions, having personal contact with a therapist, discussing weight-related issues). | Mild energy restriction weight loss |  | Researcher/therapist | 3 months | 9 months | NO | NO | Europe and the UK | 44 ± 11.9 | 84% | Not reported |
| Werrij et al., 2008  ^24^ | ***Cognitive dietetic group treatment:***  The treatment consisted of 10 weekly sessions of 2 h each. Both were provided in groups with a maximum of 12 participants. Each treatment session was divided into two parts. The first part (the first hour) was always the dietetic intervention, carried out by dieticians. This dietetic treatment part was exactly the same for both treatment conditions. The main aims of the dietetic intervention were to change unhealthy dietary patterns into more healthy ones and to improve self-control. To achieve this, nutritional education was provided, food diaries were kept, and cooking classes were given to learn healthy cooking. Dietary patterns were changed by a stepwise program, aimed at eating three meals a day, eating at a regular place, and mindful eating; slow, conscious, and without distraction. Participants were trained in changing bad eating habits into more healthy habits; they learned when to stop eating, how to refuse food, how to find social support, how to deal with parties and super market shopping, and so on. They further received guidelines for a healthy diet but this was not a prescribed diet. Interventions in the second part (the second hour) differed between the experimental and the control treatment; in the experimental CDT condition cognitive therapy (CT) was added to the dietetic treatment, whereas in the control EDT condition physical exercise was added to the dietetic intervention. The cognitive therapy was performed by fully qualified cognitive behaviour therapists. Aims of the CT were to identify, challenge, and change dysfunctional cognitions concerning eating, control, weight, and shape, as well as related schemas (e.g., self-esteem schemas or interpersonal schemas). Automatic thoughts and beliefs were identified and challenged, and behavioural experiments were set up. Participants were provided with workbooks entitled Dik Tevreden (Pleasantly Plump) containing background information about the cognitive intervention and homework assignments, including thought diaries. An example of a frequently reported dysfunctional thought related to control overeating was “whenever I start eating nuts [chocolates, candies, etc.], I have to finish the whole bowl”. An example of a dysfunctional thought related to weight was “when I am this fat, I will never find nice clothes”. The CBT therapists were intensely trained into the present CT protocol by the authors who are fully qualified CBT therapists and experienced in the cognitive treatment of eating disorders and obesity. There were weekly supervision sessions. | Mild energy restriction weight loss programme with cognitive therapy | Adults aged 18-65 and BMI≥27 kg/m^2^ | Dietitians and fully qualified CBT therapists | 2.5 months | 12 months | NO | NO | Europe and the UK | 44 ± 11.9 | 84% | Not reported |
|  | ***Exercise + dietetic treatment (EDT):***  The treatment consisted of 10 weekly sessions of 2 h each. Both were provided in groups with a maximum of 12 participants. Each treatment session was divided into two parts. The first part (the first hour) was always the dietetic intervention, carried out by dieticians. This dietetic treatment part was exactly the same for both treatment conditions. The main aims of the dietetic intervention were to change unhealthy dietary patterns into more healthy ones and to improve self-control. To achieve this, nutritional education was provided, food diaries were kept, and cooking classes were given to learn healthy cooking. Dietary patterns were changed by a stepwise program, aimed at eating three meals a day, eating at a regular place, and mindful eating; slow, conscious, and without distraction. Participants were trained in changing bad eating habits into more healthy habits; they learned when to stop eating, how to refuse food, how to find social support, how to deal with parties and super market shopping, and so on. They further received guidelines for a healthy diet but this was not a prescribed diet. Interventions in the second part (the second hour) differed between the experimental and the control treatment; in the experimental CDT condition cognitive therapy (CT) was added to the dietetic treatment, whereas in the control EDT condition physical exercise was added to the dietetic intervention. The cognitive therapy was performed by fully qualified cognitive behaviour therapists and fully qualified physiotherapists led the physical exercise. Participants in the control condition engaged in a 1-h low intensity exercise program (gym) supervised by a qualified physiotherapist. The combination of dietetic treatment (1 h) and physical exercise (1 h) in groups is, in this Dutch field setting, the standard treatment for obesity. | Mild energy restriction weight loss programme and exercise |  | Dietitians and fully qualified physiotherapists | 2.5 months | 12 months | NO | NO | Europe and the UK | 45 ± 12.2 | 78% | Not reported |
| Wilson et al., 2010  ^25^ | ***Behavioural weight loss:***  The National Institutes of Diabetes and Digestive and Kidney Diseases’ Diabetes Prevention Program’s manual^41^ was adapted for this study. The program includes both moderate caloric restriction and exercise. The treatment initially focused on dietary change toward a weight loss goal of 7% of one’s starting weight. Participants were first asked to reduce fat intake to 25% of calories from fat. If satisfactory progress in terms of weight loss was not being made a calorie goal was set based on initial weight. Self-monitoring of exercise, fat intake, and (if necessary) caloric intake is an essential aspect of the programme. The exercise goal was 2.5hours of moderate exercise each week. The core curriculum consists of 16 individual weekly sessions each lasting 50 minutes and followed by 4 sessions at 2-week intervals aimed at continuing weight loss and enhancing maintenance of such losses based on the National Institutes of Diabetes and Digestive and Kidney Diseases manual for contacts after the initial core 16 sessions. The treatment was delivered by master’s-level therapists in clinical psychology or nutrition, 2 at Rutgers University and 3 at Washington University. Bonnie Gillis, MS, RD, conducted the initial training work shop and served as a consultant. The therapists received supervision every other week. | Moderate energy restriction weight loss programme (based on DPP) | Adults with BMI 27-45 kg/m^2^ and meeting DSM-4 for binge eating disorder | Master’s-level therapists in clinical psychology or nutrition | 6 months | 12 months | NO | NO | North America | 46.2 ± 10.9 | 89% | 88% |
| Allison et al., 2022^26^ | Liraglutide (6.0 mg/ml) was provided as pre‐ filled, disposable, injection pens (Novo Nordisk A/S). A study physician or nurse practitioner instructed participants how to properly perform daily subcutaneous injections into their abdomen, thigh, or upper arm, and participants were given an instruction card detailing how to administer the medication. To reduce the likelihood of gastrointestinal symptoms (e.g., nausea, vomiting), the medication was  initiated at 0.6 mg/day for 1 week and then increased by 0.6 mg/day in weekly intervals until a dose of 3 mg/day was reached (over the  course of 5 weeks). If participants missed more than 3 days, they were to initiate therapy at 0.6 mg/day again to avoid gastrointestinal  symptoms. Participants who did not tolerate an increased dose during escalation had a delayed dose escalation by up to 7 days. This is the  same dosing regimen as that used for obesity treatment. Participants then continued administration of the injections for an additional  12 weeks at full strength. | Medication with lifestyle modification | Adults aged 21–70 years old with a BMI ≥ 27 kg/m^2^, required to meet full criteria for BED as  measured by the Eating Disorder Examination (EDE) (interview version, 16th edition) | Unspecified | 4 months | x | NO | YES- placebo | North America | 46.3 ± 7.8 | 46% | 62% |
|  | Placebo | Control |  |  |  |  |  |  |  | 42.8 ± 12.5 | 79% | 57% |
| Grilo et al., 2022^27^ | BWL followed the SMART stepped protocol. BWL was based on the LEARN Program for Weight Management. The widely used LEARN program encourages gradually losing weight, progressively increasing physical activity, and decreasing energy and fat intake through permanent lifestyle changes. The program emphasizes: (a) self monitoring of eating behavior; (b) controlling stimuli associated with eating; (c) physical activity; (d) nutrition education; (e) modifying self defeating thoughts and negative emotions associated with dieting and body image; (f) setting realistic goals; (g) relationships; and (h) relapse prevention and weight maintenance. BWL was delivered in individual sessions ranging from 50 to 60 minutes following the manual and keyed to specific patient readings in their copy of the self-care manual and to weekly behavioral goals. moderate caloric reductions with improved nutrition quality (1,200- 1,500 kcal/d, less than 30% fat), and moderate increases in physical activity (30 minutes of physical activity five times weekly). Behavioral strategies include goal setting, recording food intake and physical activity, and problem solving to cope with barriers to attaining goals. BWL was delivered in individual 45-minute sessions following the manualized protocol. Participants were given patientversion manuals covering all the BWL information and components. Weekly homework assignments were keyed to specific material to reinforce learning and using behavioral techniques. BWL focuses on gradual behavioral lifestyle changes, including moderate caloric decreases (with a goal of approximately 1500 kcal/day), improved nutrition quality (,30% fat), and moderate physical activity (30 minutes five times weekly). Behavioral techniques include goal setting, monitoring food intake and physical activity, stimulus control to achieve and maintain the lifestyle changes, and problem-solving skills to overcome challenges. BWL was delivered by 11 research clinicians with programmatic interests in eating disorders and obesity; six were clinical psychology graduate students (with a mean of 4.3 years of graduate education) and five were postdoctoral psychologists (with a mean of 8.0 years of years graduate education). Clinicians received intensive training in the manualized protocols and were supervised weekly (including reviews of recorded sessions) by the investigators to monitor quality and adherence. Supervision included review of the structure, process, and content of sessions to ensure fidelity and to prevent drift, per previous trials. | Behavioural weight loss + placebo | Meeting DSM-5 criteria for binge eating disorder, age between 18 and 70 years, and a body mass index (BMI) between 30.0 and 50.0 (or ≥27.0 with obesity-related comorbidity) | Research clinicians with programmatic interests in eating disorders and obesity; six were clinical psychology graduate students (with a mean of 4.3 years of graduate education) and five were postdoctoral psychologists (with a mean of 8.0 years of years graduate education). | 4 months | N/A | NO | Yes-placebo | North America | 46 **±** 11.9 | 80% | 74.3% |
|  | The naltrexone-bupropion combination comprised 32 mg/day of sustainedrelease naltrexone and 360 mg/day of sustained-release bupropion; two tablets were taken twice daily, each containing 8 mg of naltrexone and 90 mg of bupropion. Placebo was given in capsules matched in appearance and frequency. Naltrexone-bupropion dosing began with one quarter of the full dose and was increased weekly until full dose was achieved by the fourth week. BWL followed the protocol originally developed and refined for obesity trials and since adapted for binge-eating disorder (12, 13). BWL was delivered in individual 45-minute sessions following  the manualized protocol. Participants were given patientversion manuals covering all the BWL information and components. Weekly homework assignments were keyed to specific material to reinforce learning and using behavioral  techniques. BWL focuses on gradual behavioral lifestyle changes, including moderate caloric decreases (with a goal of approximately 1500 kcal/day), improved nutrition quality (,30% fat), and moderate physical activity  (30 minutes five times weekly). Behavioral techniques include goal setting, monitoring food intake and physical activity, stimulus control to achieve and maintain the lifestyle changes, and problem-solving skills to overcome  challenges. BWL was based on the LEARN Program for Weight Management. The widely used LEARN program encourages gradually losing weight, progressively increasing physical activity, and decreasing energy and fat intake through permanent lifestyle changes. The program emphasizes: (a) self monitoring of eating behavior; (b) controlling stimuli associated with eating; (c) physical activity; (d) nutrition education; (e) modifying self defeating thoughts and negative emotions associated with dieting and body image; (f) setting realistic goals; (g) relationships; and (h) relapse prevention and weight maintenance. | Behavioural weight loss + naltrexone-bupropion |  |  |  |  |  |  |  |  | 47 ± 13 | 82.9% |
|  | Placebo | Control |  |  |  |  |  |  |  | 46.94 ± 12.6 | 82.4% | 76.5% |
| Grammer et al., 2023^28^ | The combined intervention included all components of the Student Bodies-Eating Disorders program in addition to key behavioral principles of BWL (e.g., change in diet and physical activity, goal setting, selfmonitoring) as outlined by the U.S. Preventive Services Task Force guidelines for behavioral weight loss interventions (US Preventive Services Task Force et al., 2018). Balanced eating content focused on energy density psychoeducation adapted from a well-established volumetrics approach to reducing energy-dense foods and increasing nutrient-dense foods as a method to promote fullness and achieve a caloric deficit to aid weight change. Participants were encouraged to self-monitor daily dietary adherence in the online platform or another tracking method of their choice. Psychoeducation was provided to illustrate the benefits of physical activity to promote health and wellness but not as an effective strategy to initiate weight loss. At each session, participants in the combined intervention were asked to report on their weekly goals as well as report whether they increased nutrient-dense foods, decreased energy-dense foods, and whether their weight increased, decreased, or stayed the same from the previous week | Behavioural weight loss and cognitive behavioural therapy | Eligible participants were aged 18–39, self-reported ≥6 objectively large binge episodes in the past 3 months, had a BMI ≥25, and reported interest in losing weight | ? online coaches | 2 months | N/A | NO | NO | North America | No identifiable baseline data collected | | |
| Mohseni et al., 2023^38^ | Throughout the intervention, patients received 18 group sessions consisting of 1.5 hours of combined nutritional advice and CBT-based  psychoeducation (provided by a dietician and a psychologist, respectively). These were followed by an exercise session consisting of 1.5 hours of aerobic and anaerobic exercise (guided by a physical therapist). Session frequency was gradually tapered from weekly at the beginning (weeks 1 - 10) to meetings every three months at a later stage of the program (week 25 until 1.5 years). The sessions were held in a meeting room (CBT group sessions with dietician and psychologist together) and, after that, the gym (exercise sessions) of the Erasmus Medical Center or at similar meeting rooms/sports hall at the Erasmus University | Behavioural psychoeducation and nutritional advice | BMI ≥30 kg/m2, age ≥ 18 years, sufficient treatment adherence (e.g. ≥ three sessions missed), and presence of at least one obesity-related comorbidity (e.g., hypertension, type 2 diabetes, dyslipidemia, non-alcoholic fatty liver disease or obstructive sleep apnoea) | Dietitians, Psychologists. physical therapist | 15 months | N/A | NO | NO | Europe and the UK | 42 ± 13 | 76% | 84.4% |
| da Luz et al., 2024^29^ | The treatment protocol for the online sessions of HAPIFED was developed based on the HAPIFED manual. All sessions included interventions delivered by psychologists, dietitians, and/or exercise physiologists that aimed to reduce participants’ eating disorder behaviours and improve their poor weight management (e.g., strict dieting, binge eating, lack of planning/organization, sedentarism). | Weight loss intervention and cognitive behavioural therapy | (1) BED according to the DSM 5 criteria [35]; (2) age ≥ 18 years; (3) access to a computer with internet; (4) access to a private room during sessions; (5) being literate; (6) access to a scale and stadiometer to measure weight and height; (7) time available to complete the program; and (8) BMI ≥ 27 and <45 kg/m^2^ | All sessions included interventions delivered by psychologists,  dietitians, and/or exercise physiologists | 3 months | 9 months | NO | NO | South America | 36.5 ± 9.8 | 91.8% | 72.1% |
| Carbone et al., 2024^37^ | Participants initiated treatment with NB, taking one tablet per day, which contained 8 mg of naltrexone·HCl and 90 mg of bupropion·HCl. The dosage was gradually increased, reaching a maximum of two tablets twice a day by the fourth week. If patients experienced manageable side effects such as constipation, tinnitus, or nausea, the therapy dose was reduced to the minimum level needed for effectiveness. Patients were advised to maintain their usual eating and daily routines, except for engaging in moderate aerobic physical activity. Additionally, behavioural counselling to promote a healthy lifestyle was provided to each participant throughout the entire treatment duration | Medication and nutritional advice | A body mass index (BMI) ≥ 30 kg/m2, an age ≥18 years and <65, ability to answer self-report questionnaires and ability to give valid informed consent to the study | Research clinician | 4 months | N/A | NO | NO | Europe and the UK | 42 ± 12.5 | 82% | ?100% |
| Rahimi-Ardabili et al., 2024^30^ | The nutrition information was based on the Australian Dietary Guidelines 201334 providing information on body regulation and hunger and replacing nutrient-poor food with nutrient-rich food groups. Participants in the intervention group also received information about self-compassion that was partially adapted from Neff’s website and her book, encouraging individuals to treat themselves as a good friend in times of suffering. For the current study, information about self-compassion was modified to address participants’ needs, that is, assisting them with weight-related issues such as body dissatisfaction and a need for self-care. Participants were advised to practice self-compassion every day. At the beginning of each four-week period, participants in the intervention group were advised to adopt two goals (one for nutrition and one for self-compassion) based on the monthly information they received and track their progress on these goals using the online application over the 12-week study period. A list of goal options was available for each topic to guide participants in setting goals. Participants could choose one of the goal options, modify one of those goals or set their own goals. The investigator also provided monthly feedback on newly set goals by reviewing each participant’s goals and emailing feedback to the participant. | Weight loss + compassionate training | Aged 18 - 55 years; BMI of 25 - 40 kg/m^2^; able to run internet browser for at least one hour per week; able to read and write English | Online, therapists unspecified | 3 months | N/A | NO | Yes- minimal intervention | Australia and New Zealand | Not reported | 84% | 26% |
|  | Participants in the control group received an email every four weeks that provided only a brief form of standard nutrition information in PDF format (three emails in total: Week Zero, Week Four and Week Eight). The nutrition information provided to the control group was similar to the core part of the nutrition information provided to the intervention group. This group did not receive any information on goal-setting or self-compassion, nor did they have access to the study website or online Goal Tracker. | Control |  |  |  |  |  |  |  |  | 86% | 36% |

#### Appendix D. List of studies excluded at full-text screening stage, with brief reasons

| **Studies** | **Reason for exclusion** |
| --- | --- |
| - Cargill, B. R., Clark, M. M., Pera, V., Niaura, R. S., & Abrams, D. B. (1999). Binge eating, body image, depression, and self‐efficacy in an obese clinical population. *Obesity research*, *7*(4), 379-386. - Morrison, A. L. (1997). *The effects of alternative group interventions on physical self-esteem in obese women*. Washington State University. - Olson, K. L., Neiberg, R. H., Tate, D. F., Garcia, K. R., Gorin, A. A., Lewis, C. E., ... & Wing, R. R. (2018). Weight and shape concern impacts weight gain prevention in the SNAP trial: Implications for tailoring intervention delivery. *Obesity*, *26*(8), 1270-1276. - Legenbauer, T., Müller, A., de Zwaan, M., & Herpertz, S. (2020). Body image and body avoidance nine years after bariatric surgery and conventional weight loss treatment. *Frontiers in psychiatry*, *10*, 494905. - Moulos, I., Maramis, C., Mourouzis, A., & Maglaveras, N. (2015). Designing the user interfaces of a behavior modification intervention for obesity & eating disorders prevention. In *Digital Healthcare Empowering Europeans* (pp. 647-651). IOS Press. - Masheb, R. M., Lutes, L. D., Myra Kim, H., Holleman, R. G., Goodrich, D. E., Janney, C. A., ... & Damschroder, L. J. (2015). High‐frequency binge eating predicts weight gain among veterans receiving behavioral weight loss treatments. *Obesity*, *23*(1), 54-61. - Rutigliano, G., Briganti, E., Chatzianagnostou, K., Quiñones-Galvan, A., Arvia, C., Iervasi, G., & Dell’Osso, L. (2014). Magnesium supply improves adherence to hypocaloric diets in women: Effects on weight control competence. *Eur Neuropsychopharmacol*, *24*(2), S741. - Lydecker, J. A., & Grilo, C. M. (2022). Psychiatric comorbidity as predictor and moderator of binge-eating disorder treatment outcomes: an analysis of aggregated randomized controlled trials. *Psychological Medicine*, *52*(16), 4085-4093. - Kalaria, S. N., McElroy, S. L., Gobburu, J., & Gopalakrishnan, M. (2020). An innovative disease‐drug‐trial framework to guide binge eating disorder drug development: a case study for topiramate. *Clinical and Translational Science*, *13*(1), 88-97. - Amosova, M. V., Gurova, O. Y., & Fadeev, V. V. (2018, October). Predictors of glucose-lowering response to treatment with glucagon-like peptide-1 receptor agonists in patients with diabetes and obesity. In *DIABETOLOGIA* (Vol. 61, pp. S361-S361). 233 SPRING ST, NEW YORK, NY 10013 USA: SPRINGER. - Gudbergsen, H., Overgaard, A., Henriksen, M., Wæhrens, E. E., Bliddal, H., Christensen, R., ... & Kristensen, L. E. (2021). Liraglutide after diet-induced weight loss for pain and weight control in knee osteoarthritis: a randomized controlled trial. *The American journal of clinical nutrition*, *113*(2), 314-323. - Marchesini, G., Natale, S., Chierici, S., Manini, R., Besteghi, L., Di Domizio, S., ... & Melchionda, N. (2002). Effects of cognitive–behavioural therapy on health-related quality of life in obese subjects with and without binge eating disorder. *International journal of obesity*, *26*(9), 1261-1267. - Roberts, C. R. (1978). Psychological treatment of obesity with phentermine resin as an adjunct. *The American Journal of Psychiatry*, *135*(8), 936-939. - Gilbert, M., Raman, J., & Sui, Z. (2021). Cognitive remediation-enabled cognitive behaviour therapy for obesity: a case series. *Eating and Weight Disorders-Studies on Anorexia, Bulimia and Obesity*, *26*, 103-114. - Keller, C. (1999). Commentary: Obese binge eating women had no weight loss with diet or non-diet therapies. *Evidence-Based Nursing*, *2*(1), 17. - Muggia, C., Falchi, A. G., Michelini, I., Montagna, E., De Silvestri, A., Grecchi, I., ... & Tinelli, C. (2014). Brief group cognitive behavioral treatment in addition to prescriptive diet versus standard care in obese and overweight patients. A randomized controlled trial. *e-SPEN Journal*, *9*(1), e26-e33. - Evans, B. C., Murray, H. B., Muratore, A. F., Lantz, E. L., & Juarascio, A. S. (2019). Developing an acceptance-based behavioral weight loss treatment for individuals with binge eating pathology: a preliminary proof of concept study and clinical case series. *Cognitive and behavioral practice*, *26*(2), 395-410. - Albassam, R. S., Abdel Gawwad, E. S., & Khanam, L. (2007). Weight management practices and their relationship to knowledge, perception and health status of Saudi females attending diet clinics in Riyadh city. *J Egypt Public Health Assoc*, *82*(1-2), 173-201. - Guisado-Macías, J. A., Méndez-Sánchez, F., Baltasar-Tello, I., Zamora-Rodríguez, F. J., Escudero-Sánchez, A. B., & Vaz-Leal, F. J. (2016). Fluoxetine, topiramate, and combination of both to stabilize eating behavior before bariatric surgery. *Actas Espanolas de Psiquiatria*, *44*(3), 93-96. - Gorin, A. A., Niemeier, H. M., Hogan, P., Coday, M., Davis, C., DiLillo, V. G., ... & Look AHEAD Research Group. (2008). Binge eating and weight loss outcomes in overweight and obese individuals with type 2 diabetes: results from the Look AHEAD trial. *Archives of general psychiatry*, *65*(12), 1447-1455. - Stahre, L., & Hällström, T. (2005). A short-term cognitive group treatment program gives substantial weight reduction up to 18 months from the end of treatment. A randomized controlled trial. *Eating and Weight Disorders-Studies on Anorexia, Bulimia and Obesity*, *10*, 51-58. - Promoting Lifestyle Change Via Tailored mHealth Feedback to Improve Health (SMARTER), ClinicalTrials.gov ID NCT03367936. Sponsor University of Pittsburgh, Information provided by Lora Burke, University of Pittsburgh (Responsible Party) - Legenbauer, T., Burgmer, R., Senf, W., & Herpertz, S. (2007). Psychiatric comorbidity and quality of life in obese individuals--a prospective controlled study. *Psychotherapie, Psychosomatik, medizinische Psychologie*, *57*(11), 435-441. - Controlled prospective study to evaluate the inpatient naturopathic fasting therapy in overweight and obese patients, Main ID: DRKS00006343, http://drks.de/search/en/trial/DRKS00006343 - Anton, S. D., Martin, C. K., Redman, L., York-Crowe, E., Heilbronn, L. K., Han, H., ... & Ravussin, E. (2008). Psychosocial and behavioral pre-treatment predictors of weight loss outcomes. *Eating and Weight Disorders-Studies on Anorexia, Bulimia and Obesity*, *13*, 30-37. - Sherwood, N. E., Jeffery, R. W., & Wing, R. R. (1999). Binge status as a predictor of weight loss treatment outcome. *International Journal of Obesity*, *23*(5), 485-493. - Buclin-Thiébaud, S., Pataky, Z., Bruchez, V., & Golay, A. (2010). New psycho-pedagogic approach to obesity treatment: a 5-year follow-up. *Patient education and counseling*, *79*(3), 333-337. - Pagoto, S., Bodenlos, J. S., Kantor, L., Gitkind, M., Curtin, C., & Ma, Y. (2007). Association of major depression and binge eating disorder with weight loss in a clinical setting. *Obesity*, *15*(11), 2557-2559. - Sasdelli, A. S., Petroni, M. L., Delli Paoli, A., Collini, G., Calugi, S., Dalle Grave, R., & Marchesini, G. (2018). Expected benefits and motivation to weight loss in relation to treatment outcomes in group-based cognitive-behavior therapy of obesity. *Eating and Weight Disorders-Studies on Anorexia, Bulimia and Obesity*, *23*, 205-214. - Jorge, R., Santos, I., Tomás, R., Silva, M. N., Carraça, E. V., Teixeira, V. H., & Teixeira, P. J. (2020). Behavioural and psychological pretreatment predictors of short-and long-term weight loss among women with overweight and obesity. *Eating and Weight Disorders-Studies on Anorexia, Bulimia and Obesity*, *25*, 1377-1385. - Imperatori, C., Innamorati, M., Lamis, D. A., Contardi, A., Continisio, M., Castelnuovo, G., ... & Fabbricatore, M. (2016). Factor structure of the binge eating scale in a large sample of obese and overweight patients attending low energy diet therapy. *European Eating Disorders Review*, *24*(2), 174-178. - Akalin, A., Yazici, F., & Erol, A. (2009). The relation of sibutramin efficacy with psycopathology and eating pathologies. *KLINIK PSIKOFARMAKOLOJI BULTENI-BULLETIN OF CLINICAL PSYCHOPHARMACOLOGY*, *19*. - Appolinario, J. C., & McElroy, S. L. (2004). Pharmacological approaches in the treatment of binge eating disorder. *Current Drug Targets*, *5*(3), 301-307. - Tur, J. J., Escudero, A. J., Romaguera, D., & Burguera, B. (2013). How can we predict which morbidly obese patients will adhere to weight-loss programs based on life style changes?. *Endocrinología y Nutrición*, *60*(6), 297-302. - Eichen, D. M., Matheson, B. E., Appleton-Knapp, S. L., & Boutelle, K. N. (2017). Neurocognitive treatments for eating disorders and obesity. *Current psychiatry reports*, *19*, 1-10. - Rostanzo, E., Marchetti, M., Casini, I., & Aloisi, A. M. (2021). Very-low-calorie ketogenic diet: A potential treatment for binge eating and food addiction symptoms in women. A pilot study. *International Journal of Environmental Research and Public Health*, *18*(23), 12802. - Legenbauer, T., Mueller, A., de Zwaan, M., Fischer, C., Burgmer, R., & Herpertz, S. (2018). The impact of self‐reported impulsivity on the course of weight is mediated by disinhibited eating. *European Eating Disorders Review*, *26*(1), 38-45. - Chao, A. M., Wadden, T. A., Gorin, A. A., Shaw Tronieri, J., Pearl, R. L., Bakizada, Z. M., ... & Berkowitz, R. I. (2017). Binge eating and weight loss outcomes in individuals with type 2 diabetes: 4‐year results from the Look AHEAD Study. *Obesity*, *25*(11), 1830-1837. - Barnes, R. D., Ivezaj, V., Pittman, B. P., & Grilo, C. M. (2018). Early weight loss predicts weight loss treatment response regardless of binge‐eating disorder status and pretreatment weight change. *International Journal of Eating Disorders*, *51*(6), 558-564. - Grilo, C. M. (2006). Cognitive behavioural therapy does not improve outcome in obese women with binge eating disorder receiving a comprehensive very low calorie diet programme. *Evidence-based mental health*, *9*(1), 12-12. - Balzer, K., Hesse, K., Eisold, U., & Kopke, S. (2013). Better health for body and soul. *Pflege Zeitschrift*, *66*(11), 652-655. - Sawamoto, R., Nozaki, T., Furukawa, T., Tanahashi, T., Morita, C., Hata, T., ... & Sudo, N. (2016). Predictors of dropout by female obese patients treated with a group cognitive behavioral therapy to promote weight loss. *Obesity facts*, *9*(1), 29-38. - Pacanowski, C. R., Linde, J. A., Faulconbridge, L. F., Coday, M., Safford, M. M., Chen, H., ... & Jeffery, R. W. (2018). Psychological status and weight variability over eight years: Results from Look AHEAD. *Health Psychology*, *37*(3), 238. - Dubbert, P. M., & Wilson, G. T. (1984). Goal-setting and spouse involvement in the treatment of obesity. *Behaviour Research and Therapy*, *22*(3), 227-242. - Delinsky, S. S., Latner, J. D., & Wilson, G. T. (2006). Binge eating and weight loss in a self‐help behavior modification program. *Obesity*, *14*(7), 1244-1249. - TEAIMA, M., HAMID, M. M. A., SHOMAN, N. A., JASTI, B. R., ELNABARAWI, M. A., & YASSER, M. (2021). BINGE EATING DISORDERS; UPDATED AND EMERGING APPROACHES. *Int J App Pharm*, *13*(2), 84-93. - Abrams, M. (1991). The eating disorder inventory as a predictor of compliance in a behavioral weight‐loss program. *International Journal of Eating Disorders*, *10*(3), 355-360. - Sysko, R., Michaelides, A., Costello, K., Herron, D. M., & Hildebrandt, T. (2022). An initial test of the efficacy of a digital health intervention for bariatric surgery candidates. *Obesity surgery*, *32*(11), 3641-3649. - Warschburger, P., Wortmann, H. R., Gisch, U. A., Baer, N. R., Schenk, L., Anton, V., & Bergmann, M. M. (2022). An experimental approach to training interoceptive sensitivity: study protocol for a pilot randomized controlled trial. *Nutrition Journal*, *21*(1), 74. - Grammer, A. C., Best, J. R., Fowler, L. A., Stein, R. I., Kolko Conlon, R. P., Balantekin, K. N., ... & Wilfley, D. E. (2023). Change in parent and child psychopathology following obesity treatment and maintenance: A secondary data analysis. *Pediatric obesity*, *18*(1), e12971. - Pala, B., Pennazzi, L., Tifi, P., Alivernini, M. C., Nardoianni, G., Barbato, E., & Tocci, G. (2023). Italian Society of Obesity XI National Congress. - Kolnikaj, T. S., Herman, R., Janež, A., & Jensterle, M. (2022). Assessment of eating disorders and eating behavior to improve treatment outcomes in women with polycystic ovary syndrome. *Life*, *12*(11), 1906. - Kalra, S., Bathla, M., & Kapoor, N. (2022). Baromania: A contrarian epidemic. *JPMA. The Journal of the Pakistan Medical Association*, *72*(12), 2567-2568. - Himmerich, H., Bentley, J., & McElroy, S. L. (2024). Pharmacological treatment of binge eating disorder and frequent comorbid diseases. *CNS drugs*, *38*(9), 697-718. - Mundstock, R. F., de Lima Silva, G. M., Titton, C. A., Kuskoski, D. G., & Busnello, F. M. (2023). Relationship between adherence to dietary treatment and binge-eating disorder in obese patients. *Nutrición Hospitalaria*, *40*(5). - Riccardo Dalle Grave, M. D. (2023). Obesity and eating disorders: an interactive and complex coexistence. | Ineligible study design |
| - Goode, R. W., Kalarchian, M. A., Conroy, M., Craighead, L., Sereika, S., Mattos, M., ... & Burke, L. E. (2017, March). Feasibility of an Appetite Awareness Intervention to Reduce Cardiovascular Disease Risk Factors and Binge Eating in African-American Women with Obesity. In *CIRCULATION* (Vol. 135). TWO COMMERCE SQ, 2001 MARKET ST, PHILADELPHIA, PA 19103 USA: LIPPINCOTT WILLIAMS & WILKINS. - Jospe, M. R., Brown, R. C., Williams, S. M., Roy, M., Meredith‐Jones, K. A., & Taylor, R. W. (2018). Self‐monitoring has no adverse effect on disordered eating in adults seeking treatment for obesity. *Obesity Science & Practice*, *4*(3), 283-288. - Corsica, J., Hood, M. M., Katterman, S., Kleinman, B., & Ivan, I. (2014). Development of a novel mindfulness and cognitive behavioral intervention for stress-eating: a comparative pilot study. *Eating behaviors*, *15*(4), 694-699. - Call, C. C., D'Adamo, L., Butryn, M. L., & Stice, E. (2021). Examining weight suppression as a predictor and moderator of intervention outcomes in an eating disorder and obesity prevention trial: A replication and extension study. *Behaviour research and therapy*, *141*, 103850. - Beintner, I., Emmerich, O. L. M., Vollert, B., Taylor, C. B., & Jacobi, C. (2019). Promoting positive body image and intuitive eating in women with overweight and obesity via an online intervention: Results from a pilot feasibility study. *Eating behaviors*, *34*, 101307. - Linardon, J. (2017). *The cognitive-behavioural theory and treatment for eating disorders and disordered eating: A direct evaluation* (Doctoral dissertation, Australian Catholic University). - Ricca, V., Castellini, G., Mannucci, E., Sauro, C. L., Ravaldi, C., Rotella, C. M., & Faravelli, C. (2010). Comparison of individual and group cognitive behavioral therapy for binge eating disorder. A randomized, three-year follow-up study. *Appetite*, *55*(3), 656-665. - Rahmani, M., Omidi, A., Asemi, Z., & Akbari, H. (2018). The effect of dialectical behaviour therapy on binge eating, difficulties in emotion regulation and BMI in overweight patients with binge-eating disorder: A randomized controlled trial. *Mental Health & Prevention*, *9*, 13-18. - Stepped Care for Binge Eating Disorder: Predicting Response to Minimal Intervention in a Randomized Controlled Trial, ClinicalTrials.gov ID NCT01837953, Sponsor: Ottawa Hospital Research Institute, Information provided by Ottawa Hospital Research Institute (Responsible Party) - Vanderlinden, J., Adriaensen, A., Vancampfort, D., Pieters, G., Probst, M., & Vansteelandt, K. (2012). A cognitive-behavioral therapeutic program for patients with obesity and binge eating disorder: short-and long-term follow-up data of a prospective study. *Behavior modification*, *36*(5), 670-686. - Torres, S., Sales, C. M., Guerra, M. P., Simões, M. P., Pinto, M., & Vieira, F. M. (2020). Emotion-focused cognitive behavioral therapy in comorbid obesity with binge eating disorder: A pilot study of feasibility and long-term outcomes. *Frontiers in Psychology*, *11*, 343. - Yu, Z., Roberts, B., Snyder, J., Stuart, K., Wilburn, J., Pudwill, H., & Cortazzo, K. (2021). A pilot study of a videoconferencing-based binge eating disorder program in overweight or obese females. *Telemedicine and e-Health*, *27*(3), 330-340. - Lammers, M. W., Vroling, M. S., Crosby, R. D., & van Strien, T. (2020). Dialectical behavior therapy adapted for binge eating compared to cognitive behavior therapy in obese adults with binge eating disorder: a controlled study. *Journal of eating disorders*, *8*, 1-11. - Lores, T., Musker, M., Collins, K., Burke, A., Perry, S. W., Wong, M. L., & Licinio, J. (2020). Pilot trial of a group cognitive behavioural therapy program for comorbid depression and obesity. *BMC psychology*, *8*, 1-11. - Chami, R., Reichenberger, J., Cardi, V., Lawrence, N., Treasure, J., & Blechert, J. (2021). Characterising binge eating over the course of a feasibility trial among individuals with binge eating disorder and bulimia nervosa. *Appetite*, *164*, 105248. - Fitzsimmons-Craft, E. E., Taylor, C. B., Graham, A. K., Sadeh-Sharvit, S., Balantekin, K. N., Eichen, D. M., ... & Wilfley, D. E. (2020). Effectiveness of a digital cognitive behavior therapy–guided self-help intervention for eating disorders in college women: A cluster randomized clinical trial. *JAMA network open*, *3*(8), e2015633-e2015633. - Abrahamsson, N., Ahlund, L., Ahrin, E., & Alfonsson, S. (2018). Video-based CBT-E improves eating patterns in obese patients with eating disorder: A single case multiple baseline study. *Journal of behavior therapy and experimental psychiatry*, *61*, 104-112. - Schag, K., Leehr, E. J., Martus, P., Bethge, W., Becker, S., Zipfel, S., & Giel, K. E. (2015). Impulsivity-focused group intervention to reduce binge eating episodes in patients with binge eating disorder: study protocol of the randomised controlled IMPULS trial. *BMJ open*, *5*(12), e009445. - Hilbert, A., Saelens, B. E., Stein, R. I., Mockus, D. S., Welch, R. R., Matt, G. E., & Wilfley, D. E. (2007). Pretreatment and process predictors of outcome in interpersonal and cognitive behavioral psychotherapy for binge eating disorder. *Journal of Consulting and Clinical Psychology*, *75*(4), 645. - Schag, K., Rennhak, S. K., Leehr, E. J., Skoda, E. M., Becker, S., Bethge, W., ... & Giel, K. E. (2019). IMPULS: impulsivity-focused group intervention to reduce binge eating episodes in patients with binge eating disorder–a randomised controlled trial. *Psychotherapy and psychosomatics*, *88*(3), 141-153. - Wagner, B., Nagl, M., Dölemeyer, R., Klinitzke, G., Steinig, J., Hilbert, A., & Kersting, A. (2016). Randomized controlled trial of an internet-based cognitive-behavioral treatment program for binge-eating disorder. *Behavior Therapy*, *47*(4), 500-514. - Eik-Nes, T. T., Vrabel, K., Raman, J., Clark, M. R., & Berg, K. H. (2021). A group intervention for individuals with obesity and comorbid binge eating disorder: Results from a feasibility study. *Frontiers in Endocrinology*, *12*, 738856. - Cachelin, F. M., Gil-Rivas, V., Palmer, B., Vela, A., Phimphasone, P., de Hernandez, B. U., & Tapp, H. (2019). Randomized controlled trial of a culturally-adapted program for Latinas with binge eating. *Psychological Services*, *16*(3), 504. - Cognolato, S., Silvestri, A., AL, F. B., & Santonastaso, P. (1996). Psychodynamic group psychotherapy with obese patients. *Minerva psichiatrica*, *37*(1), 5-12. - Mason, T. B., Smith, K. E., Williams-Kerver, G. A., Crosby, R. D., Engel, S. G., Crow, S. J., ... & Peterson, C. B. (2021). Descriptives and baseline ecological momentary assessed predictors of weight change over the course of psychological treatments for binge eating disorder. *Journal of psychosomatic research*, *143*, 110373. - Popkess-Vawter, S., & Owens, V. (1999). Use of the bulit bulimia screening questionnaire to assess risk and progress in weight management for overweight women who weight cycle. *Addictive behaviors*, *24*(4), 497-507. - Lewis, V. J., Blair, A. J., & Booth, D. A. (1992). Outcome of group therapy for body-image emotionality and weight-control self-efficacy. *Behavioural and Cognitive Psychotherapy*, *20*(2), 155-165. - Rahimi-Ardabili, H., Vartanian, L. R., Zwar, N., Sharpe, A., & Reynolds, R. C. (2020). Efficacy and acceptability of a pilot dietary intervention focusing on self-compassion, goal-setting and self-monitoring. *Public Health Nutrition*, *23*(15), 2746-2758. - de Zwaan, M., Herpertz, S., Zipfel, S., Tuschen-Caffier, B., Friederich, H. C., Schmidt, F., ... & Hilbert, A. (2012). INTERBED: internet-based guided self-help for overweight and obese patients with full or subsyndromal binge eating disorder. A multicenter randomized controlled trial. *Trials*, *13*, 1-13. - Yu, Z., Snyder, J., Stuart, K., Wilburn, J., Pudwill, H., Williams, B., & Cortazzo, K. (2017). A Web‐based Binge Eating Disorder Intervention Program Reduced Eating Disorder Risks in Overweight or Obese Females–A Pilot Study. *The FASEB Journal*, *31*, lb360-lb360. - Zervos, K., Koletsi, M., Mantzios, M., Skopeliti, N., Tsitsas, G., & Naska, A. (2022). An eight-week mindful eating program applied in a Mediterranean population with overweight or obesity: The EATT Intervention Study. *Psychological Reports*, *125*(2), 1011-1040. - Pinto‐Gouveia, J., Carvalho, S. A., Palmeira, L., Castilho, P., Duarte, C., Ferreira, C., ... & Costa, J. (2017). BEfree: A new psychological program for binge eating that integrates psychoeducation, mindfulness, and compassion. *Clinical psychology & psychotherapy*, *24*(5), 1090-1098. - Davis, C., Dutton, W. B., Durant, T., Annunziato, R. A., & Marcotte, D. (2014). Achieving cultural congruency in weight loss interventions: can a spirituality-based program attract and retain an inner-city community sample?. *Journal of Obesity*, *2014*. - Cancian, A. C. M., de Souza, L. A. S., Liboni, R. P. A., Machado, W. D. L., & Oliveira, M. D. S. (2019). Effects of a dialectical behavior therapy-based skills group intervention for obese individuals: A Brazilian pilot study. *Eating and Weight Disorders-Studies on Anorexia, Bulimia and Obesity*, *24*, 1099-1111. - Hoopes, S. P., Reimherr, F. W., Hedges, D. W., Rosenthal, N. R., Kamin, M., Karim, R., ... & Karvois, D. (2003). Treatment of bulimia nervosa with topiramate in a randomized, double-blind, placebo-controlled trial, part 1: improvement in binge and purge measures. *Journal of Clinical Psychiatry*, *64*(11), 1335-1341. - Levine, M. D., Marcus, M. D., & Moulton, P. (1996). Exercise in the treatment of binge eating disorder. *International Journal of Eating Disorders*, *19*(2), 171-177. - ter Huurne, E. D., Postel, M. G., de Haan, H. A., Drossaert, C. H., & DeJong, C. A. (2013). Web-based treatment program using intensive therapeutic contact for patients with eating disorders: before-after study. *Journal of Medical Internet Research*, *15*(2), e2211. - Pinto-Gouveia, J., Carvalho, S. A., Palmeira, L., Castilho, P., Duarte, C., Ferreira, C., ... & Costa, J. (2019). Incorporating psychoeducation, mindfulness and self-compassion in a new programme for binge eating (BEfree): Exploring processes of change. *Journal of health psychology*, *24*(4), 466-479. - Boutelle, K. N., Monreal, T., Strong, D. R., & Amir, N. (2016). An open trial evaluating an attention bias modification program for overweight adults who binge eat. *Journal of behavior therapy and experimental psychiatry*, *52*, 138-146. - Alfonsson, S., Parling, T., & Ghaderi, A. (2015). Group behavioral activation for patients with severe obesity and binge eating disorder: a randomized controlled trial. *Behavior Modification*, *39*(2), 270-294. - Sarto, H. M., Barcelo-Soler, A., Herrera-Mercadal, P., Pantilie, B., Navarro-Gil, M., Garcia-Campayo, J., & Montero-Marin, J. (2019). Efficacy of a mindful-eating programme to reduce emotional eating in patients suffering from overweight or obesity in primary care settings: a cluster-randomised trial protocol. *BMJ open*, *9*(11), e031327. - Beutel, M., Thiede, R., Wiltink, J., & Sobez, I. (2001). Effectiveness of behavioral and psychodynamic in-patient treatment of severe obesity—first results from a randomized study. *International Journal of Obesity*, *25*(1), S96-S98. - Souza, L. A. S. D., Cancian, A. C. M., Castro, T. G. D., & Oliveira, M. D. S. (2019). Problematic and adaptive eating in people with obesity after a DBT-based skills training intervention: 3-and 8-month follow-up and mediation analysis. *Psicologia: Reflexão e Crítica*, *32*, 1. - Wagner, B., Horn, A. B., & Maercker, A. (2014). Internet-based versus face-to-face cognitive-behavioral intervention for depression: a randomized controlled non-inferiority trial. *Journal of affective disorders*, *152*, 113-121. - König, H. H., Bleibler, F., Friederich, H. C., Herpertz, S., Lam, T., Mayr, A., ... & Egger, N. (2018). Economic evaluation of cognitive behavioral therapy and internet‐based guided self‐help for binge‐eating disorder. *International Journal of eating disorders*, *51*(2), 155-164. - Pendleton, V. R., Goodrick, G. K., Poston, W. S. C., Reeves, R. S., & Foreyt, J. P. (2002). Exercise augments the effects of cognitive‐behavioral therapy in the treatment of binge eating. *International Journal of Eating Disorders*, *31*(2), 172-184. - Seamoore, D., Buckroyd, J., & Stott, D. (2006). Changes in eating behaviour following group therapy for women who binge eat: a pilot study. *Journal of Psychiatric and Mental Health Nursing*, *13*(3), 337-346. - Ricca, V., Castellini, G., Mannucci, E., Sauro, C. L., Ravaldi, C., Rotella, C. M., & Faravelli, C. (2010). Comparison of individual and group cognitive behavioral therapy for binge eating disorder. A randomized, three-year follow-up study. *Appetite*, *55*(3), 656-665. - Goode, R. W., Kalarchian, M. A., Craighead, L., Conroy, M. B., Wallace Jr, J., Eack, S. M., & Burke, L. E. (2018). The feasibility of a binge eating intervention in Black women with obesity. *Eating Behaviors*, *29*, 83-90. - Alberts, H. J., Thewissen, R., & Raes, L. (2012). Dealing with problematic eating behaviour. The effects of a mindfulness-based intervention on eating behaviour, food cravings, dichotomous thinking and body image concern. *Appetite*, *58*(3), 847-851. - Emotion-focused therapy for binge-eating disorder: A pilot randomized control trial., ACTRN12620000563965, https://anzctr.org.au/ACTRN12620000563965.aspx - Malhotra, S., King, K. H., Welge, J. A., Brusman-Lovins, L., & McElroy, S. L. (2002). Venlafaxine treatment of binge-eating disorder associated with obesity: a series of 35 patients. *Journal of Clinical Psychiatry*, *63*(9), 802-806. - Paul, L., van der Heiden, C., van Hoeken, D., Deen, M., Vlijm, A., Klaassen, R. A., ... & Hoek, H. W. (2021). Cognitive behavioral therapy versus usual care before bariatric surgery: one-year follow-up results of a randomized controlled trial. *Obesity Surgery*, *31*, 970-979. - Pearl, R. L., Wadden, T. A., Hopkins, C. M., Shaw, J. A., Hayes, M. R., Bakizada, Z. M., ... & Alamuddin, N. (2017). Association between weight bias internalization and metabolic syndrome among treatment‐seeking individuals with obesity. *Obesity*, *25*(2), 317-322. - Kearney, D. J., Milton, M. L., Malte, C. A., McDermott, K. A., Martinez, M., & Simpson, T. L. (2012). Participation in mindfulness-based stress reduction is not associated with reductions in emotional eating or uncontrolled eating. *Nutrition Research*, *32*(6), 413-420. - Dastan, B., Afshar Zanjani, S., Froueddin Adl, A., & Habibi, M. (2020). The effectiveness of dialectical behaviour therapy for treating women with obesity suffering from BED: A feasibility and pilot study. *Clinical Psychologist*, *24*(2), 133-142. - Ricca, V., Castellini, G., Sauro, C. L., Rotella, C. M., & Faravelli, C. (2009). Zonisamide combined with cognitive behavioral therapy in binge eating disorder: A one-year follow-up study. *Psychiatry (Edgmont)*, *6*(11), 23. - Deumens, R. A., Noorthoorn, E. O., & Verbraak, M. J. (2012). Predictors for treatment outcome of binge eating with obesity: a naturalistic study. *Eating disorders*, *20*(4), 276-287. - Schyns, G., van den Akker, K., Roefs, A., Houben, K., & Jansen, A. (2020). Exposure therapy vs lifestyle intervention to reduce food cue reactivity and binge eating in obesity: A pilot study. *Journal of Behavior Therapy and Experimental Psychiatry*, *67*, 101453. - Gorin, A. A. (2000). *A controlled trial of cognitive-behavioral therapy with and without spousal involvement for binge eating disorder*. State University of New York at Stony Brook. - Roosen, M. A., Safer, D., Adler, S., Cebolla, A., & Van Strien, T. (2012). Group dialectical behavior therapy adapted for obese emotional eaters; a pilot study. *Nutricion hospitalaria*, *27*(4), 1141-1147. - Appolinario, J. C., Fontenelle, L. F., Papelbaum, M., Bueno, J. R., & Coutinho, W. (2002). Topiramate use in obese patients with binge eating disorder: an open study. *The Canadian Journal of Psychiatry*, *47*(3), 271-273. - Vela, A. M., Palmer, B., Gil-Rivas, V., & Cachelin, F. (2023). The role of disordered eating in type 2 diabetes: A pilot study. *American Journal of Lifestyle Medicine*, *17*(1), 131-139. - Evaluation of group psychotherapies to improve the self-regulation of overweight/obese people, <http://drks.de/search/en/trial/DRKS00005250> - Rasson, S. (2022, June). Reducing Eating disorders with a multidisciplinary intervention containing cognitive therapy, nutrition and physical exercise in overweighed adults between years 2016 and 2018. In *ANNALES MEDICO-PSYCHOLOGIQUES* (Vol. 180, No. 6, pp. 495-502). 21 STREET CAMILLE DESMOULINS, ISSY, 92789 MOULINEAUX CEDEX 9, FRANCE: MASSON EDITEUR. - Richard, K. (2017). *Treating Binge Eating Disorder with Eye Movement Desensitisation Reprocessing: A preliminary randomised controlled trial* (Doctoral dissertation). - Castillo, I., Solano, S., & Sepúlveda, A. R. (2019). A controlled study of an integrated prevention program for improving disordered eating and body image among Mexican university students: A 3‐month follow‐up. *European Eating Disorders Review*, *27*(5), 541-556. - Allen, H. N., & Craighead, L. W. (1999). Appetite monitoring in the treatment of binge eating disorder. *Behavior Therapy*, *30*(2), 253-272. - Boswell, R. G., Gueorguieva, R., & Grilo, C. M. (2023). Change in impulsivity is prospectively associated with treatment outcomes for binge-eating disorder. *Psychological medicine*, *53*(7), 2789-2797. - Grilo, C. M. (2014, April). PREDICTING MEANINGFUL OUTCOMES TO MEDICATION AND SELF-HELP TREATMENTS FOR BINGE EATING DISORDER IN PRIMARY CARE: THE SIGNIFICANCE OF RAPID RESPONSE. In *ANNALS OF BEHAVIORAL MEDICINE* (Vol. 47, pp. S231-S231). 233 SPRING ST, NEW YORK, NY 10013 USA: SPRINGER. - Leibbrand, R., & Fichter, M. M. (2002). Maintenance of weight loss after obesity treatment: is continuous support necessary?. *Behaviour research and therapy*, *40*(11), 1275-1289. - Leombruni, P., Pierò, A., Lavagnino, L., Brustolin, A., Campisi, S., & Fassino, S. (2008). A randomized, double-blind trial comparing sertraline and fluoxetine 6-month treatment in obese patients with Binge Eating Disorder. *Progress in Neuro-Psychopharmacology and Biological Psychiatry*, *32*(6), 1599-1605. - Berardi, K. L. (2008). The clinical effectiveness of cognitive behaviour therapy for the treatment of body image disturbance in women with eating disorders. - Cuneo, J. G., Godfrey, K. M., Wright, L. J., Backhaus, A., Miggantz, E., & Afari, N. (2018). Feasibility, acceptability, and exploratory outcomes of acceptance and commitment therapy for binge eating symptoms in veterans: a preliminary clinic-based study. *Journal of Cognitive Psychotherapy*, *32*(3), 155-170. - Elder, K. A. (2003). *Appetite-focused cognitive behavioral therapy for the early intervention of binge eating disorder*. University of Colorado at Boulder. - Carter, A. M. (2021). Compassion focused therapy for body weight shame. - Hildebrandt, T., Michaelides, A., Mackinnon, D., Greif, R., DeBar, L., & Sysko, R. (2017). Randomized controlled trial comparing smartphone assisted versus traditional guided self‐help for adults with binge eating. *International Journal of Eating Disorders*, *50*(11), 1313-1322. - Stice, E., Rohde, P., Gau, J. M., Butryn, M. L., Shaw, H., Cloud, K., & D'Adamo, L. (2021). Enhancing efficacy of a dissonance-based obesity and eating disorder prevention program: Experimental therapeutics. *Journal of consulting and clinical psychology*, *89*(10), 793. - Grilo, C. M., Crosby, R. D., Wilson, G. T., & Masheb, R. M. (2012). 12-month follow-up of fluoxetine and cognitive behavioral therapy for binge eating disorder. *Journal of consulting and clinical psychology*, *80*(6), 1108. - de Zwaan, M., Herpertz, S., Zipfel, S., Svaldi, J., Friederich, H. C., Schmidt, F., ... & Hilbert, A. (2017). Effect of internet-based guided self-help vs individual face-to-face treatment on full or subsyndromal binge eating disorder in overweight or obese patients: the INTERBED randomized clinical trial. *JAMA psychiatry*, *74*(10), 987-995. - The Effectiveness of Emotion-Focused Therapy On Anxiety, Depression and Difficulty in Emotion Regulation in Binge Eating Disorder, IRCT20200222046583N1, <http://en.irct.ir/trial/46071> - Ashton, K., Drerup, M., Windover, A., & Heinberg, L. (2009). Brief, four-session group CBT reduces binge eating behaviors among bariatric surgery candidates. *Surgery for Obesity and Related Diseases*, *5*(2), 257-262. Smith, D. E., Marcus, M. D., & Kaye, W. (1992). Cognitive‐behavioral treatment of obese binge eaters. *International Journal of Eating Disorders*, *12*(3), 257-262. - Traviss‐Turner, G. D., Philpot, U., Wilton, J., Green, K., Heywood‐Everett, S., & Hill, A. J. (2018). Guided self‐help to manage binge eating in a dietetic‐led community weight management service. *Clinical obesity*, *8*(4), 250-257. - Appolinario, J. C., Bacaltchuk, J., Sichieri, R., Claudino, A. M., Godoy-Matos, A., Morgan, C., ... & Coutinho, W. (2003). A randomized, double-blind, placebo-controlled study of sibutramine in the treatment of binge-eating disorder. *Archives of General Psychiatry*, *60*(11), 1109-1116. - Buckroyd, J., Rother, S., & Stott, D. (2006). Weight loss as a primary objective of therapeutic groups for obese women: two preliminary studies. *British Journal of Guidance & Counselling*, *34*(2), 245-265. - Kristeller, J. L. (2019). Mindfulness-Based Eating Awareness Training (MB-EAT). In *Handbook of Mindfulness-Based Programmes* (pp. 191-203). Routledge. - Dicker, S. L., & Craighead, L. W. (2004). Appetite-focused cognitive-behavioral therapy in the treatment of binge eating with purging. *Cognitive and Behavioral Practice*, *11*(2), 213-221. - Shelley-Ummenhofer, J., & MacMillan, P. D. (2007). Cognitive-behavioural treatment for women who binge eat. *Canadian Journal of Dietetic Practice and Research*, *68*(3), 139-142. - Crow, S. J. (2002). Group interpersonal psychotherapy may be as effective as group cognitive behavioural therapy for overweight people with binge eating disorder. *Psychiatry*, *59*, 713-21. - Gorin, A. A., Gokee LaRose, J., Espeland, M. A., Tate, D. F., Jelalian, E., Robichaud, E., ... & Wing, R. R. (2019). Eating pathology and psychological outcomes in young adults in self-regulation interventions using daily self-weighing. *Health Psychology*, *38*(2), 143. - Czepczor-Bernat, K., Brytek-Matera, A., & Staniszewska, A. (2021). The effect of a web-based psychoeducation on emotional functioning, eating behaviors, and body image among premenopausal women with excess body weight. *Archives of Women's Mental Health*, *24*, 423-435. - Wolff, G. E., & Clark, M. M. (2001). Changes in eating self-efficacy and body image following cognitive–behavioral group therapy for binge eating disorder: A clinical study. *Eating Behaviors*, *2*(2), 97-104. - Pendleton, V. R., Goodrick, G. K., Poston, W. S. C., Reeves, R. S., & Foreyt, J. P. (2002). Exercise augments the effects of cognitive‐behavioral therapy in the treatment of binge eating. *International Journal of Eating Disorders*, *31*(2), 172-184. - Castillo, I., Solano, S., & Sepulveda, A. R. (2016). Prevention program for disordered eating and obesity among Mexican university students. *BEHAVIORAL PSYCHOLOGY-PSICOLOGIA CONDUCTUAL*, *24*(1), 5-28. - Mortezaei Shemirani, S., Sanaei Zaker, B., Tajeri, B., Sodagar, S., & Meschi, F. (2020). Comparing the effects of acceptance and commitment group therapy and cognitive-behavioral group therapy on life habits, disordered eating behavior, and health-promoting lifestyle in obese women. *Journal of Arak University of Medical Sciences*, *23*(6), 944-957. - Evaluation of a smartphone-based stress reduction intervention for reducing episodes of disordered eating among women who engage in stress-related eating behaviours, U1111-1229-4886, https://anzctr.org.au/Trial/Registration/TrialReview.aspx?ACTRN=12619000432112 - van Riel, L., van den Berg, E., Polak, M., Geerts, M., Peen, J., Ingenhoven, T., & Dekker, J. (2023). Exploring effectiveness of CBT in obese patients with binge eating disorder: personality functioning is associated with clinically significant change. *BMC psychiatry*, *23*(1), 136. - Lampe, E. W., Srivastava, P., Presseller, E. K., Wilkinson, M. L., Trainor, C., Manasse, S. M., & Juarascio, A. S. (2024). Latent Change Trajectories in Mood During Focused CBT Enhanced for Eating Disorders Are Associated With Global Eating Pathology at Posttreatment and Follow-Up Among Individuals With Bulimia Nervosa Spectrum Disorders: A Preliminary Examination. *Behavior Therapy*. | No weight loss component |
| - Zamorano, A. S., Wilson, E. M., Liu, J., Leon, A., Kuroki, L. M., Thaker, P. H., ... & Hagemann, A. R. (2021). Text-message-based behavioral weight loss for endometrial cancer survivors with obesity: A randomized controlled trial. *Gynecologic oncology*, *162*(3), 770-777. - Piya, M. K., Chimoriya, R., Yu, W., Grudzinskas, K., Myint, K. P., Skelsey, K., ... & Hay, P. (2021). Improvement in eating disorder risk and psychological health in people with class 3 obesity: effects of a multidisciplinary weight management program. *Nutrients*, *13*(5), 1425. - Usubini, G., & Riboni, V. ACTonFood. Acceptance and Commitment Therapy-Based Group Treatment Compared to Cognitive Behavioral Therapy-Based Group Treatment for Weight Loss Maintenance: An Individually Randomized Group Treatment Trial. - Annesi, J. J., & Mareno, N. (2015). Improvement in emotional eating associated with an enhanced body image in obese women: mediation by weight‐management treatments' effects on self‐efficacy to resist emotional cues to eating. *Journal of advanced nursing*, *71*(12), 2923-2935. - Annesi, J. J., & Porter, K. J. (2015). Reciprocal effects of exercise and nutrition treatment-induced weight loss with improved body image and physical self-concept. *Behavioral Medicine*, *41*(1), 18-24. - Bick, D., Taylor, C., Avery, A., Bhavnani, V., Craig, V., Healey, A., ... & Ussher, M. (2019). Protocol for a two-arm feasibility RCT to support postnatal maternal weight management and positive lifestyle behaviour in women from an ethnically diverse inner city population: the SWAN feasibility trial. *Pilot and feasibility studies*, *5*, 1-12. - MacKean, S. S., Eskandari, H., Borjali, A., & Ghodsi, D. (2010). The comparison between efficacy of narrative therapy and diet therapy on body image in women with overweight and obesity. *Pajoohandeh Journal*, *15*(5), 225-232. - Černelič-Bizjak, M. (2019). Changes in body image during a 6-month lifestyle behaviour intervention in a sample of overweight and obese individuals. *Journal of Bodywork and Movement Therapies*, *23*(3), 515-520. - Joshi, P., Quintiliani, L., Bourland, A. C., Cuellar, A., Mahesri, M., Sullivan, L., & Apovian, C. (2016, May). BEHAVIORAL WEIGHT MANAGEMENT INTERVENTION IN UNDERSERVED OVERWEIGHT POSTPARTUM WOMEN, A RANDOMIZED CONTROLLED FEASIBILITY TRIAL: THE RENEW STUDY. In *JOURNAL OF GENERAL INTERNAL MEDICINE* (Vol. 31, pp. S137-S138). 233 SPRING ST, NEW YORK, NY 10013 USA: SPRINGER. - Optimizing Weight Loss Outcomes Through Body Image Enhancement, ClinicalTrials.gov ID NCT05090293, Sponsor: Texas Tech University, Information provided by Martin Binks - van Beurden, S. B., Smith, J. R., Lawrence, N. S., Abraham, C., & Greaves, C. J. (2019). Feasibility randomized controlled trial of ImpulsePal: smartphone app–based weight management intervention to reduce impulsive eating in overweight adults. *JMIR formative research*, *3*(2), e11586. - Riva, G., Bacchetta, M., Cesa, G., Conti, S., & Molinari, E. (2003). Six-month follow-up of in-patient experiential cognitive therapy for binge eating disorders. *Cyberpsychology & behavior*, *6*(3), 251-258. - Werlinger, K., King, T. K., Clark, M. M., Pera, V., & Wincze, J. P. (1997). Perceived changes in sexual functioning and body image following weight loss in an obese female population: A pilot study. *Journal of Sex & Marital Therapy*, *23*(1), 74-78. - Linde, J. A. (2014). A randomised pilot and feasibility study examining body weight tracking frequency and psychosocial health indicators. *Obesity Research & Clinical Practice*, *8*(4), e399-e402. - Annesi, J. J. (2007). Relations of changes in exercise self-efficacy, physical self-concept, and body satisfaction with weight changes in obese white and African American women initiating a physical activity program. *Ethnicity & Disease*, *17*(1), 19-22. - Rock, C. L., Pande, C., Flatt, S. W., Ying, C., Pakiz, B., Parker, B. A., ... & Nichols, J. F. (2013). Favorable changes in serum estrogens and other biologic factors after weight loss in breast cancer survivors who are overweight or obese. *Clinical breast cancer*, *13*(3), 188-195. - Pearl, R. L., Wadden, T. A., Chao, A. M., Walsh, O., Alamuddin, N., Berkowitz, R. I., & Tronieri, J. S. (2019). Weight bias internalization and long-term weight loss in patients with obesity. *Annals of Behavioral Medicine*, *53*(8), 782-787. - Katterman, S. N., Butryn, M. L., Hood, M. M., & Lowe, M. R. (2016). Daily weight monitoring as a method of weight gain prevention in healthy weight and overweight young adult women. *Journal of Health Psychology*, *21*(12), 2955-2965. - Annesi, J. J. (2009). Correlations of changes in weight and body satisfaction for obese women initiating exercise: assessing effects of ethnicity. *Psychological reports*, *105*(3_suppl), 1072-1076. - Annesi, J. J. (2017). Mediation of the relationship of behavioural treatment type and changes in psychological predictors of healthy eating by body satisfaction changes in women with obesity. *Obesity Research & Clinical Practice*, *11*(1), 97-107. - Durso, L. E. (2011). *The relationship of internalized weight bias to weight change in treatment-seeking overweight adults* (Doctoral dissertation, [Honolulu]:[University of Hawaii at Manoa],[December 2011]). - Kuzmar, I., Rizo, M., & Cortés-Castell, E. (2014). Adherence to an overweight and obesity treatment: how to motivate a patient?. *PeerJ*, *2*, e495. - Annesi, J. J., & Whitaker, A. C. (2010). Psychological factors discriminating between successful and unsuccessful weight loss in a behavioral exercise and nutrition education treatment. *International journal of behavioral medicine*, *17*, 168-175. - Foster, G. D., Wadden, T. A., & Vogt, R. A. (1997). Body image in obese women before, during, and after weight loss treatment. *Health Psychology*, *16*(3), 226. - Annesi, J. J. (2000). Effects of minimal exercise and cognitive behavior modification on adherence, emotion change, self-image, and physical change in obese women. *Perceptual and Motor Skills*, *91*(1), 322-336. - Vanderlinden, J., Adriaensen, A., Vancampfort, D., Pieters, G., Probst, M., & Vansteelandt, K. (2012). A cognitive-behavioral therapeutic program for patients with obesity and binge eating disorder: short-and long-term follow-up data of a prospective study. *Behavior modification*, *36*(5), 670-686. - Palmeira, A. L., Markland, D. A., Silva, M. N., Branco, T. L., Martins, S. C., Minderico, C. S., ... & Teixeira, P. J. (2009). Reciprocal effects among changes in weight, body image, and other psychological factors during behavioral obesity treatment: a mediation analysis. *International Journal of Behavioral Nutrition and Physical Activity*, *6*, 1-12. - Latner, J. D., Ciao, A. C., Wendicke, A. U., Murakami, J. M., & Durso, L. E. (2013). Community-based behavioral weight-loss treatment: Long-term maintenance of weight loss, physiological, and psychological outcomes. *Behaviour research and therapy*, *51*(8), 451-459. - Bick, D., Taylor, C., Avery, A., Bhavnani, V., Craig, V., Healey, A., ... & Ussher, M. (2019). Protocol for a two-arm feasibility RCT to support postnatal maternal weight management and positive lifestyle behaviour in women from an ethnically diverse inner city population: the SWAN feasibility trial. *Pilot and feasibility studies*, *5*, 1-12. - Dimitrov Ulian, M., Pinto, A. J., de Morais Sato, P., B. Benatti, F., Lopes de Campos-Ferraz, P., Coelho, D., ... & B. Scagliusi, F. (2018). Effects of a new intervention based on the Health at Every Size approach for the management of obesity: The “Health and Wellness in Obesity” study. *PLoS One*, *13*(7), e0198401. - Annesi, J. J., & Unruh, J. L. (2008). Relations of exercise, self-appraisal, mood changes and weight loss in obese women: testing propositions based on Baker and Brownell’s (2000) model. *The American journal of the medical sciences*, *335*(3), 198-204. - Stewart, T. M., Bachand, A. R., Han, H., Ryan, D. H., Bray, G. A., & Williamson, D. A. (2011). Body image changes associated with participation in an intensive lifestyle weight loss intervention. *Obesity*, *19*(6), 1290-1295. - Effects of Fasting Mimicking Diet (FMD) in Women With Polycystic Ovary Syndrome (PCOS), ClinicalTrials.gov ID NCT05196568, Sponsor: University of Salento, Information provided by Anna Maria Giudetti, University of Salento (Responsible Party) - The Effectiveness of Reality Therapy Based on Choice Theory along with   diet Therapy on Biochemical Indicators in Obese People: The Mediating  Role of Bipolar Thinking and Body Image, IRCT registration number: IRCT20190822044580N1, Registration date: 2020-03-19, 1398/12/29, Registration timing: retrospective   - Palmeira, A. L., Branco, T. L., Martins, S. C., Minderico, C. S., Silva, M. N., Vieira, P. N., ... & Teixeira, P. J. (2010). Change in body image and psychological well-being during behavioral obesity treatment: Associations with weight loss and maintenance. *Body Image*, *7*(3), 187-193. - Pearl, R. L., Wadden, T. A., Bach, C., Gruber, K., Leonard, S., Walsh, O. A., ... & Berkowitz, R. I. (2020). Effects of a cognitive-behavioral intervention targeting weight stigma: A randomized controlled trial. *Journal of Consulting and Clinical Psychology*, *88*(5), 470. - McCrea, C. (1995). An investigation of the usefulness of videofeedback in the treatment of obesity. *Clinical Psychology & Psychotherapy*, *2*(3), 192-198. - Downe, K. A., Goldfein, J. A., & Devlin, M. J. (2009). Restraint, hunger, and disinhibition following treatment for binge‐eating disorder. *International Journal of Eating Disorders*, *42*(6), 498-504. - Cash, T. F. (1994). Body image and weight changes in a multisite comprehensive very-low-calorie diet program. *Behavior Therapy*, *25*(2), 239-254. - Annesi, J. J., Mareno, N., & McEwen, K. (2016). Psychosocial predictors of emotional eating and their weight-loss treatment-induced changes in women with obesity. *Eating and Weight Disorders-Studies on Anorexia, Bulimia and Obesity*, *21*, 289-295. - Ames, G. E. (2005). *Reformulated cognitive behavioral treatment for obesity: A randomized pilot study investigating changes in expectations for treatment outcome*. University of Florida. - Bode, B. W., Testa, M. A., Magwire, M., Hale, P. M., Hammer, M., Blonde, L., ... & LEAD‐3 Study Group. (2010). Patient‐reported outcomes following treatment with the human GLP‐1 analogue liraglutide or glimepiride in monotherapy: results from a randomized controlled trial in patients with type 2 diabetes. *Diabetes, Obesity and Metabolism*, *12*(7), 604-612. - Leon, G. R. (1975). Personality, body image, and eating pattern changes in overweight persons after weight loss. *Journal of Clinical Psychology*, *31*(4), 618-623. - Male, D., Fergus, K., & Yufe, S. (2022). ‘Weighing’losses and gains: evaluation of the Healthy Lifestyle Modification After Breast Cancer Pilot Program. *Frontiers in Psychology*, *13*, 814671. - Riva, G., Bacchetta, M., Baruffi, M., & Molinari, E. (2002). Virtual-reality-based multidimensional therapy for the treatment of body image disturbances in binge eating disorders: a preliminary controlled study. *IEEE Transactions on Information Technology in Biomedicine*, *6*(3), 224-234. - Yarborough, B. J. H., Leo, M. C., Yarborough, M. T., Stumbo, S., Janoff, S. L., Perrin, N. A., & Green, C. A. (2016). Improvement in body image, perceived health, and health-related self-efficacy among people with serious mental illness: the STRIDE study. *Psychiatric Services*, *67*(3), 296-301. - Cooper, Z., Doll, H. A., Hawker, D. M., Byrne, S., Bonner, G., Eeley, E., ... & Fairburn, C. G. (2010). Testing a new cognitive behavioural treatment for obesity: A randomized controlled trial with three-year follow-up. *Behaviour research and therapy*, *48*(8), 706-713. - Crerand, C. E., Wadden, T. A., Foster, G. D., Sarwer, D. B., Paster, L. M., & Berkowitz, R. I. (2007). Changes in obesity‐related attitudes in women seeking weight reduction. *Obesity*, *15*(3), 740-747. - Lawlor, E. R., Islam, N., Bates, S., Griffin, S. J., Hill, A. J., Hughes, C. A., ... & Ahern, A. L. (2020). Third‐wave cognitive behaviour therapies for weight management: a systematic review and network meta‐analysis. *Obesity Reviews*, *21*(7), e13013. - Foster, G. D., Borradaile, K. E., Vander Veur, S. S., Shantz, K. L., Dilks, R. J., Goldbacher, E. M., ... & Satz, W. (2009). The effects of a commercially available weight loss program among obese patients with type 2 diabetes: a randomized study. *Postgraduate Medicine*, *121*(5), 113-118. - Rickman, A. D., Goodpaster, B., & Jakicic, J. (2013, May). The Effect Of Diet Versus Diet Plus Exercise On Body Image In Severely Obese. In *MEDICINE AND SCIENCE IN SPORTS AND EXERCISE* (Vol. 45, No. 5, pp. 709-709). 530 WALNUT ST, PHILADELPHIA, PA 19106-3621 USA: LIPPINCOTT WILLIAMS & WILKINS. - Cheruka, C. A., & Jakicic, J. M. (2021). Change In Fitness Domains Of Body Image In Response To A Behavioral Weight Loss Intervention: 889. *Medicine & Science in Sports & Exercise*, *53*(8S), 294. - Mostajabi, S. Z., Shoorab, N. J., Kordi, M., & Esmaily, H. (2022). The Effect of Training Based on Extended Parallel Process Model on Weight of Women with High Body Mass Index: A Cluster Randomized Trial. *Journal of Midwifery & Reproductive Health*, *10*(4). - Carraca, E., Silva, M., Vieira, P., Coutinho, S., Castro, M., Minderico, C. S., & Teixeira, P. (2008, October). Associations of Body Image with Obesity Level and With 12-Month Weight Change During a 12-Month Behavioral Weight Management Program in Women. In *OBESITY* (Vol. 16, pp. S159-S159). 75 VARICK ST, 9TH FLR, NEW YORK, NY 10013-1917 USA: NATURE PUBLISHING GROUP. - Raymond, N. C., de Zwaan, M., Mitchell, J. E., Ackard, D., & Thuras, P. (2002). Effect of a very low calorie diet on the diagnostic category of individuals with binge eating disorder. *International Journal of Eating Disorders*, *31*(1), 49-56. - Collings, A. S., Saules, K. K., & Saad, L. R. (2008). A prospective study of predictors of successful weight maintenance by women enrolled in community-based weight-loss programs. *Eating and Weight Disorders-Studies on Anorexia, Bulimia and Obesity*, *13*, 38-47. - Manzoni, G. M., Cesa, G. L., Bacchetta, M., Castelnuovo, G., Conti, S., Gaggioli, A., ... & Riva, G. (2016). Virtual reality–enhanced cognitive–behavioral therapy for morbid obesity: a randomized controlled study with 1 year follow-up. *Cyberpsychology, Behavior, and Social Networking*, *19*(2), 134-140. - Cesa, G. L., Manzoni, G. M., Bacchetta, M., Castelnuovo, G., Conti, S., Gaggioli, A., ... & Riva, G. (2013). Virtual reality for enhancing the cognitive behavioral treatment of obesity with binge eating disorder: randomized controlled study with one-year follow-up. *Journal of medical Internet research*, *15*(6), e113. - Stewart, T. M., Bachand, A. R., Han, H., Ryan, D. H., Bray, G. A., & Williamson, D. A. (2011). Body image changes associated with participation in an intensive lifestyle weight loss intervention. *Obesity*, *19*(6), 1290-1295. - Scott, S. E., Duarte, C., Encantado, J., Evans, E. H., Harjumaa, M., Heitmann, B. L., ... & Stubbs, R. J. (2019). The NoHoW protocol: a multicentre 2× 2 factorial randomised controlled trial investigating an evidence-based digital toolkit for weight loss maintenance in European adults. *BMJ open*, *9*(9), e029425. - Annesi, J. J., & Marti, C. N. (2011). Path analysis of exercise treatment-induced changes in psychological factors leading to weight loss. *Psychology & Health*, *26*(8), 1081-1098. - Ribeiro, A. A., de Lima Oliveira, V. T., & Becerra, R. A. (2020). Behavioral and eating disorders in obese patients after intervention group/ALTERACOES COMPORTAMENTAIS E ALIMENTARES EM OBESOS APOS GRUPO DE INTERVENCAO. *Revista Brasileira de Obesidade, Nutrição e Emagrecimento*, *14*(87), 641-652. - Sohrabi, F., Pasha, R., Naderi, F., Askary, P., & Ehteshamzadeh, P. (2018). Effectiveness of cognitive-behavioral therapy on Body Mass Index and self-concept perceptions of overweight individuals. *Iranian Journal of Nutrition Sciences and Food Technology*, *12*(4), 43-51. - Olthof, I., van den Berg, E. M., Boom, Y., Peen, J., & Dekker, J. (2010). Effect study of multidisciplinary group-treatment of morbid obesity in a clinic for eating disorders. *PSYCHOLOGIE & GEZONDHEID*, *38*(2), 57-65. - Petelin, A., Bizjak, M., Černelič-Bizjak, M., Jurdana, M., Jakus, T., & Jenko-Pražnikar, Z. (2014). Low-grade inflammation in overweight and obese adults is affected by weight loss program. *Journal of endocrinological investigation*, *37*, 745-755. - Castillo-Hernández, I. M., Vishwanathan, M., & Evans, E. M. (2019). Effects of a Weight Management Intervention on Holiday Weight Change And Body Image in Inactive Overweight Midlife Postmenopausal Women: 3064 Board# 110 May 31 3: 30 PM-5: 00 PM. *Medicine & Science in Sports & Exercise*, (6S), 844-845. - ALI, L. A. E. H., KADER, N. M. A., & MAHGOUB, N. A. (2021). Dietary/Exercise Guiding Program for Improvement of Nursing Students Body Image, Self-Esteem and Overweight Prevention: A Randomized Controlled study. *Pak J Med Sci*, *15*(1), 388-392. - Ginis, K. A. M., McEwan, D., Josse, A. R., & Phillips, S. M. (2012). Body image change in obese and overweight women enrolled in a weight-loss intervention: The importance of perceived versus actual physical changes. *Body image*, *9*(3), 311-317. - Annesi, J. J. (2021). Effects of increased exercise on propensity for emotional eating through associated psychological changes. *Journal of Nutrition Education and Behavior*, *53*(11), 944-950. - Pataky, Z., Gasteyger, C., Ziegler, O., Rissanen, A., Hanotin, C., & Golay, A. (2013). Efficacy of rimonabant in obese patients with binge eating disorder. *Experimental and Clinical Endocrinology & Diabetes*, *121*(01), 20-26. - Chad-Friedman, E., Pearsall, M., Miller, K. M., Wheeler, A. E., Denninger, J. W., Mehta, D. H., & Dossett, M. L. (2018). Total lifestyle coaching: A pilot study evaluating the effectiveness of a mind–body and nutrition telephone coaching program for obese adults at a community health center. *Global Advances in Health and Medicine*, *7*, 2164956118784902. - Halseth, A., Shan, K., Gilder, K., Malone, M., Acevedo, L., & Fujioka, K. (2018). Quality of life, binge eating and sexual function in participants treated for obesity with sustained release naltrexone/bupropion. *Obesity Science & Practice*, *4*(2), 141-152. - Wadden, T. A., Foster, G. D., & Letizia, K. A. (1994). One-year behavioral treatment of obesity: comparison of moderate and severe caloric restriction and the effects of weight maintenance therapy. *Journal of consulting and clinical psychology*, *62*(1), 165. - Reas, D. L. (2002). *Relationship between weight loss and body image in obese individuals seeking weight loss treatment*. Louisiana State University and Agricultural & Mechanical College. - Tseng, M. C., Lee, Y. J., Chen, S. Y., Lee, M. B., Lin, K. H., Chen, P. R., & Lai, J. S. (2002). Psychobehavioral response and weight loss prediction in a hospital-based weight reduction program. *Journal of the Formosan Medical Association*, *101*(10), 705-711. - Yarborough, B. J. H., Leo, M. C., Yarborough, M. T., Stumbo, S., Janoff, S. L., Perrin, N. A., & Green, C. A. (2016). Improvement in body image, perceived health, and health-related self-efficacy among people with serious mental illness: the STRIDE study. *Psychiatric Services*, *67*(3), 296-301. - Carraca, E. V., Markland, D., Silva, M. N., Coutinho, S. R., Vieira, P. N., Minderico, C. S., ... & Teixeira, P. J. (2012). Physical activity predicts changes in body image during obesity treatment in women. *Medicine & Science in Sports & Exercise*, *44*(8), 1604-1612. - Alici, M., & Pinar, R. (2008). Eveluation [sic] of the effectiveness of education given to obese patients. *Turkish Journal of Research & Development in Nursing/Hemşirelikte Araştırma Geliştirme Dergisi*, *10*(2). - Roughan, P., Seddon, E., & Vernon-Roberts, J. (1990). Long-term effects of a psychologically based group programme for women preoccupied with body weight and eating behaviour. *International Journal of Obesity*, *14*(2), 135-147. - Parretti, H. M., Ives, N. J., Tearne, S., Vince, A., Greenfield, S. M., Jolly, K., ... & Daley, A. (2020). Protocol for the feasibility and acceptability of a brief routine weight management intervention for postnatal women embedded within the national child immunisation programme: randomised controlled cluster feasibility trial with nested qualitative study (PIMMS-WL). *BMJ open*, *10*(2), e033027. - Korneeva, E. V. (2015). LONG-TERM EFFECTS OF ORLISTAT ON EATING BEHAVIOR OF OBESITY PATIENTS. *Russian Journal of Cardiology*, (9), 65-70. - Annesi, J. J. (2017). Physical activity in the treatment of obesity: A marker of psychosocial predictors of controlled eating, or facilitator of their improvements in women with differing body images. *Minerva Psichiatrica*. - Teixeira, P. J., Palmeira, A. L., Branco, T. L., Martins, S. S., Minderico, C. S., Barata, J. T., ... & Sardinha, L. B. (2004). Who will lose weight? A reexamination of predictors of weight loss in women. *International Journal of Behavioral Nutrition and Physical Activity*, *1*, 1-12. - Annesi, J. J. (2010). Relations of changes in self-regulatory efficacy and physical self-concept with improvements in body satisfaction in obese women initiating exercise with cognitive-behavioral support. *Body Image*, *7*(4), 356-359. - Palmeira, L., Pinto-Gouveia, J., & Cunha, M. (2017). Exploring the efficacy of an acceptance, mindfulness & compassionate-based group intervention for women struggling with their weight (Kg-Free): A randomized controlled trial. *Appetite*, *112*, 107-116. - Joseph, G., Arviv-Eliashiv, R., & Tesler, R. (2020). A comparison of diet versus diet+ exercise programs for health improvement in middle-aged overweight women. *Women's Health*, *16*, 1745506520932372. - Klassen, A. F., Cano, S. J., Alderman, A., Soldin, M., Thoma, A., Robson, S., ... & Pusic, A. L. (2016). The BODY-Q: a patient-reported outcome instrument for weight loss and body contouring treatments. *Plastic and Reconstructive Surgery–Global Open*, *4*(4), e679. - Meekums, B., Vaverniece, I., Majore-Dusele, I., & Rasnacs, O. (2012). Dance movement therapy for obese women with emotional eating: A controlled pilot study. *The Arts in Psychotherapy*, *39*(2), 126-133. - Annesi, J. J., & Gorjala, S. (2010). Body satisfaction and overall mood: Effects of race in exercisers with obesity. *Social Behavior and Personality: an international journal*, *38*(8), 1105-1109. - Goodpaster, B. H., DeLany, J. P., Otto, A. D., Kuller, L., Vockley, J., South-Paul, J. E., ... & Jakicic, J. M. (2010). Effects of diet and physical activity interventions on weight loss and cardiometabolic risk factors in severely obese adults: a randomized trial. *Jama*, *304*(16), 1795-1802. - Patrick, K., Marshall, S. J., Davila, E. P., Kolodziejczyk, J. K., Fowler, J. H., Calfas, K. J., ... & Robinson, T. N. (2014). Design and implementation of a randomized controlled social and mobile weight loss trial for young adults (project SMART). *Contemporary clinical trials*, *37*(1), 10-18. - Slaba, S. (2004). The Life Quality Change in Obese Patients after Short-time Cognitive Behavioral Therapy Intervention. *CESKA A SLOVENSKA PSYCHIATRIE*, *100*(6), 343-347. - Willis, E. D., McCoy, B., & Berman, M. (1990, April). THE EFFECT OF A WEIGHT MANAGEMENT PROGRAM ON SELF-ESTEEM AND BODY-IMAGE IN OBESE YOUTH. In *American Journal of Diseases of Children* (Vol. 144, No. 4, pp. 417-417). 515 N STATE ST, CHICAGO, IL 60610: AMER MEDICAL ASSOC. - Shapiro, J. R., Koro, T., Doran, N., Thompson, S., Sallis, J. F., Calfas, K., & Patrick, K. (2012). Text4Diet: a randomized controlled study using text messaging for weight loss behaviors. *Preventive medicine*, *55*(5), 412-417. - Compare, A., & Tasca, G. A. (2016). The rate and shape of change in binge eating episodes and weight: An effectiveness trial of emotionally focused group therapy for binge‐eating disorder. *Clinical psychology & psychotherapy*, *23*(1), 24-34. - Ames, G. E., Perri, M. G., Fox, L. D., Fallon, E. A., De Braganza, N., Murawski, M. E., ... & Hausenblas, H. A. (2005). Changing weight-loss expectations: a randomized pilot study. *Eating behaviors*, *6*(3), 259-269. - Webber, K. H., Casey, E. M., Mayes, L., Katsumata, Y., & Mellin, L. (2016). A comparison of a behavioral weight loss program to a stress management program: A pilot randomized controlled trial. *Nutrition*, *32*(7-8), 904-909. - Bick, D., Taylor, C., Avery, A., Bhavnani, V., Craig, V., Healey, A., ... & Ussher, M. (2019). Protocol for a two-arm feasibility RCT to support postnatal maternal weight management and positive lifestyle behaviour in women from an ethnically diverse inner city population: the SWAN feasibility trial. *Pilot and feasibility studies*, *5*, 1-12. - Jiskoot, L. G., de Niet, J. E., van Busschbach, J. J., van Stigt, V. W. H. C., van Egmond, A. M. E., Verbiest, A. C. M., ... & Laven, J. S. E. (2012, January). x00EC; Losing weight, gaining fertilityi: a multidisciplinary lifestyle program for overweight women with polycystic ovary syndrome (PCOS), a study protocol. In *HUMAN REPRODUCTION* (Vol. 27). GREAT CLARENDON ST, OXFORD OX2 6DP, ENGLAND: OXFORD UNIV PRESS. - Jiskoot, L. G., de Niet, J. E., van Busschbach, J. J., van Stigt, V. W. H. C., van Egmond, A. M. E., Verbiest, A. C. M., ... & Laven, J. S. E. (2012, January). x00EC; Losing weight, gaining fertilityi: a multidisciplinary lifestyle program for overweight women with polycystic ovary syndrome (PCOS), a study protocol. In *HUMAN REPRODUCTION* (Vol. 27). GREAT CLARENDON ST, OXFORD OX2 6DP, ENGLAND: OXFORD UNIV PRESS. - Daubenmier, J., Lin, J., Blackburn, E., Hecht, F. M., Kristeller, J., Maninger, N., ... & Epel, E. (2012). Changes in stress, eating, and metabolic factors are related to changes in telomerase activity in a randomized mindfulness intervention pilot study. *Psychoneuroendocrinology*, *37*(7), 917-928. - Braden, A., & O’Brien, W. (2021). Pilot study of a treatment using dialectical behavioral therapy skills for adults with overweight/obesity and emotional eating. *Journal of Contemporary Psychotherapy*, *51*, 21-29. - Olson, K. (2017). *Eating, exercise, and quality of life: The role of body image among adult women attempting weight loss* (Doctoral dissertation, The Ohio State University). - Fabricatore, A. N., Wadden, T. A., Womble, L. G., Sarwer, D. B., Berkowitz, R. I., Foster, G. D., & Brock, J. R. (2007). The role of patients' expectations and goals in the behavioral and pharmacological treatment of obesity. *International journal of obesity*, *31*(11), 1739-1745. - Mason, A. E., Laraia, B., Daubenmier, J., Hecht, F. M., Lustig, R. H., Puterman, E., ... & Epel, E. S. (2015). Putting the brakes on the “drive to eat”: Pilot effects of naltrexone and reward-based eating on food cravings among obese women. *Eating behaviors*, *19*, 53-56. - Bennion, K. A., Tate, D., Muñoz‐Christian, K., & Phelan, S. (2020). Impact of an Internet‐Based Lifestyle Intervention on Behavioral and Psychosocial Factors During Postpartum Weight Loss. *Obesity*, *28*(10), 1860-1867. - Davis, K. K. (2008). *Effect of mindfulness meditation and home-based resistance exercise on weight loss, weight loss behaviors, and psychosocial correlates in overweight adults* (Doctoral dissertation, University of Pittsburgh). - Jackson, J. B., Pietrabissa, G., Rossi, A., Manzoni, G. M., & Castelnuovo, G. (2018). Brief strategic therapy and cognitive behavioral therapy for women with binge eating disorder and comorbid obesity: A randomized clinical trial one-year follow-up. *Journal of consulting and clinical psychology*, *86*(8), 688. - Lillis, J., Niemeier, H. M., Ross, K. M., Thomas, J. G., Leahey, T., Unick, J., ... & Wing, R. R. (2015). Weight loss intervention for individuals with high internal disinhibition: design of the Acceptance Based Behavioral Intervention (ABBI) randomized controlled trial. *BMC psychology*, *3*, 1-10. - Hilbert, A., Neuhaus, P., Köhler, N., Petroff, D., Hay, P., & Hübner, C. (2021). Group cognitive remediation therapy prior to behavioral weight loss treatment for adults with severe obesity: A randomized clinical trial (CRT study). *Journal of Consulting and Clinical Psychology*, *89*(8), 695. - Optimizing an Online Behavioral Weight Loss Intervention and Novel Culturally Tailored Components for Sexual Minority Women, NCT05775497 - Sleep, Lifestyle, Energy, Eating, Exercise Program for the management of sleep apnea patients indicated for weight loss treatment: a randomised, controlled pilot study, ACTRN12613000191796 - Olson, K. L., Thaxton, T. T., Landers, J. D., & Emery, C. F. (2024). Evaluating the effect of targeting body shape concerns on long‐term weight change. *International Journal of Eating Disorders*, *57*(1), 201-205. - Yokoyama, H., Nozaki, T., Nishihara, T., Sawamoto, R., Komaki, G., & Sudo, N. (2022). Factors associated with the improvement of body image dissatisfaction of female patients with overweight and obesity during cognitive behavioral therapy. *Frontiers in Psychiatry*, *13*, 1025946. - Martínez, A. G., Avitia, G. C., & Mendez, Y. L. (2023). Multidisciplinary intervention and cognitive remediation therapy for adults with obesity: A study protocol for a randomized controlled clinical trial. *Contemporary Clinical Trials Communications*, *31*, 101041. - Bachand, A. R. (2008). *Changes in body image and body weight and shape goals associated with weight loss and maintenance in overweight/obese adults diagnosed with type 2 diabetes mellitus*. Louisiana State University and Agricultural & Mechanical College. - Dieterich-Hartwell, R., Sukumar, D., & Kaimal, G. (2023). A Creative Arts Therapy and Nutrition Education Approach for Postmenopausal Women. *Art Therapy*, 1-7. - González-Fernández, E., Xandri-Martínez, R., Gómez-Díaz, M., & Navas-López, J. (2024). Nutritional and Psychosocial Intervention to Improve the Self-Concept of Body Image and Increase the Self-Esteem of Overweight and Obese Individuals: A Quasi-Experimental Study. *Nutrients*, *16*(16), 2708. - Groshon, L. (2022). Longitudinal Associations of Binge Eating With Internalized Weight Stigma and Eating Self-Efficacy. *Obesity*, *30*, 150-150. - Muskan, F., Jain, S., & Bains, K. (2024). Efficacy of short-term cognitive group treatment to reduce obesity among overweight Indian women: a randomized control trial. *Current Science (00113891)*, *126*(3). - Effect of Different Types of Diet on Weight among Obese Individuals with Eating Disorders, IRCT20240629062275N1 | Body image questionnaire used or ineligible questionnaire |
| - Salvo, V., Kristeller, J., Montero Marin, J., Sanudo, A., Lourenço, B. H., Schveitzer, M. C., ... & Demarzo, M. (2018). Mindfulness as a complementary intervention in the treatment of overweight and obesity in primary health care: study protocol for a randomised controlled trial. *Trials*, *19*, 1-14. - Beutel, M. E., Dippel, A., Szczepanski, M., Thiede, R., & Wiltink, J. (2006). Mid-term effectiveness of behavioral and psychodynamic inpatient treatments of severe obesity based on a randomized study. *Psychotherapy and psychosomatics*, *75*(6), 337-345. - Fogarty, S., Stojanovska, L., Harris, D., Zaslawski, C., Mathai, M. L., & McAinch, A. J. (2015). A randomised cross-over pilot study investigating the use of acupuncture to promote weight loss and mental health in overweight and obese individuals participating in a weight loss program. *Eating and Weight Disorders-Studies on Anorexia, Bulimia and Obesity*, *20*, 379-387. - Seimon, R. V., Gibson, A. A., Harper, C., Keating, S. E., Johnson, N. A., Da Luz, F. Q., ... & Sainsbury, A. (2018, July). Rationale and protocol for a randomized controlled trial comparing fast versus slow weight loss in postmenopausal women with obesity—the TEMPO Diet Trial. In *Healthcare* (Vol. 6, No. 3, p. 85). MDPI. - Fogarty, S., Stojanovska, L., Harris, D., Zaslawski, C., Mathai, M. L., & McAinch, A. J. (2015). A randomised cross-over pilot study investigating the use of acupuncture to promote weight loss and mental health in overweight and obese individuals participating in a weight loss program. *Eating and Weight Disorders-Studies on Anorexia, Bulimia and Obesity*, *20*, 379-387. - Salvo, V., Sanudo, A., Kristeller, J., Schveitzer, M. C., Martins, P., Favarato, M. L., & Demarzo, M. (2022). Mindful eating for overweight and obese women in Brazil: An exploratory mixed-methods pilot study. *Nutrition and Health*, *28*(4), 591-601. - Foster, G. D., Wadden, T. A., Kendall, P. C., Stunkard, A. J., & Vogt, R. A. (1996). Psychological effects of weight loss and regain: a prospective evaluation. *Journal of Consulting and Clinical Psychology*, *64*(4), 752. - Isabelle, C., Christelle, C., Patrick, R., Tony, L., & Alain, G. (2011). Acceptance and efficacy of a guided internet self-help treatment program for obese patients with binge eating disorder. *Clinical practice and epidemiology in mental health: CP & EMH*, *7*, 8. - Anastasiadou, D., Slater, M., Spanlang, B., Porras, D. C., Comas, M., Ciudin, A., ... & Lusilla-Palacios, P. (2022). Clinical efficacy of a virtual reality tool for the treatment of obesity: study protocol of a randomised controlled trial. *BMJ open*, *12*(6), e060822. | Intervention not eligible |
| - Haugeland, B. J. (1995). *A treatment comparison of social rhythmicity in obese binge eaters* (Doctoral dissertation, University of Pittsburgh). - Mitchell, E. S., Yang, Q., Ho, A. S., Behr, H., May, C. N., DeLuca, L., & Michaelides, A. (2021). Self-reported nutritional factors are associated with weight loss at 18 months in a self-managed commercial program with food categorization system: Observational study. *Nutrients*, *13*(5), 1733. - Effect of Fasting and Calorie-Restricted Diets on Dopamine and Serotonin Levels Among Obese Women With BED and FA, ClinicalTrials.gov ID NCT04873648. Sponsor: University of Jordan, Information provided by zainab zueter, University of Jordan (Responsible Party) - Tufail, M. W., Khan, R., Shahadan, M. A., & Saleem, M. (2017). A psycho-physical intervention for waist circumference and waist hip ratio among pakistani women: a randomized control trial study. *Jurnal Psikologi Malaysia*, *31*(3). - Adding Guided Self-Help Group Therapy to the Alli Weight Loss Program in Treating Binge Eating Disorder, ClinicalTrials.gov ID NCT00601354, Sponsor: Stanford University, Information provided by Debra L. Safer, Stanford University (Responsible Party) - Boh, B., Lemmens, L. H., Jansen, A., Nederkoorn, C., Kerkhofs, V., Spanakis, G., ... & Roefs, A. (2016). An Ecological Momentary Intervention for weight loss and healthy eating via smartphone and Internet: study protocol for a randomised controlled trial. *Trials*, *17*, 1-12. - Lifestyle Intervention Plus Emotion Regulation Group Intervention Impact on Women's Cardiovascular Risk Reduction, ClinicalTrials.gov ID NCT03167489, Sponsor: Hadassah Medical Organization, Information provided by Donna R Zwas, Hadassah Medical Organization (Responsible Party) - Hahn, S. L., Pacanowski, C. R., Loth, K. A., Miller, J., Eisenberg, M. E., & Neumark-Sztainer, D. (2021). Self-weighing among young adults: who weighs themselves and for whom does weighing affect mood? A cross-sectional study of a population-based sample. *Journal of Eating Disorders*, *9*, 1-12. - Sherwood, N. E., Rock, C. L., Flatt, S. W., Karanja, N., Pakiz, B., & Thomson, C. (2010, October). Binge Status and Depression as Predictors of Weight Loss in a Multi-Site Randomized Trial of a Commercial Weight Loss Program. In *OBESITY* (Vol. 18, pp. S89-S89). 75 VARICK ST, 9TH FLR, NEW YORK, NY 10013-1917 USA: NATURE PUBLISHING GROUP. - A randomised controlled trial to compare time restricted eating with standard dietetic practices on glycaemic control in individuals with type 2 diabetes: a pilot study, https://anzctr.org.au/ACTRN12620000453987.aspx - Mindful Eating in the treatment of Obesity - ATENTO study - ATENTO Mindful Eating in the treatment of Obesity, RBR-22p3nn2, <http://ensaiosclinicos.gov.br/rg/RBR-22p3nn2> - Memory-Updating Technique to Reduce Food Craving and High Calorie Food Intake Among Individuals With Overweight/​Obesity, ClinicalTrials.gov ID NCT04077385, Sponsor: University of Pittsburgh, Information provided by Lisa J Germeroth, PhD, University of Pittsburgh (Responsible Party) - Adler, S. (2008). *Orlistat/alli as Compared to Orlistat/alli Plus Dialectical Behavior Therapy in Overweight Binge Eaters: A Randomized Control Trial* (Doctoral dissertation, Pacific Graduate School of Psychology). - Forman, E. M., Berry, M. P., Butryn, M. L., Hagerman, C. J., Huang, Z., Juarascio, A. S., ... & Zhang, F. (2023). Using artificial intelligence to optimize delivery of weight loss treatment: protocol for an efficacy and cost-effectiveness trial. *Contemporary clinical trials*, *124*, 107029. - ARIADNE: a Study of Weight Loss for Diabetes Treatment and Wellbeing, NCT05744232 - Boutelle, K. N., Afari, N., Obayashi, S., Eichen, D. M., Strong, D. R., & Peterson, C. B. (2023). Design of the CHARGE study: A randomized control trial evaluating a novel treatment for Veterans with binge eating disorder and overweight and obesity. *Contemporary Clinical Trials*, *130*, 107234. - Development of a Treatment Program for people with Binge eating and overweight or Obesity, RBR-10hvfd58 - Comparison of two weight loss programs involving 16 weeks on a severely energy-restricted diet in adults with overweight or obesity: the TANGO Diet Trial (Temporary phases of Accelerated weight loss for Noticeably Greater Outcomes), ACTRN12623000338662 - Effectiveness of different nutritional treatments for the health care of women with Obesity in Viçosa-MG, RBR-87wb8x5 - Effectiveness of Nutrition Communication, Empowerment and Leadership Training of Trainers (NutriCEL ToT) for Nutrition Students in Empowering Undergraduates to Practise Healthy Nutrition: a Cluster Randomised Controlled Trial, JPRN-UMIN000053636 - Targeting Weight and Shape Concern Among Women With High Body Weight, NCT05845866 | Results not published |
| - Mohorko, N., Černelič-Bizjak, M., Poklar-Vatovec, T., Grom, G., Kenig, S., Petelin, A., & Jenko-Pražnikar, Z. (2019). Weight loss, improved physical performance, cognitive function, eating behavior, and metabolic profile in a 12-week ketogenic diet in obese adults. *Nutrition research*, *62*, 64-77. - Jackson, J. B., Pietrabissa, G., Rossi, A., Manzoni, G. M., & Castelnuovo, G. (2018). Brief strategic therapy and cognitive behavioral therapy for women with binge eating disorder and comorbid obesity: A randomized clinical trial one-year follow-up. *Journal of consulting and clinical psychology*, *86*(8), 688. - Podina, I. R., Fodor, L. A., Cosmoiu, A., & Boian, R. (2017). An evidence-based gamified mHealth intervention for overweight young adults with maladaptive eating habits: study protocol for a randomized controlled trial. *Trials*, *18*, 1-14. - Messier, V., Rabasa-Lhoret, R., Doucet, E., Brochu, M., Lavoie, J. M., Karelis, A., ... & Strychar, I. (2010). Effects of the addition of a resistance training programme to a caloric restriction weight loss intervention on psychosocial factors in overweight and obese post-menopausal women: a Montreal Ottawa New Emerging Team study. *Journal of Sports Sciences*, *28*(1), 83-92. - Eldredge, K. L., Agras, W. S., Arnow, B., Telch, C. F., Bell, S., Castonguay, L., & Marnell, M. (1997). The effects of extending cognitive‐behavioral therapy for binge eating disorder among initial treatment nonresponders. *International Journal of Eating Disorders*, *21*(4), 347-352. - Grilo, C. (2015). Cardiovascular disease risk reduction in patients with binge eating disorder and obesity: Randomized controlled trial of stepped-care versus standard behavioral weight loss. *Atherosclerosis*, *241*(1), e19. - Alger, S. A., Malone, M., Cerulli, J., Fein, S., & Howard, L. (1999). Beneficial effects of pharmacotherapy on weight loss, depressive symptoms, and eating patterns in obese binge eaters and non‐binge eaters. *Obesity research*, *7*(5), 469-476. - Afari, N., Herbert, M. S., Godfrey, K. M., Cuneo, J. G., Salamat, J. S., Mostoufi, S., ... & Wetherell, J. L. (2019). Acceptance and commitment therapy as an adjunct to the MOVE! programme: a randomized controlled trial. *Obesity science & practice*, *5*(5), 397-407. - Robert, S. A., Rohana, A. G., Shah, S. A., Chinna, K., Mohamud, W. N. W., & Kamaruddin, N. A. (2015). Improvement in binge eating in non-diabetic obese individuals after 3 months of treatment with liraglutide–a pilot study. *Obesity research & clinical practice*, *9*(3), 301-304. - Rapoport, L., Clark, M., & Wardle, J. (2000). Evaluation of a modified cognitive–behavioural programme for weight management. *International journal of obesity*, *24*(12), 1726-1737. - Grilo, C. (2016). Randomized controlled trial testing behavioral weight loss versus multi-modal stepped-care treatment for binge eating disorder. *European Psychiatry*, *33*(S1), S163-S163. - Castelnuovo, G., Manzoni, G. M., Villa, V., Cesa, G. L., & Molinari, E. (2011). Brief strategic therapy vs cognitive behavioral therapy for the inpatient and telephone-based outpatient treatment of binge eating disorder: the STRATOB randomized controlled clinical trial. *Clinical practice and epidemiology in mental health: CP & EMH*, *7*, 29. - Golay, A., Laurent‐Jaccard, A., Habicht, F., Gachoud, J. P., Chabloz, M., Kammer, A., & Schutz, Y. (2005). Effect of orlistat in obese patients with binge eating disorder. *Obesity research*, *13*(10), 1701-1708. - Grilo, C. (2016). Randomized controlled trial testing behavioral weight loss versus multi-modal stepped-care treatment for binge eating disorder. *European Psychiatry*, *33*(S1), S163-S163. - Daley, A. J., Jolly, K., Bensoussane, H., Ives, N., Jebb, S. A., Tearne, S., ... & Parretti, H. M. (2020). Feasibility and acceptability of a brief routine weight management intervention for postnatal women embedded within the national child immunisation programme in primary care: randomised controlled cluster feasibility trial. *Trials*, *21*, 1-19. - Castelnuovo, G., Manzoni, G. M., Cuzziol, P., Cesa, G. L., Tuzzi, C., Villa, V., ... & Molinari, E. (2010). TECNOB: study design of a randomized controlled trial of a multidisciplinary telecare intervention for obese patients with type-2 diabetes. *BMC public health*, *10*, 1-8. - Carels, R. A., Wott, C. B., Young, K. M., Gumble, A., Koball, A., & Oehlhof, M. W. (2010). Implicit, explicit, and internalized weight bias and psychosocial maladjustment among treatment-seeking adults. *Eating behaviors*, *11*(3), 180-185. - MOON, K. T. (2010). Comparison of Psychological Treatments for Binge Eating Disorder. *American Family Physician*, *82*(5), 534-534. - Claudino, A. M., Oliveira, I. R. D., Appolinario, J. C., Cordás, T. A., Duchesne, M., Sichieri, R., & Bacaltchuk, J. (2007). Double-blind, randomized, placebo-controlled trial of topiramate plus cognitive-behavior therapy in binge-eating disorder. *Journal of Clinical Psychiatry*, *68*(9), 1324-1332. - Agras, W. S., Telch, C. F., Arnow, B., Eldredge, K., Wilfley, D. E., Raeburn, S. D., ... & Marnell, M. (1994). Weight loss, cognitive-behavioral, and desipramine treatments in binge eating disorder. An additive design. *Behavior Therapy*, *25*(2), 225-238. - Bolognese, M. A., Franco, C. B., Ferrari, A., Bennemann, R. M., Lopes, S. M. A., Bertolini, S. M. M. G., ... & Branco, B. H. M. (2020). Group nutrition counseling or individualized prescription for women with obesity? A clinical trial. *Frontiers in public health*, *8*, 127. - Delparte, C. A., Power, H. A., Gelinas, B. L., Oliver, A. M., Hart, R. D., & Wright, K. D. (2019). Examination of the effectiveness of a brief, adapted dialectical behavior therapy-skills training group for bariatric surgical candidates. *Obesity surgery*, *29*, 252-261. - Duchesne, M., Appolinario, J. C., Rangé, B. P., Fandiño, J., Moya, T., & Freitas, S. R. (2007). The use of a manual-driven group cognitive behavior therapy in a Brazilian sample of obese individuals with binge-eating disorder. *Brazilian Journal of Psychiatry*, *29*, 23-25. - McElroy, S. L. (2003). Topiramate in the treatment of binge eating disorder associated with obesity: A randomized, placebo-controlled trial (vol 160, pg 255, 2003). *AMERICAN JOURNAL OF PSYCHIATRY*, *160*(3), 612-612. - Keränen, A. M., Savolainen, M. J., Reponen, A. H., Kujari, M. L., Lindeman, S. M., Bloigu, R. S., & Laitinen, J. H. (2009). The effect of eating behavior on weight loss and maintenance during a lifestyle intervention. *Preventive medicine*, *49*(1), 32-38. - Yanovski, S. Z., & Sebring, N. G. (1994). Recorded food intake of obese women with binge eating disorder before and after weight loss. *International Journal of eating disorders*, *15*(2), 135-150. - Ruffault, A., Carette, C., i Puerto, K. L., Juge, N., Beauchet, A., Benoliel, J. J., ... & Flahault, C. (2016). Randomized controlled trial of a 12-month computerized mindfulness-based intervention for obese patients with binge eating disorder: The MindOb study protocol. *Contemporary clinical trials*, *49*, 126-133. - De Zwaan, M., Mitchell, J. E., Crosby, R. D., Mussell, M. P., Raymond, N. C., Specker, S. M., & Seim, H. C. (2005). Short-term cognitive behavioral treatment does not improve outcome of a comprehensive very-low-calorie diet program in obese women with binge eating disorder. *Behavior therapy*, *36*(1), 89-99. - Painot, D., Jotterand, S., Kammer, A., Fossati, M., & Golay, A. (2001). Simultaneous nutritional cognitive–behavioural therapy in obese patients. *Patient education and counseling*, *42*(1), 47-52. - Bacon, L., Keim, N. L., Van Loan, M. D., Derricote, M., Gale, B., Kazaks, A., & Stern, J. (2002). Evaluating a ‘non-diet’wellness intervention for improvement of metabolic fitness, psychological well-being and eating and activity behaviors. *International journal of obesity*, *26*(6), 854-865. - Piñera, M. J., Arrieta, F. J., Alcaraz-Cebrián, F., Botella-Carretero, J. I., Calañas, A., Balsa, J. A., ... & Vázquez, C. (2012). Influence of weight loss in the clinical evolution, metabolic and psychological of the patients with overweight or obesity. *Nutricion Hospitalaria*, *27*(5), 1480-1488. - Papini, N. M., Foster, R. N., Lopez, N. V., Ptomey, L. T., Herrmann, S. D., & Donnelly, J. E. (2022). Examination of three-factor eating questionnaire subscale scores on weight loss and weight loss maintenance in a clinical intervention. *BMC psychology*, *10*(1), 101. - Agras, W. S., Telch, C. F., Arnow, B., Eldredge, K., Detzer, M. J., Henderson, J., & Marnell, M. (1995). Does interpersonal therapy help patients with binge eating disorder who fail to respond to cognitive-behavioral therapy?. *Journal of consulting and clinical psychology*, *63*(3), 356. - Guerdjikova, A. I., Walsh, B., Shan, K., Halseth, A. E., Dunayevich, E., & McElroy, S. L. (2017). Concurrent improvement in both binge eating and depressive symptoms with naltrexone/bupropion therapy in overweight or obese subjects with major depressive disorder in an open-label, uncontrolled study. *Advances in Therapy*, *34*, 2307-2315. - Chao, A. M., Wadden, T. A., Pearl, R. L., Alamuddin, N., Leonard, S. M., Bakizada, Z. M., ... & Tronieri, J. S. (2018). A randomized controlled trial of lorcaserin and lifestyle counselling for weight loss maintenance: changes in emotion‐and stress‐related eating, food cravings and appetite. *Clinical obesity*, *8*(6), 383-390. - Pacanowski, C. R., & Levitsky, D. A. (2013). Effectiveness of losing weight slowly for producing and maintaining weight loss in overweight and obese adults: 24 month results. - Fogelholm, M., Kukkonen-Harjula, K., & Oja, P. (1999). Eating control and physical activity as determinants of short-term weight maintenance after a very-low-calorie diet among obese women. *International journal of obesity*, *23*(2), 203-210. - Mama, S. K., Schembre, S. M., O'Connor, D. P., Kaplan, C. D., Bode, S., & Lee, R. E. (2015). Effectiveness of lifestyle interventions to reduce binge eating symptoms in African American and Hispanic women. *Appetite*, *95*, 269-274. - LaRose, J. G., Fava, J. L., Steeves, E. A., Hecht, J., Wing, R. R., & Raynor, H. A. (2014). Daily self-weighing within a lifestyle intervention: impact on disordered eating symptoms. *Health Psychology*, *33*(3), 297. - Wadden, T. A., Foster, G. D., Sarwer, D. B., Anderson, D. A., Gladis, M., Sanderson, R. S., ... & Phelan, S. (2004). Dieting and the development of eating disorders in obese women: results of a randomized controlled trial. *The American journal of clinical nutrition*, *80*(3), 560-568. - Lundgren, J. D. (2004). *A mindfulness-based behavioral treatment for weight loss*. State University of New York at Albany. - Friederich, H. C., Schild, S., Wild, B., De Zwaan, M., Quenter, A., Herzog, W., & Zipfel, S. (2007). Treatment outcome in people with subthreshold compared with full‐syndrome binge eating disorder. *Obesity*, *15*(2), 283-287. - Compare, A., Calugi, S., Marchesini, G., Shonin, E., Grossi, E., Molinari, E., & Dalle Grave, R. (2013). Emotionally focused group therapy and dietary counseling in binge eating disorder. Effect on eating disorder psychopathology and quality of life. *Appetite*, *71*, 361-368. - Steinberg, D. M., Tate, D. F., Bennett, G. G., Ennett, S., Samuel-Hodge, C., & Ward, D. S. (2014). Daily self-weighing and adverse psychological outcomes: a randomized controlled trial. *American Journal of Preventive Medicine*, *46*(1), 24-29. - Tseng, M. C., Lee, M. B., Chen, S. Y., Lee, Y. J., Lin, K. H., Chen, P. R., & Lai, J. S. (2004). Response of Taiwanese obese binge eaters to a hospital-based weight reduction program. *Journal of Psychosomatic Research*, *57*(3), 279-285. - Svendsen, M., Rissanen, A., Richelsen, B., Rössner, S., Hansson, F., & Tonstad, S. (2008). Effect of orlistat on eating behavior among participants in a 3‐year weight maintenance trial. *Obesity*, *16*(2), 327-333. - Carels, R. A., Burmeister, J. M., Koball, A. M., Oehlhof, M. W., Hinman, N., LeRoy, M., ... & Gumble, A. (2014). A randomized trial comparing two approaches to weight loss: differences in weight loss maintenance. *Journal of health psychology*, *19*(2), 296-311. - Tanco, S. A. (1995). *Well-being and morbid obesity in women* (Doctoral dissertation, University of British Columbia). - Cheng, H. L., Griffin, H., Claes, B. E., Petocz, P., Steinbeck, K., Rooney, K., & O’Connor, H. (2014). Influence of dietary macronutrient composition on eating behaviour and self-perception in young women undergoing weight management. *Eating and Weight Disorders-Studies on Anorexia, Bulimia and Obesity*, *19*, 241-247. - Ruffault, A., Carette, C., i Puerto, K. L., Juge, N., Beauchet, A., Benoliel, J. J., ... & Flahault, C. (2016). Randomized controlled trial of a 12-month computerized mindfulness-based intervention for obese patients with binge eating disorder: The MindOb study protocol. *Contemporary clinical trials*, *49*, 126-133. - Castelnuovo, G., Manzoni, G. M., Villa, V., Cesa, G. L., Pietrabissa, G., & Molinari, E. (2011). The STRATOB study: design of a randomized controlled clinical trial of Cognitive Behavioral Therapy and Brief Strategic Therapy with telecare in patients with obesity and binge-eating disorder referred to residential nutritional rehabilitation. *Trials*, *12*, 1-7. - Afari, N., Cuneo, J. G., Herbert, M., Miller, I., Webb-Murphy, J., Delaney, E., ... & Wisbach, G. (2019). Design for a cohort-randomized trial of an acceptance and commitment therapy-enhanced weight management and fitness program for Navy personnel. *Contemporary clinical trials communications*, *15*, 100408. - Goodrick, G. K., Poston II, W. S. C., Kimball, K. T., Reeves, R. S., & Foreyt, J. P. (1998). Nondieting versus dieting treatment for overweight binge-eating women. *Journal of consulting and clinical psychology*, *66*(2), 363. - Hoddy, K. K., Kroeger, C. M., Trepanowski, J. F., Barnosky, A. R., Bhutani, S., & Varady, K. A. (2015). Safety of alternate day fasting and effect on disordered eating behaviors. *Nutrition journal*, *14*, 1-3. - Blevins, N. C. (2008). *Mindfulness meditation as an intervention for body image and weight management in college women: A pilot study* (Doctoral dissertation, University of Florida). - Beaulieu, K., Casanova, N., Oustric, P., Turicchi, J., Gibbons, C., Hopkins, M., ... & Finlayson, G. (2020). Matched weight loss through intermittent or continuous energy restriction does not lead to compensatory increases in appetite and eating behavior in a randomized controlled trial in women with overweight and obesity. *The Journal of nutrition*, *150*(3), 623-633. - Devlin, M. J., Goldfein, J. A., Petkova, E., Jiang, H., Raizman, P. S., Wolk, S., ... & Walsh, B. T. (2005). Cognitive behavioral therapy and fluoxetine as adjuncts to group behavioral therapy for binge eating disorder. *Obesity research*, *13*(6), 1077-1088. - Cash, T. F. (1993). Body-image attitudes among obese enrollees in a commercial weight-loss program. *Perceptual and Motor skills*, *77*(3_suppl), 1099-1103. - Grilo, C. M., Kerrigan, S. G., Lydecker, J. A., & White, M. A. (2021). Physical activity changes during behavioral weight loss treatment by Latinx patients with obesity with and without binge eating disorder. *Obesity*, *29*(12), 2026-2034. - Robert, S. A., Ghani, R. A., Zainuddin, S., Shah, S. A., Mohamud, W. N. W., & Kamaruddin, N. A. (2013). The influence of a GLP-1 analogue, liraglutide on binge eating behaviour among obese healthy participants. *Obesity Research & Clinical Practice*, (7), e39. - Podina, I. R., Fodor, L. A., Cosmoiu, A., & Boian, R. (2017). An evidence-based gamified mHealth intervention for overweight young adults with maladaptive eating habits: study protocol for a randomized controlled trial. *Trials*, *18*, 1-14. - Minniti, A., Bissoli, L., Di Francesco, V., Fantin, F., Mandragona, R., Olivieri, M., ... & Zamboni, M. (2007). Individual versus group therapy for obesity: comparison of dropout rate and treatment outcome. *Eating and Weight Disorders-Studies on Anorexia, Bulimia and Obesity*, *12*, 161-167. - Williamson, D. A., Martin, C. K., Anton, S. D., York-Crowe, E., Han, H., Redman, L., & Ravussin, E. (2008). Is caloric restriction associated with development of eating-disorder symptoms? Results from the CALERIE trial. *Health Psychology*, *27*(1S), S32. - Molinari, E., Baruffi, M., Croci, M., Marchi, S., & Petroni, M. L. (2005). Binge eating disorder in obesity: comparison of different therapeutic strategies. *Eating and Weight Disorders-Studies on Anorexia, Bulimia and Obesity*, *10*, 154-161. - Teixeira, P. J., Going, S. B., Houtkooper, L. B., Cussler, E. C., Metcalfe, L. L., Blew, R. M., ... & Lohman, T. G. (2006). Exercise motivation, eating, and body image variables as predictors of weight control. *Medicine & science in sports & Exercise*, *38*(1), 179-188. - Donini, L. M., Cuzzolaro, M., Gnessi, L., Lubrano, C., Migliaccio, S., Aversa, A., ... & Lenzi, A. (2014). Obesity treatment: results after 4 years of a Nutritional and Psycho-Physical Rehabilitation Program in an outpatient setting. *Eating and Weight Disorders-Studies on Anorexia, Bulimia and Obesity*, *19*, 249-260. - A Study of the Effectiveness and Safety of Topiramate in the Treatment of Moderate to Severe Binge-eating Disorder Associated With Obesity, ClinicalTrials.gov ID NCT00210808, Sponsor Johnson & Johnson Pharmaceutical Research & Development, L.L.C., Information provided by Johnson & Johnson Pharmaceutical Research & Development, L.L.C. - Annesi, J. J., & Johnson, P. H. (2015). Theory-based psychosocial factors that discriminate between weight-loss success and failure over 6 months in women with morbid obesity receiving behavioral treatments. *Eating and Weight Disorders-Studies on Anorexia, Bulimia and Obesity*, *20*, 223-232. - Osei-Assibey, G., Kyrou, I., Kumar, S., Saravanan, P., & Matyka, K. A. (2010). Self-reported psychosocial health in obese patients before and after weight loss. *Journal of Obesity*, *2010*. - Telch, C. F., & Agras, W. S. (1993). The effects of a very low calorie diet on binge eating. *Behavior Therapy*, *24*(2), 177-193. - Afari, N., Herbert, M. S., Godfrey, K. M., Cuneo, J. G., Salamat, J. S., Mostoufi, S., ... & Wetherell, J. L. (2019). Acceptance and commitment therapy as an adjunct to the MOVE! programme: a randomized controlled trial. *Obesity science & practice*, *5*(5), 397-407. - Carraça, E. V., Silva, M. N., Markland, D., Vieira, P. N., Minderico, C. S., Sardinha, L. B., & Teixeira, P. J. (2011). Body image change and improved eating self-regulation in a weight management intervention in women. *International Journal of Behavioral Nutrition and Physical Activity*, *8*, 1-11. - Pataky, Z., Gasteyger, C., Ziegler, O., Rissanen, A., Hanotin, C., & Golay, A. (2013). Efficacy of rimonabant in obese patients with binge eating disorder. *Experimental and Clinical Endocrinology & Diabetes*, *121*(01), 20-26. - Agras, W. S., Telch, C. F., Arnow, B., Eldredge, K., & Marnell, M. (1997). One-year follow-up of cognitive-behavioral therapy for obese individuals with binge eating disorder. *Journal of consulting and Clinical Psychology*, *65*(2), 343. - Annunziato, R. A., Timko, C. A., Crerand, C. E., Didie, E. R., Bellace, D. L., Phelan, S., ... & Lowe, M. R. (2009). A randomized trial examining differential meal replacement adherence in a weight loss maintenance program after one-year follow-up. *Eating behaviors*, *10*(3), 176-183. - Grave, R. D., Calugi, S., Ruocco, A., & Marchesini, G. (2011). Night eating syndrome and weight loss outcome in obese patients. *International Journal of Eating Disorders*, *44*(2), 150-156. - Masheb, R. M., Grilo, C. M., & Rolls, B. J. (2011, November). An RCT For Obesity and Binge Eating Disorder: Low-Energy-Density Nutrition Counseling and Cognitive Behavioral Therapy. In *OBESITY* (Vol. 19, pp. S113-S114). 75 VARICK ST, 9TH FLR, NEW YORK, NY 10013-1917 USA: NATURE PUBLISHING GROUP. - dos Santos Moraes, A., Cipullo, M. A. T., Poli, V. F. S., Rebelo, R. A., Ribeiro, E. B., Oyama, L. M., ... & Caranti, D. A. (2019). Neuroendocrine control, inflammation, and psychological aspects after interdisciplinary therapy in obese women. *Hormone and Metabolic Research*, *51*(06), 375-380. - Rieger, E., Treasure, J., Murray, K., & Caterson, I. (2017). The use of support people to improve the weight-related and psychological outcomes of adults with obesity: A randomised controlled trial. *Behaviour Research and Therapy*, *94*, 48-59. - Teixeira, P. J., Silva, M. N., Coutinho, S. R., Palmeira, A. L., Mata, J., Vieira, P. N., ... & Sardinha, L. B. (2010). Mediators of weight loss and weight loss maintenance in middle‐aged women. *Obesity*, *18*(4), 725-735. - Grilo, C. (2016). [pp. 13.07] Hypertension and cardiovascular disease risk reduction in obese patients with binge eating disorder: controlled trial of stepped-care and behavioral weight loss. *Journal of Hypertension*, *34*, e192-e193. - Molinari, E., Baruffi, M., Croci, M., Marchi, S., & Petroni, M. L. (2005). Binge eating disorder in obesity: comparison of different therapeutic strategies. *Eating and Weight Disorders-Studies on Anorexia, Bulimia and Obesity*, *10*, 154-161. - Pekkarinen, T., Takala, I., & Mustajoki, P. (1996). Two year maintenance of weight loss after a VLCD and behavioural therapy for obesity: correlation to the scores of questionnaires measuring eating behaviour. *International journal of obesity and related metabolic disorders: journal of the International Association for the Study of Obesity*, *20*(4), 332-337. - Carvalho-Ferreira, J. P. D., Cipullo, M. A. T., Caranti, D. A., Masquio, D. C. L., Andrade-Silva, S. G., Pisani, L. P., & Dâmaso, A. R. (2012). Interdisciplinary lifestyle therapy improves binge eating symptoms and body image dissatisfaction in Brazilian obese adults. *Trends in psychiatry and psychotherapy*, *34*, 223-233. - Shapiro, J. R., Reba‐Harrelson, L., Dymek‐Valentine, M., Woolson, S. L., Hamer, R. M., & Bulik, C. M. (2007). Feasibility and acceptability of CD‐ROM‐based cognitive‐behavioural treatment for binge‐eating disorder. *European Eating Disorders Review: The Professional Journal of the Eating Disorders Association*, *15*(3), 175-184. - Teong, X. T., Hutchison, A. T., Liu, B., Wittert, G. A., Lange, K., Banks, S., & Heilbronn, L. K. (2021). Eight weeks of intermittent fasting versus calorie restriction does not alter eating behaviors, mood, sleep quality, quality of life and cognitive performance in women with overweight. *Nutrition Research*, *92*, 32-39. - Rieger, E., Treasure, J., Murray, K., & Caterson, I. (2017). The use of support people to improve the weight-related and psychological outcomes of adults with obesity: A randomised controlled trial. *Behaviour Research and Therapy*, *94*, 48-59. - Devlin, M. J., Goldfein, J. A., Carino, J. S., & Wolk, S. L. (2000). Open treatment of overweight binge eaters with phentermine and fluoxetine as an adjunct to cognitive‐behavioral therapy. *International Journal of Eating Disorders*, *28*(3), 325-332. - Compare, A., Calugi, S., Marchesini, G., Molinari, E., & Dalle Grave, R. (2013). Emotion-focused therapy and dietary counseling for obese patients with binge eating disorder: A propensity score-adjusted study. *Psychotherapy and Psychosomatics*, *82*(3), 193-194. - Leite, P. B., Dâmaso, A. R., Poli, V. S., Sanches, R. B., Silva, S. G. A., Fidalgo, J. P. N., ... & Caranti, D. A. (2017). Long-term interdisciplinary therapy decreases symptoms of binge eating disorder and prevalence of metabolic syndrome in adults with obesity. *Nutrition research*, *40*, 57-64. - Bacon, L., Stern, J. S., Van Loan, M. D., & Keim, N. L. (2005). Size acceptance and intuitive eating improve health for obese, female chronic dieters. *Journal of the American Dietetic Association*, *105*(6), 929-936. - Bégin, C., Carbonneau, E., Gagnon-Girouard, M. P., Mongeau, L., Paquette, M. C., Turcotte, M., & Provencher, V. (2019). Eating-related and psychological outcomes of health at every size intervention in health and social services centers across the Province of Quebec. *American Journal of Health Promotion*, *33*(2), 248-258. - Pietrabissa, G., Manzoni, G. M., Ceccarini, M., & Castelnuovo, G. (2014). A brief strategic therapy protocol for binge eating disorder. *Procedia-Social and Behavioral Sciences*, *113*, 8-15. - Zwickert, K., Rieger, E., Swinbourne, J., Manns, C., McAulay, C., Gibson, A. A., ... & Caterson, I. D. (2016). High or low intensity text-messaging combined with group treatment equally promote weight loss maintenance in obese adults. *Obesity research & clinical practice*, *10*(6), 680-691. - Rudolph, A., Hellbardt, M., Baldofski, S., de Zwaan, M., & Hilbert, A. (2016). Evaluation of the one-year multimodal weight loss program DOC WEIGHT® 1.0 for obesity class II and III. *Psychotherapie, Psychosomatik, Medizinische Psychologie*, *66*(8), 316-323. - Raynaud, A. S. (1999). *Evaluation of body image, self-esteem, and binge eating after treatment with behavioral self-management or food dependency regimes in obese females who binge eat*. University of Houston. - Mason, C., de Dieu Tapsoba, J., Duggan, C., Wang, C. Y., Alfano, C. M., & McTiernan, A. (2019). Eating behaviors and weight loss outcomes in a 12-month randomized trial of diet and/or exercise intervention in postmenopausal women. *International Journal of Behavioral Nutrition and Physical Activity*, *16*, 1-11. - Schaumberg, K., Anderson, D. A., Kirschenbaum, D. S., & Earleywine, M. (2015). Participation as a leader in immersion weight loss treatment may benefit, not harm, young adult staff members. *Clinical Obesity*, *5*(4), 226-235. - Mason, A. E., Lustig, R. H., Brown, R. R., Acree, M., Bacchetti, P., Moran, P. J., ... & Epel, E. S. (2015). Acute responses to opioidergic blockade as a biomarker of hedonic eating among obese women enrolled in a mindfulness-based weight loss intervention trial. *Appetite*, *91*, 311-320. - Dalle Grave, R., Cuzzolaro, M., Calugi, S., Tomasi, F., Temperilli, F., Marchesini, G., & QUOVADIS Study Group. (2007). The effect of obesity management on body image in patients seeking treatment at medical centers. *Obesity*, *15*(9), 2320-2327. - Sbrocco, T., Carter, M. M., Lewis, E. L., Vaughn, N. A., Kalupa, K. L., King, S., ... & Cintrón, J. A. (2005). Church-based obesity treatment for African-American women improves adherence. *Ethnicity & disease*, *15*(2), 246. - Slawson, D. (2003). Topiramate for obesity-related binge eating disorder. *American Family Physician*, *67*(12), 2580-2580. - Munsch, S., Biedert, E., & Keller, U. (2003). Evaluation of a lifestyle change programme for the treatment of obesity in general practice. *Swiss medical weekly*, *133*, 148-154. - Rasson, S., 2022, June. Reducing Eating disorders with a multidisciplinary intervention containing cognitive therapy, nutrition and physical exercise in overweighed adults between years 2016 and 2018. In *ANNALES MEDICO-PSYCHOLOGIQUES* (Vol. 180, No. 6, pp. 495-502). 21 STREET CAMILLE DESMOULINS, ISSY, 92789 MOULINEAUX CEDEX 9, FRANCE: MASSON EDITEUR. - Groshon, L. C., & Pearl, R. L. (2023). Longitudinal associations of binge eating with internalized weight stigma and eating self-efficacy. *Eating Behaviors*, *50*, 101785. - Goldstein, S. P., Olson, K. L., & Thomas, J. G. (2023). Association of weight and shape concern with weight change and weight-related behaviors in behavioral weight loss treatment. *Journal of Behavioral Medicine*, *46*(6), 1049-1056. - Choi, B. S. Y., Brunelle, L., Pilon, G., Cautela, B. G., Tompkins, T. A., Drapeau, V., ... & Tremblay, A. (2023). Lacticaseibacillus rhamnosus HA-114 improves eating behaviors and mood-related factors in adults with overweight during weight loss: A randomized controlled trial. *Nutritional Neuroscience*, *26*(7), 667-679. - Morillo‐Sarto, H., López‐del‐Hoyo, Y., Pérez‐Aranda, A., Modrego‐Alarcón, M., Barceló‐Soler, A., Borao, L., ... & Montero‐Marin, J. (2023). ‘Mindful eating’for reducing emotional eating in patients with overweight or obesity in primary care settings: A randomized controlled trial. *European Eating Disorders Review*, *31*(2), 303-319. - Kataoka, J., Stener‐Victorin, E., Schmidt, J., & Larsson, I. (2024). A prospective 12‐month structured weight loss intervention in women with severe obesity and polycystic ovary syndrome: Impact of weight loss on eating behaviors. *Acta Obstetricia et Gynecologica Scandinavica*. - Salvo, V., Curado, D. F., Sanudo, A., Kristeller, J., Schveitzer, M. C., Favarato, M. L., ... & Demarzo, M. (2022). Comparative effectiveness of mindfulness and mindful eating programmes among low-income overweight women in primary health care: A randomised controlled pragmatic study with psychological, biochemical, and anthropometric outcomes. *Appetite*, *177*, 106131. - Pepe, R. B., Coelho, G. S. D. M. A., da Silva Miguel, F., Gualassi, A. C., Sarvas, M. M., Cercato, C., ... & de Melo, M. E. (2023). Mindful eating for weight loss in women with obesity: a randomised controlled trial. *British Journal of Nutrition*, *130*(5), 911-920. - Fagundes, G. B. P., Tibães, J. R. B., Silva, M. L., Braga, M. M., Silveira, A. L. M., Teixeira, A. L., & Ferreira, A. V. M. (2023). Metabolic and behavioral effects of time-restricted eating in women with overweight or obesity: Preliminary findings from a randomized study. *Nutrition*, *107*, 111909. - Björkman, S., Höskuldsdóttir, G., Mossberg, K., Laurenius, A., Engström, M., Fändriks, L., ... & Larsson, I. (2024). Impact of eating behavior on 24‐month weight change after treatment of severe obesity—A clinical prospective cohort study. *Obesity*. - Salvo, V., Sanudo, A., Kristeller, J., Schveitzer, M. C., Martins, P., Favarato, M. L., & Demarzo, M. (2022). Mindful eating for overweight and obese women in Brazil: An exploratory mixed-methods pilot study. *Nutrition and Health*, *28*(4), 591-601. - Morillo‐Sarto, H., López‐del‐Hoyo, Y., Pérez‐Aranda, A., Modrego‐Alarcón, M., Barceló‐Soler, A., Borao, L., ... & Montero‐Marin, J. (2023). ‘Mindful eating’for reducing emotional eating in patients with overweight or obesity in primary care settings: A randomized controlled trial. *European Eating Disorders Review*, *31*(2), 303-319. - Félix-Alcántara, M. P., Villacañas-Blázquez, M., Banzo-Arguis, C., Domínguez-García, C., Gutiérrez-Arana, I., Hermosillo-Torres, R., & Quintero, J. (2023). Desarrollo del programa PsicoObe, una intervención psicoterapéutica grupal para personas con obesidad, y estudio piloto sobre sus efectos. *Revista de Psicopatología y Psicología Clínica*, *28*(2), 83-98. - Annesi, J. J., & Powell, S. M. (2023). Effects of Women's Body Satisfaction, Emotional Eating, and Race on Short-, Mid-, and Long-term Weight Loss. *Journal of Nutrition Education and Behavior*, *55*(10), 743-747. - Annesi, J. J., & Powell, S. M. (2023). Effects of Women's Body Satisfaction, Emotional Eating, and Race on Short-, Mid-, and Long-term Weight Loss. *Journal of Nutrition Education and Behavior*, *55*(10), 743-747. - Irani, H., Abiri, B., Khodami, B., Yari, Z., Lafzi Ghazi, M., Hosseinzadeh, N., & Saidpour, A. (2024). Effect of time restricted feeding on anthropometric measures, eating behavior, stress, serum levels of BDNF and LBP in overweight/obese women with food addiction: A randomized clinical trial. *Nutritional Neuroscience*, *27*(6), 577-589. - Duarte, C., Gilbert, P., Stalker, C., Catarino, F., Basran, J., Scott, S., ... & Stubbs, R. J. (2021). Effect of adding a compassion-focused intervention on emotion, eating and weight outcomes in a commercial weight management programme. *Journal of health psychology*, *26*(10), 1700-1715. - Ghafouri-Taleghani, F., Tafreshi, A. S., Doost, A. H., Tabesh, M., Abolhasani, M., Amini, A., & Saidpour, A. (2024). Effects of probiotic supplementation added to a weight loss program on anthropometric measures, body composition, eating behavior, and related hormone levels in patients with food addiction and weight regain after bariatric surgery: a randomized clinical trial. *Obesity Surgery*, *34*(9), 3181-3194. - Minari, T. P., Araújo-Filho, G. M. D., Tácito, L. H. B., Yugar, L. B. T., Rubio, T. D. A., Pires, A. C., ... & Moreno, H. (2024). Effects of Mindful Eating in Patients with Obesity and Binge Eating Disorder. *Nutrients*, *16*(6), 884. - Berman, M. I., Park, J., Kragenbrink, M. E., & Hegel, M. T. (2022). Accept yourself! A pilot randomized controlled trial of a self-acceptance-based treatment for large-bodied women with depression. *Behavior Therapy*, *53*(5), 913-926. | Secondary outcome questionnaire used (not analysed here) |
| - Marazziti, D., Rossi, L., Baroni, S., Consoli, G., Hollander, E., & Catena-Dell'Osso, M. (2011). Novel treatment options of binge eating disorder. *Current medicinal chemistry*, *18*(33), 5159-5164. - Palavras, M. A., Hay, P., dos Santos Filho, C. A., & Claudino, A. (2017). The efficacy of psychological therapies in reducing weight and binge eating in people with bulimia nervosa and binge eating disorder who are overweight or obese—a critical synthesis and meta-analyses. *Nutrients*, *9*(3), 299. - Jones, R. A., Lawlor, E. R., Birch, J. M., Patel, M. I., Werneck, A. O., Hoare, E., ... & Ahern, A. L. (2021). The impact of adult behavioural weight management interventions on mental health: A systematic review and meta‐analysis. *Obesity Reviews*, *22*(4), e13150. - Amodeo, G., Cuomo, A., Bolognesi, S., Goracci, A., Trusso, M. A., Piccinni, A., ... & Fagiolini, A. (2019). Pharmacotherapeutic strategies for treating binge eating disorder. Evidence from clinical trials and implications for clinical practice. *Expert opinion on pharmacotherapy*, *20*(6), 679-690. - Lasikiewicz, N., Myrissa, K., Hoyland, A., & Lawton, C. L. (2014). Psychological benefits of weight loss following behavioural and/or dietary weight loss interventions. A systematic research review. *Appetite*, *72*, 123-137. - Da Luz, F. Q., Hay, P., Gibson, A. A., Touyz, S. W., Swinbourne, J. M., Roekenes, J. A., & Sainsbury, A. (2015). Does severe dietary energy restriction increase binge eating in overweight or obese individuals? A systematic review. *Obesity Reviews*, *16*(8), 652-665. - Martín-Mariscal, V. (2017). A systematic review and narrative synthesis of interventions for uncomplicated obesity: weight loss, well-being and impact on eating disorder. *Enfermeria Clinica (English Edition)*, *28*(3), 212-213. - Jacob, A., Moullec, G., Lavoie, K. L., Laurin, C., Cowan, T., Tisshaw, C., ... & Bacon, S. L. (2018). Impact of cognitive-behavioral interventions on weight loss and psychological outcomes: A meta-analysis. *Health Psychology*, *37*(5), 417. - Godsey, J. (2013). The role of mindfulness based interventions in the treatment of obesity and eating disorders: an integrative review. *Complementary therapies in medicine*, *21*(4), 430-439. - Peckmezian, T., & Hay, P. (2017). A systematic review and narrative synthesis of interventions for uncomplicated obesity: weight loss, well-being and impact on eating disorders. *Journal of eating disorders*, *5*, 1-15. - Ruffault, A., Czernichow, S., Hagger, M. S., Ferrand, M., Erichot, N., Carette, C., ... & Flahault, C. (2017). The effects of mindfulness training on weight-loss and health-related behaviours in adults with overweight and obesity: A systematic review and meta-analysis. *Obesity research & clinical practice*, *11*(5), 90-111. - Kantilafti, M., Chrysostomou, S., Yannakoulia, M., & Giannakou, K. (2022). The association between binge eating disorder and weight management in overweight and obese adults: A systematic literature review. *Nutrition and Health*, *28*(2), 189-197. - Tata, A. L., & Kockler, D. R. (2006). Topiramate for binge-eating disorder associated with obesity. *Annals of Pharmacotherapy*, *40*(11), 1993-1997. - Silverii, G. A., Cresci, B., Benvenuti, F., Santagiuliana, F., Rotella, F., & Mannucci, E. (2023). Effectiveness of intermittent fasting for weight loss in individuals with obesity: A meta-analysis of randomized controlled trials. *Nutrition, Metabolism and Cardiovascular Diseases*, *33*(8), 1481-1489. - Clarke, E. D., Stanford, J., Gomez‐Martin, M., & Collins, C. E. (2024). Revisiting the impact of Health at Every Size® interventions on health and cardiometabolic related outcomes: An updated systematic review with meta‐analysis. *Nutrition & Dietetics*. - Chew, H. S. J., Chng, S., Rajasegaran, N. N., Choy, K. H., & Chong, Y. Y. (2023). Effectiveness of acceptance and commitment therapy on weight, eating behaviours and psychological outcomes: a systematic review and meta-analysis. *Eating and Weight Disorders-Studies on Anorexia, Bulimia and Obesity*, *28*(1), 6. - Wajid, I., Vega, A., Thornhill, K., Jenkins, J., Merriman, C., Chandler, D., ... & Kaye, A. D. (2023). Topiramate (Topamax): Evolving role in weight reduction management: a narrative review. *Life*, *13*(9), 1845. - Forman, E. M., Evans, B. C., Berry, M. P., Lampe, E. W., Chwyl, C., & Zhang, F. (2023). Behavioral weight loss outcomes in individuals with binge‐eating disorder: A meta‐analysis. *Obesity*, *31*(8), 1981-1995. - Lobo, I., da Luz, F. Q., Hay, P., Gaeta, T. L., Teixeira, P. C., Cordás, T. A., ... & Salis, Z. (2023). Is binge eating associated with poor weight loss outcomes in people with a high body weight? A systematic review with meta-analyses. *Eating and Weight Disorders-Studies on Anorexia, Bulimia and Obesity*, *28*(1), 89. - Theodoulou, A., Hartmann‐Boyce, J., Gorenberg, J., Oke, J. L., Butler, A. R., Bastounis, A., ... & Aveyard, P. (2023). Weight regain and mental health outcomes following behavioural weight management programmes: A systematic review with meta‐analyses. *Clinical Obesity*, *13*(3), e12575. - Elperin, A. (2024). Supportive Interventions in Managing Eating Disorders: A Review of the Evolving Medical Professional Landscape. *Adv. Mind. Body. Med*, *28*, 15-19. - Chew, H. S. J., Lau, S. T., & Lau, Y. (2022). Weight‐Loss interventions for improving emotional eating among adults with high body mass index: A systematic review with Meta‐Analysis and Meta‐Regression. *European Eating Disorders Review*, *30*(4), 304-327. - Toledo, P. R., Lotufo-Neto, F., Verdeli, H., Goulart, A. C., Marques, A. H., de Oliveira Solis, A. C., & Wang, Y. P. (2023). Interpersonal psychotherapy for treatment of obesity: A systematic review and meta-analysis. *Journal of affective disorders*, *320*, 319-329. - LOPES NERI, L. D. C., Mariotti, F., Guglielmetti, M., Fiorini, S., Tagliabue, A., & Ferraris, C. (2024). Dropout in cognitive behavioural treatment (CBT) in adults with overweight: a systematic review. *OBESITY FACTS*, *17*, 571-572. - Gravina, D., Keeler, J. L., Akkese, M. N., Bektas, S., Fina, P., Tweed, C., ... & Himmerich, H. (2023). Randomized controlled trials to treat obesity in military populations: a systematic review and meta-analysis. *Nutrients*, *15*(22), 4778. - Neri, L. D. C. L., Mariotti, F., Guglielmetti, M., Fiorini, S., Tagliabue, A., & Ferraris, C. (2024). Dropout in cognitive behavioral treatment in adults living with overweight and obesity: a systematic review. *Frontiers in Nutrition*, *11*, 1250683. - Iturbe, I., & Maiz, E. (2022). Prevención de los trastornos de la conducta alimentaria desde las diferentes perspectivas psicológicas. *Nutrición Hospitalaria*, *39*(SPE2), 68-80. | Systematic review |
| - Presnell, K. E. (2005). *The effects of dieting as usual on weight change and bulimic pathology: experimental evidence*. The University of Texas at Austin. - Khazaal, Y., Fresard, E., Rabia, S., Chatton, A., Rothen, S., Pomini, V., ... & Zullino, D. (2007). Cognitive behavioural therapy for weight gain associated with antipsychotic drugs. *Schizophrenia research*, *91*(1-3), 169-177. - Laessle, R. G., Platte, P., Schweiger, U., & Pirke, K. M. (1996). Biological and psychological correlates of intermittent dieting behavior in young women. A model for bulimia nervosa. *Physiology & behavior*, *60*(1), 1-5. - Dorling, J. L., Bhapkar, M., Das, S. K., Racette, S. B., Apolzan, J. W., Fearnbach, S. N., ... & CALERIE Study Group. (2019). Change in self-efficacy, eating behaviors and food cravings during two years of calorie restriction in humans without obesity. *Appetite*, *143*, 104397. - Raber, A. C. (2004). *Empowering women: A health promotion program for weight-related problems*. Bowling Green State University. - de Menezes, M. C., Mingoti, S. A., Cardoso, C. S., de Deus Mendonça, R., & Lopes, A. C. S. (2015). Intervention based on Transtheoretical Model promotes anthropometric and nutritional improvements—A randomized controlled trial. *Eating behaviors*, *17*, 37-44. - Giner-Bartolome, C., Fagundo, A. B., Sanchez, I., Jimenez-Murcia, S., Santamaria, J. J., Ladouceur, R., ... & Fernandez-Aranda, F. (2015). Can an intervention based on a serious videogame prior to cognitive behavioral therapy be helpful in bulimia nervosa? A clinical case study. *Frontiers in psychology*, *6*, 982. - Mathisen, T. F., Sundgot-Borgen, J., Rosenvinge, J. H., & Bratland-Sanda, S. (2018). Managing risk of non-communicable diseases in women with bulimia nervosa or binge eating disorders: A randomized trial with 12 months follow-up. *Nutrients*, *10*(12), 1887. - Presnell, K., & Stice, E. (2003). An experimental test of the effect of weight-loss dieting on bulimic pathology: tipping the scales in a different direction. *Journal of abnormal psychology*, *112*(1), 166. - Dalai, S. S., Adler, S., Najarian, T., & Safer, D. L. (2018). Study protocol and rationale for a randomized double-blinded crossover trial of phentermine-topiramate ER versus placebo to treat binge eating disorder and bulimia nervosa. *Contemporary Clinical Trials*, *64*, 173-178. - Grilo, C. M., Lydecker, J. A., Jastreboff, A. M., Pittman, B., & McKee, S. A. (2023). Naltrexone/bupropion for binge‐eating disorder: A randomized, double‐blind, placebo‐controlled trial. *Obesity*, *31*(11), 2762-2773. - Marshall, R. D. (2023). *Examination of the Feasibility, Acceptability, and Effectiveness of a Group Program for Internalized Weight Bias Among Undergraduate Women* (Doctoral dissertation, University of Hawai'i at Manoa). - Rom, S., Miskovic‐Wheatley, J., Barakat, S., Aouad, P., Fuller‐Tyszkiewicz, M., & Maguire, S. (2022). Evaluating the feasibility and potential efficacy of a brief eTherapy for binge‐eating disorder: A pilot study. *International Journal of Eating Disorders*, *55*(11), 1614-1620. | Wrong population (age, BMI etc) |
| - White, M. A., & Grilo, C. M. (2013). Focus on Women’s Mental Health Bupropion for Overweight Women With Binge-Eating Disorder: A Randomized, Double-Blind, Placebo-Controlled Trial. *J Clin Psychiatry*, *74*(4), 400-406. - Appolinario, J. C., Godoy-Matos, A., Fontenelle, L. F., Carraro, L., Cabral, M., Vieira, A., & Coutinho, W. (2002). An open-label trial of sibutramine in obese patients with binge-eating disorder. *Journal of Clinical Psychiatry*, *63*(1), 28-30. - Appolinario, J. C., Fontenelle, L. F., Papelbaum, M., Bueno, J. R., & Coutinho, W. (2002). Topiramate use in obese patients with binge eating disorder: an open study. *The Canadian Journal of Psychiatry*, *47*(3), 271-273. - Resch, M., Jako, P., Sido, Z., & Haasz, P. (1999). The combined effect of psychotherapy and fluoxetine on obesity. *Orvosi Hetilap*, *140*(40), 2221-2225. - Bauer, C., Fischer, A., & Keller, U. (2006). Effect of sibutramine and of cognitive‐behavioural weight loss therapy in obesity and subclinical binge eating disorder. *Diabetes, obesity and metabolism*, *8*(3), 289-295. - Milano, W., Petrella, C., Casella, A., Capasso, A., Carrino, S., & Milano, L. (2005). Use of sibutramine an inhibitor of the reuptake of serotonin and noradrenaline, in the treatment of binge eating disorder: A placebo-controlled study. *Advances in therapy*, *22*, 25-31. - Smith, S. R., Blundell, J. E., Burns, C., Ellero, C., Schroeder, B. E., Kesty, N. C., ... & Weyer, C. (2007). Pramlintide treatment reduces 24-h caloric intake and meal sizes and improves control of eating in obese subjects: a 6-wk translational research study. *American Journal of Physiology-Endocrinology and Metabolism*, *293*(2), E620-E627. - Enzi, G. (1995). Dexfenfluramine Italian Multicentre Open Study (DIMOS) Efficacy and Safety of Dexfenfluramine in the Treatment of Patients with Simple or Complicated Obesity. *Clinical Drug Investigation*, *10*, 249-256. - Appolinario, J. C., Godoy-Matos, A., Fontenelle, L. F., Carraro, L., Cabral, M., Vieira, A., & Coutinho, W. (2002). An open-label trial of sibutramine in obese patients with binge-eating disorder. *Journal of Clinical Psychiatry*, *63*(1), 28-30. - Mathus-Vliegen, L. M. (1993). Dexfenfluramine influences dietary compliance and eating behavior, but dietary instruction may overrule its effect on food selection in obese subjects. *Journal of the American Dietetic Association*, *93*(10), 1163-1166. - Painot, D., Jotterand, S., Kammer, A., Guzman, M., Aubry, M., & Golay, A. (1998). Traitements des troubles du comportement alimentaire chez les patients obèses. *Médecine et hygiène*, *56*(2212), 1194-1197. - Grilo, C. M., Masheb, R. M., White, M. A., Gueorguieva, R., Barnes, R. D., Walsh, B. T., ... & Garcia, R. (2014). Treatment of binge eating disorder in racially and ethnically diverse obese patients in primary care: randomized placebo-controlled clinical trial of self-help and medication. *Behaviour research and therapy*, *58*, 1-9. | Medication not eligible |

References

1. Berk KA, Buijks HIM, Verhoeven AJM, et al. Group cognitive behavioural therapy and weight regain after diet in type 2 diabetes: results from the randomised controlled POWER trial. *Diabetologia* 2018; **61**(4): 790-9.

2. Grilo CM, Masheb RM, Salant SL. Cognitive behavioral therapy guided self-help and orlistat for the treatment of binge eating disorder: a randomized, double-blind, placebo-controlled trial. *Biol Psychiatry* 2005; **57**(10): 1193-201.

3. Wilfley DE, Welch RR, Stein RI, et al. A randomized comparison of group cognitive-behavioral therapy and group interpersonal psychotherapy for the treatment of overweight individuals with binge-eating disorder. *Arch Gen Psychiatry* 2002; **59**(8): 713-21.

4. Carels RA, Miller JC, Shonrock AT, Byrd R, Haley E. Exploring the addition of self-compassion skills training to a behavioral weight loss program delivered using video conferencing software. *Journal of Contextual Behavioral Science* 2021; **21**: 196-202.

5. Chao AM, Wadden TA, Walsh OA, et al. Effects of Liraglutide and Behavioral Weight Loss on Food Cravings, Eating Behaviors, and Eating Disorder Psychopathology. *Obesity (Silver Spring)* 2019; **27**(12): 2005-10.

6. da Luz FQ, Swinbourne J, Sainsbury A, et al. HAPIFED: a Healthy APproach to weIght management and Food in Eating Disorders: a case series and manual development. *J Eat Disord* 2017; **5**: 29.

7. Mensinger JL, Calogero RM, Tylka TL. Internalized weight stigma moderates eating behavior outcomes in women with high BMI participating in a healthy living program. *Appetite* 2016; **102**: 32-43.

8. DiMarco ID, Klein DA, Clark VL, Wilson GT. The use of motivational interviewing techniques to enhance the efficacy of guided self-help behavioral weight loss treatment. *Eat Behav* 2009; **10**(2): 134-6.

9. Grilo CM, Lydecker JA, Morgan PT, Gueorguieva R. Naltrexone + Bupropion Combination for the Treatment of Binge-eating Disorder with Obesity: A Randomized, Controlled Pilot Study. *Clin Ther* 2021; **43**(1): 112-22.e1.

10. Grilo CM, Masheb RM. A randomized controlled comparison of guided self-help cognitive behavioral therapy and behavioral weight loss for binge eating disorder. *Behav Res Ther* 2005; **43**(11): 1509-25.

11. Dassen FCM, Houben K, Van Breukelen GJP, Jansen A. Gamified working memory training in overweight individuals reduces food intake but not body weight. *Appetite* 2018; **124**: 89-98.

12. Grilo CM, White MA. Orlistat with behavioral weight loss for obesity with versus without binge eating disorder: randomized placebo-controlled trial at a community mental health center serving educationally and economically disadvantaged Latino/as. *Behav Res Ther* 2013; **51**(3): 167-75.

13. Grilo CM, White MA, Masheb RM, Ivezaj V, Morgan PT, Gueorguieva R. Randomized controlled trial testing the effectiveness of adaptive "SMART" stepped-care treatment for adults with binge-eating disorder comorbid with obesity. *Am Psychol* 2020; **75**(2): 204-18.

14. Loader KA. A compassionate mind approach to self-help for treatment seeking obese adults: A randomised controlled trial: University of Leicester; 2013.

15. Nauta H, Hospers H, Kok G, Jansen A. A comparison between a cognitive and a behavioral treatment for obese binge eaters and obese non-binge eaters. *Behavior Therapy* 2000; **31**(3): 441-61.

16. Grilo CM, Masheb RM, Wilson GT, Gueorguieva R, White MA. Cognitive-behavioral therapy, behavioral weight loss, and sequential treatment for obese patients with binge-eating disorder: a randomized controlled trial. *J Consult Clin Psychol* 2011; **79**(5): 675-85.

17. Masheb RM, Grilo CM, Rolls BJ. A randomized controlled trial for obesity and binge eating disorder: low-energy-density dietary counseling and cognitive-behavioral therapy. *Behav Res Ther* 2011; **49**(12): 821-9.

18. Munsch S, Biedert E, Meyer A, et al. A randomized comparison of cognitive behavioral therapy and behavioral weight loss treatment for overweight individuals with binge eating disorder. *Int J Eat Disord* 2007; **40**(2): 102-13.

19. Preuss H, Pinnow M, Schnicker K, Legenbauer T. Improving Inhibitory Control Abilities (ImpulsE)-A Promising Approach to Treat Impulsive Eating? *Eur Eat Disord Rev* 2017; **25**(6): 533-43.

20. Ramirez EM, Rosen JC. A comparison of weight control and weight control plus body image therapy for obese men and women. *J Consult Clin Psychol* 2001; **69**(3): 440-6.

21. Rock CL, Flatt SW, Sherwood NE, Karanja N, Pakiz B, Thomson CA. Effect of a Free Prepared Meal and Incentivized Weight Loss Program on Weight Loss and Weight Loss Maintenance in Obese and Overweight Women: A Randomized Controlled Trial. *JAMA* 2010; **304**(16): 1803-10.

22. Barnes RD, Ivezaj V, Martino S, Pittman BP, Grilo CM. Back to Basics? No Weight Loss from Motivational Interviewing Compared to Nutrition Psychoeducation at One-Year Follow-Up. *Obesity (Silver Spring)* 2017; **25**(12): 2074-8.

23. Moss EL, Tobin LN, Campbell TS, von Ranson KM. Behavioral weight-loss treatment plus motivational interviewing versus attention control: lessons learned from a randomized controlled trial. *Trials* 2017; **18**(1): 351.

24. Werrij MQ, Jansen A, Mulkens S, Elgersma HJ, Ament AJ, Hospers HJ. Adding cognitive therapy to dietetic treatment is associated with less relapse in obesity. *J Psychosom Res* 2009; **67**(4): 315-24.

25. Wilson GT, Wilfley DE, Agras WS, Bryson SW. Psychological treatments of binge eating disorder. *Arch Gen Psychiatry* 2010; **67**(1): 94-101.

26. Allison KC, Chao AM, Bruzas MB, et al. A pilot randomized controlled trial of liraglutide 3.0 mg for binge eating disorder. *Obes Sci Pract* 2023; **9**(2): 127-36.

27. Grilo CM, Lydecker JA, Fineberg SK, Moreno JO, Ivezaj V, Gueorguieva R. Naltrexone-Bupropion and Behavior Therapy, Alone and Combined, for Binge-Eating Disorder: Randomized Double-Blind Placebo-Controlled Trial. *Am J Psychiatry* 2022; **179**(12): 927-37.

28. Grammer AC, Monterubio GE, D'Adamo L, et al. Evaluation of a combined, online intervention for binge-type eating disorders and high body weight in young adults. *Eat Behav* 2023; **50**: 101789.

29. da Luz FQ, Mohsin M, Teixeira PC, et al. A Comparison of the Efficacy of Online HAPIFED versus Online Cognitive Behavioural Therapy for Binge Eating Disorder: A Randomized Controlled Trial. *Obesities*, 2024. (accessed.

30. Rahimi Ardabili H, Reynolds R, Zwar N, Briggs N, Vartanian L. The efficacy of an online behavioural intervention for improving dietary habits with a focus on self-compassion, goal-setting and self-monitoring: A randomised controlled trial; 2023.

31. Calugi S, Ruocco A, El Ghoch M, et al. Residential cognitive-behavioral weight-loss intervention for obesity with and without binge-eating disorder: A prospective case-control study with five-year follow-up. *Int J Eat Disord* 2016; **49**(7): 723-30.

32. Carbone EA, Caroleo M, Rania M, et al. An open-label trial on the efficacy and tolerability of naltrexone/bupropion SR for treating altered eating behaviours and weight loss in binge eating disorder. *Eat Weight Disord* 2021; **26**(3): 779-88.

33. Dalle Grave R, Calugi S, Bosco G, et al. Personalized group cognitive behavioural therapy for obesity: a longitudinal study in a real-world clinical setting. *Eat Weight Disord* 2020; **25**(2): 337-46.

34. Abilés V, Rodríguez-Ruiz S, Abilés J, et al. Effectiveness of cognitive-behavioral therapy in morbidity obese candidates for bariatric surgery with and without binge eating disorder. *Nutr Hosp* 2013; **28**(5): 1523-9.

35. Pataky Z, Carrard I, Gay V, et al. Effects of a Weight Loss Program on Metabolic Syndrome, Eating Disorders and Psychological Outcomes: Mediation by Endocannabinoids? *Obes Facts* 2018; **11**(2): 144-56.

36. Barnes RD, Ivezaj V, Martino S, Pittman BP, Paris M, Grilo CM. Examining motivational interviewing plus nutrition psychoeducation for weight loss in primary care. *J Psychosom Res* 2018; **104**: 101-7.

37. Carbone EA, Caroleo M, Rania M, et al. Influence of NUCB/Nesfatin-1 Polymorphism on Treatment Response to Naltrexone/Bupropion SR in Binge Eating Disorder and Obesity. *Biomedicines* 2024; **12**(2).

38. Mohseni M, Kuckuck S, Meeusen REH, et al. Improved Physical and Mental Health After a Combined Lifestyle Intervention with Cognitive Behavioural Therapy for Obesity. *Int J Endocrinol Metab* 2023; **21**(1): e129906.

39. Brownell KD, Center LE. The LEARN Program for Weight Management: Lifestyle, Exercise, Attitudes, Relationships, Nutrition: American Health Publishing Company; 2004.

40. Cooper Z, Fairburn CG, Hawker DM. Cognitive-Behavioral Treatment of Obesity: A Clinician's Guide: Guilford Publications; 2004.

41. The Diabetes Prevention Program (DPP): description of lifestyle intervention. *Diabetes Care* 2002; **25**(12): 2165-71.

42. Group DPPR. Reduction in the incidence of type 2 diabetes with lifestyle intervention or metformin. *New England journal of medicine* 2002; **346**(6): 393-403.

43. McCallum S. Gamification and serious games for personalized health. *Stud Health Technol Inform* 2012; **177**: 85-96.

44. Houben K, Dassen FCM, Jansen A. Taking control: Working memory training in overweight individuals increases self-regulation of food intake. *Appetite* 2016; **105**: 567-74.

45. Houben K, Wiers RW, Jansen A. Getting a Grip on Drinking Behavior: Training Working Memory to Reduce Alcohol Abuse. *Psychological Science* 2011; **22**(7): 968-75.

46. Davidson MH, Hauptman J, DiGirolamo M, et al. Weight control and risk factor reduction in obese subjects treated for 2 years with orlistat: a randomized controlled trial. *Jama* 1999; **281**(3): 235-42.

47. Goss K. The Compassionate Mind Approach to Beating Overeating: Little, Brown; 2019.

48. Foster GD, Wyatt HR, Hill JO, et al. A randomized trial of a low-carbohydrate diet for obesity. *N Engl J Med* 2003; **348**(21): 2082-90.

49. Devlin MJ, Goldfein JA, Petkova E, et al. Cognitive Behavioral Therapy and Fluoxetine as Adjuncts to Group Behavioral Therapy for Binge Eating Disorder. *Obesity Research* 2005; **13**(6): 1077-88.

50. Ello-Martin JA, Roe LS, Ledikwe JH, Beach AM, Rolls BJ. Dietary energy density in the treatment of obesity: a year-long trial comparing 2 weight-loss diets. *Am J Clin Nutr* 2007; **85**(6): 1465-77.

51. Hilbert A, Tuschen-Caffier B. Essanfälle und Adipositas: ein Manual zur kognitiv-behavioralen Therapie der Binge-Eating-Störung;[mit CD-ROM]: Hogrefe; 2010.

52. Government of Canada. Eating well with Canada’s Food Guide. <http://www.hc-sc.gc.ca/fn-an/alt_formats/hpfb-dgpsa/pdf/food-guide-aliment/view_eatwell_vue_bienmang-eng.pdf> (2011).

53. Miller WR, Rollnick S. Motivational interviewing: Helping people change: Guilford press; 2012.

54. Rollnick S, Miller WR, Butler CC, Aloia MS. Motivational interviewing in health care: helping patients change behavior. Taylor & Francis; 2008.

55. DiLillo V, Siegfried NJ, West DS. Incorporating motivational interviewing into behavioral obesity treatment. *Cognitive and Behavioral Practice* 2003; **10**(2): 120-30.
